# An efficient end-to-end computational framework for the generation of ECG calibrated volumetric models of human atrial electrophysiology

**DOI:** 10.1016/j.media.2025.103822

**Published:** 2025-10-10

**Authors:** Elena Zappon, Luca Azzolin, Matthias A.F. Gsell, Franz Thaler, Anton J. Prassl, Robert Arnold, Karli Gillette, Mohammadreza Kariman, Martin Manninger, Daniel Scherr, Aurel Neic, Martin Urschler, Christoph M. Augustin, Edward J. Vigmond, Gernot Plank

**Affiliations:** a Division of Biophysics, Medical University of Graz, Graz, Austria; b BioTechMed-Graz, Graz, Austria; c NumeriCor Gmbh, Graz, Austria; d Institute of Visual Computing, Graz University of Technology, Graz, Austria; e Institute for Medical Informatics, Statistics and Documentation, Medical University of Graz, Graz, Austria; f Scientific Computing and Imaging Institute, University of Utah, USA; g Department of Biomedical Engineering, University of Utah, USA; h Clinical Department of Cardiology, Medical University of Graz, Graz, Austria; i University of Bordeaux, CNRS, Bordeaux, France

**Keywords:** Atrial electrophysiology, Volumetric atrial models, Cardiac digit twins, Universal atrial coordinates, Cardiac modeling

## Abstract

Computational models of atrial electrophysiology (EP) are increasingly utilized for applications such as the development of advanced mapping systems, personalized clinical therapy planning, and the generation of virtual cohorts and digital twins. These models have the potential to establish robust causal links between simulated *in silico* behaviors and observed human atrial EP, enabling safer, cost-effective, and comprehensive exploration of atrial dynamics. However, current state-of-the-art approaches lack the fidelity and scalability required for regulatory-grade applications, particularly in creating high-quality virtual cohorts or patient-specific digital twins. Challenges include anatomically accurate model generation, calibration to sparse and uncertain clinical data, and computational efficiency within a streamlined workflow.

This study addresses these limitations by introducing novel methodologies integrated into an automated end-to-end workflow for generating high-fidelity digital twin snapshots and virtual cohorts of atrial EP. These innovations include: (i) automated multi-scale generation of volumetric biatrial models with detailed anatomical structures and fiber architecture; (ii) a robust method for defining space-varying atrial parameter fields; (iii) a parametric approach for modeling inter-atrial conduction pathways; and (iv) an efficient forward EP model for high-fidelity electro-cardiogram (ECG) computation.

We evaluated this workflow on a cohort of 50 atrial fibrillation (AF) patients, producing high-quality meshes suitable for reaction-eikonal and reaction–diffusion models, demonstrating the ability to efficiently simulate atrial ECGs under parametrically controlled conditions, and, as a proof-of-concept, the feasibility of calibrating models to clinical P-wave in four patients. These advancements represent a critical step towards scalable, precise, and clinically applicable digital twin models and virtual cohorts, enabling enhanced patient-specific predictions and therapeutic planning.

## 1. Introduction

Computational models of atrial EP are increasingly being considered in a variety of applications, ranging from the industrial development of devices such as electro-anatomical mapping (EAM) systems (Reddy et al., 2023; Szili-Torok et al., 2023) to the stratification and planning of clinical therapy (Boyle et al., 2019). These applications are built upon the mechanistic nature of biophysical models of atrial EP, and are based on the tacit assumption that simulated *in silico* behaviors closely correspond to the real human atrial EP observed in patients. If sufficient regulatory-strength evidence of such a close causal relation is provided, *in silico* models can be used to explore human atrial EP more comprehensively, in a safer and more cost-effective manner compared to the current paradigm based on preclinical animal testing and clinical trials (Dössel et al., 2012; Schotten et al., 2011). However, current state-of-the-art models supporting evidence of such a tight causal and quantitatively accurate relation are limited. The vast majority of computational studies use simplified anatomical models (Roney et al., 2023; Azzolin et al., 2023; Labarthe et al., 2014) with uncalibrated default parameters (Boyle et al., 2019; Sakata et al., 2024), and any comparison to directly observable quantities, such as ECG, is usually limited (Loewe et al., 2016; Ferrer et al., 2015) or even not accounted for (Roney et al., 2023). This can be largely attributed to the limited capabilities of current modeling technologies in terms of anatomical model generation as well as calibration and simulation technologies. A direct comparison to observations would reveal major discrepancies between physical and virtual spaces, thus undermining the credibility of the model.

The degree of fidelity and detail needed in modeling atrial anatomies and structures, as well as the metrics used for measuring it, is application-dependent (Boyle et al., 2019; Bhagirath et al., 2024). Beyond the minimum requirement of a mechanistic relation – that is, the models of atrial EP are able to qualitatively recapitulate all the mechanisms of atrial EP at play – in silico models can be calibrated to be representative of a group of patients of interest, also covering anatomical and functional variability (Niederer et al., 2020). Such representation of a patient cohort, rather than an individual, may facilitate generic interpretation of observations in real physical atria, and prediction of EP responses to therapeutic interventions. Such sets of functionally-similar models referred to as virtual cohorts, have recently started to be considered for in silico trials and for safety and efficacy testing of new devices or therapies (Viceconti et al., 2020). Nevertheless, creating virtual cohorts of in silico models will necessitate a shift in cardiac modeling, moving from a limited set of custom models to efficient and scalable workflows capable of generating large volumes of models quickly (Niederer et al., 2020).

Most demanding are clinical applications geared towards precision cardiology, that is, to tailor therapies to individual patients. There, models are sought that replicate cardiac anatomy and structures and quantitatively calibrate to match functional observations from an individual patient’s heart in a one-to-one manner. Such functionally equivalent models, where a particular stimulus or perturbation leads to the same emergent response in virtual and real space at a single time point, are referred to as digital twin snapshots. When the models are continuously or periodically updated with measurements, they become true digital twins (Corral-Acero et al., 2020; Hopman et al., 2023; Bhagirath et al., 2024).

However, the ability of current atrial EP modeling pipelines for creating high-fidelity digital twin snapshots or virtual cohorts at scale in a sufficiently efficient and robust manner is severely limited. The key challenge is to describe the electrical sources in the atria accurately enough to predict the electrical potential field in its surroundings where all observable measurements are recorded. In humans *in vivo*, electrical measurements are in the form of electrograms (EGMs) by devices or EAM systems, or as ECG at the body surface. While conceptually simple, the implementation of such a pipeline is vastly demanding, posing a long list of formidable challenges many of which remain unaddressed. In general, modeling pipelines for creating cardiac digital twin models of atrial EP are separated into two distinct stages, an anatomical and a functional twinning stage (Roney et al., 2023; Azzolin et al., 2023; Labarthe et al., 2014).

At the anatomical stage, multi-label segmentation of tomographic images is performed to identify all relevant domains (Payer et al., 2017) which are turned into multi-label computational meshes (Prassl et al., 2009; Crozier et al., 2016) to accurately represent biatrial anatomy. Involved procedures are notoriously laborious, requiring numerous manual operator interventions by trained experts and significant computational resources (Roney et al., 2023; Azzolin et al., 2023), to obtain anatomically accurate representations of sufficient mesh quality for a given type of cardiac EP simulation. Specifically for atrial anatomies, these are often simplified (David M. Harrild, 2000) and represented as manifolds only (Roney et al., 2023; Azzolin et al., 2023; Labarthe et al., 2014), thus limiting the achievable quantitative accuracy in representing electrical sources and associated potential fields.

At the functional twinning stage, the fundamental core challenge of calibrating an atrial EP model to clinical data is to infer high-dimensional space-varying parameter fields governing the EP behavior from limited sparse clinical recordings that are afflicted with substantial observational uncertainties (Coveney et al., 2022; Whittaker et al., 2020). This poses a number of technological problems. Model functionalization refers to conceiving a framework for comprehensively describing a sufficiently high-dimensional parameter space defined over geometrically complex objects, such as the heart (Bayer et al., 2018; Roney et al., 2019a), that encapsulates all relevant factors governing atrial EP, and the genesis of the associated extracellular potential field. These fields must be exposed to unattended algorithmic manipulation to facilitate parameter sweeps in order to minimize the mismatch between simulated and observed data within an optimization procedure (Grandits et al., 2021, 2024b). A computationally efficient yet accurate forward EP model for generating the observed electrical recordings, i.e. ECGs, EGMs or EAMs, is required to cope with the computational burden of a large number of model evaluations incurring during optimization (Neic et al., 2017b; Pezzuto et al., 2017; Gillette et al., 2021b). Computational workflows meeting these criteria and reported so far in the literature (Boyle et al., 2019; Roney et al., 2023; Azzolin et al., 2023), are primarily limited in terms of anatomical and structural representation, functional calibration capabilities, as well as automation, efficiency, robustness, reproducibility, and numerical accuracy.

In this study, we address these limitations by developing novel methodologies that are essential for the scalable generation of atrial digital twin snapshots and virtual cohorts, calibrated by ECGs and, potentially, EGMs or EAMs. These comprise:

- An automated flexible multi-scale approach for generating anatomically accurate volumetric biatrial models with comprehensive parametric incorporation of atrial structures and fiber architecture, and of sufficiently high mesh quality to be suitable for most widely used forward EP models;
- A robust method for generating a volumetric atrial reference frame for defining space-varying atrial parameter fields, and their unattended manipulation in optimization sweeps;
- A novel method for the flexible parametric representation of IC pathways;
- An efficient and clinically-compatible ECG forward model that computes high fidelity gold standard ECGs and EGMs with close to real-time performance.

We report on the integration of these methodological developments into a highly automated and efficient end-to-end workflow for building high-fidelity digital twin snapshots of atrial EP from clinical data. We elucidate in detail the methodological underpinnings of all processing stages – from image analysis over model generation to ECG and EGM prediction – and evaluate the performance of the end-to-end workflow on a clinical cohort of patients treated for AF, by processing 50 patient datasets randomly selected from the Graz AF registry. For each patient, both coarse and fine-resolution meshes were generated at ≈ 0.90 mm and 0.25 mm for reaction-eikonal (Neic et al., 2017b) and reaction–diffusion mono- or bidomain models, respectively, together with a set of automatically generated volumetric UAC. We demonstrate the ability to compute atrial ECGs by performing sweeps on parametrically-controlled and easy-to-generate inter-atrial pathways, and we show the possibility of automatically varying the position and shape of the SAN, allowing fast calibration of the model at hands. Furthermore, as proof-of-principle we demonstrate feasibility of calibrating atrial EP models to clinical P-waves under normal sinus rhythm activation in a set of four patients, by employing a Saltelli sampling approach for optimizing 24 selected parameters governing atrial activation.

The structure of the paper is as follows: Section 2 presents a comprehensive overview of the proposed end-to-end workflow, encompassing the anatomical twinning stage (Section 2.1) and the functional modeling stage (Section 2.2). Specifically, our approach for generating anatomically accurate volumetric biatrial models, including anatomical structures and fibers, is detailed in Sections 2.1.1–2.1.4. Sections 2.1.5 and 2.1.6 describe the computation of the UAC and torso model surrounding the biatrial mesh. Our novel method for computing interatrial connections is outlined in Section 2.2.1, while the parameter sweeping process for model calibration is detailed in Section 2.2.2. The numerical results are provided in Section 3, with the discussion, limitations, and conclusions presented in Sections 4, 5, and 6, respectively.

## 2. Methods

**Fig. 1.**
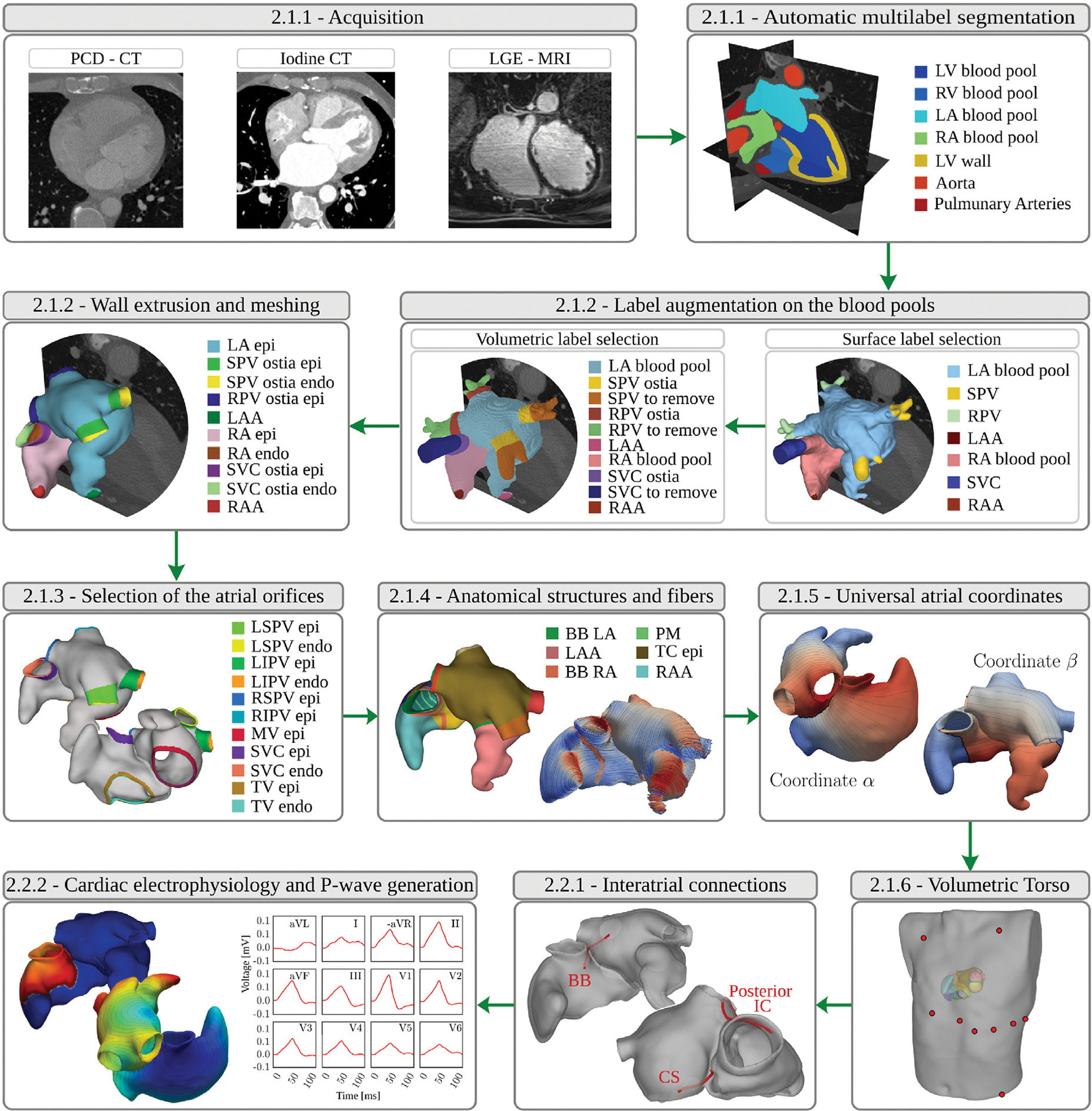
Schematic outline of the end-to-end framework for the generation of ECG-calibrated volumetric models of patient-specific human atria. After image acquisition, the workflow comprises nine steps: automatic multilabel segmentation, automatic label augmentation on the blood pools, extrusion of the volumetric bilayer walls, automatic selection of atrial orifices, automatic annotation of anatomical structures and fiber generation, generation of UAC, registration and generation of a torso volume conductor, generation of ICs, and cardiac electrophysiological simulation and P-wave generation. We moreover provide the paper section index where each step is detailed.

We provide a schematic overview of an end-to-end workflow for the generation of anatomically accurate volumetric biatrial-torso model in Fig. 1.

The workflow comprises two major processing stages, (i) an anatomical twinning stage comprising the generation of the atrial anatomical model with structural labels and fiber architecture, the generation of an atrial reference frame for spatial parameter swiping, and a torso anatomical model, and (ii) a functional modeling stage, for the definition of inter-atrial pathways, and the setting up of a forward EP model for representing and calibrating atrial action potential propagation, and for generating the associated clinically observable signals, such as the ECGs and the EGMs. Both stages are closely linked to the choice of forward EP mathematical model, which influences, for instance, the targeted spatial mesh resolution of the biatrial anatomical model.

The anatomical twinning stage is organized following an almost automatic and sequential step approach:

- image segmentation: by exploiting a convolutional neural network, tomographic scans, either computed tomography (CT) or magnetic resonance imaging (MRI), are automatically segmented;
- label augmentation on the atrial blood pool: veins and appendages on the atrial blood pools are identified and labeled, for defining the border between atrial myocardium and attached vasculature;
- extrusion of the volumetric atrial walls: by accounting for the identified labels on the blood pools, a sequence of prescribed operations is employed on the segmentation to extrude the endo- and epicardial walls endowed with a selected thickness, automatically opening veins and valves, and generating a corresponding smooth volumetric mesh;
- selection of atrial orifices: a fully automated process is carried out to identify the complete set of vein ostia and valves on the atrial walls, separated in endocardial and epicardial tissue;
- automatic labeling of anatomical structures and fiber generation: the remaining anatomical regions are automatically annotated, and the atrial fiber architecture is computed;
- UACs generation: a system of normalized volumetric universal coordinates is computed;
- integration of a torso volumetric conductor: a reference torso anatomical model is automatically integrated with the biatrial anatomical model and lead fields are computed for all considered electrode positions for computing all clinical ECG signals of interest.

The functional modeling stage comprises:

- (viii) generating ICs: a flexible set of conduction pathways that cannot be delineated from images are computed to facilitate inter-atrial impulse conduction
- (ix) generate EP clinical outputs: employing either R-D monodomain formulation, or a R-E model to represent the transmembrane voltage, $V_{m}$, in the atrial model, a parameter spaces of EP parameter is selected to be used for the model calibration. The P-wave of the 12-lead ECGs on the torso are computed using the respective lead fields (Potse, 2018; Gillette et al., 2022). The same approach can be potentially employed to compute EGMs in the blood pools.

The entire workflow is implemented in a single user-friendly software building on the package meshtool (Neic et al., 2020) and on python code. For meshing operations the software TetGen (Weierstrass Institute, Berlin, Germany) and NetGen (CerbSim GmbH, Vienna, Austria) have been licensed and integrated with meshtool. An interactive mode is moreover available, allowing for the verification of the labeling process, the visual control of all processing stages, and the correction of any potential software errors, through the use of the software NumeriCor Studio (Numericor GmbH, Graz, Austria). All required interactive steps are readily supported by the freely available starter version of NumeriCor Studio for academia. Each step of our workflow will be extensively described in the following sections.

### 2.1. Anatomical twinning stage

#### 2.1.1. Imaging data acquisition and segmentation

For developing and testing the workflow, 50 patients diagnosed with AF and scheduled for AF ablation therapy were selected. Iodanized contrast CT scans were acquired at an isotropic resolution of 0.40mm as part of the routine standard of care at the Medical University of Graz hospital for patients included in the local AF ablation registry. This registry was approved by the ethics committee of the Medical University of Graz (reference number 26–217 ex 13/14) and all patients gave written informed consent.

Each CT dataset was segmented using the SpatialConfiguration-Net (SCN) (Thaler et al., 2021), an automated multi-label segmentation method based on convolutional neural networks. Prior to input to the SCN, 3D image stacks were cropped around the center of the heart, re-sampled to an isotropic resolution of 1.20mm, and Gaussian filtered with σ=1.00mm. The SCN was trained to recognize and label seven cardiac domains, comprising all four major cardiac left ventricle (LV), right ventricle (RV), LA, RA blood pools, the LV myocardium, and the vascular blood pools in the aorta and the pulmonary artery. Segmentation quality was assessed interactively, to ascertain topological soundness. Topological errors, if present, such as connections between the blood pools of the LA appendage and the left superior pulmonary vein (LSPV), or between the CS and the LA were identified, and manually corrected using the ITK-SNAP software (Yushkevich et al., 2006).

#### 2.1.2. Automated label augmentation on the blood pools

Label augmentation is performed to identify the atrial appendages and veins, which will later be used in the wall extrusion process. Among all the volumetric labels defined through segmentation, only the LA and RA blood pools are targeted at this stage, while all other anatomical labels serve as auxiliary references.

The blood pool label augmentation is carried out in two stages: the first stage automatically defines key landmarks on the surface of the blood pools; the second stage effectively labels the entire blood pool volumes.

**Fig. 2.**
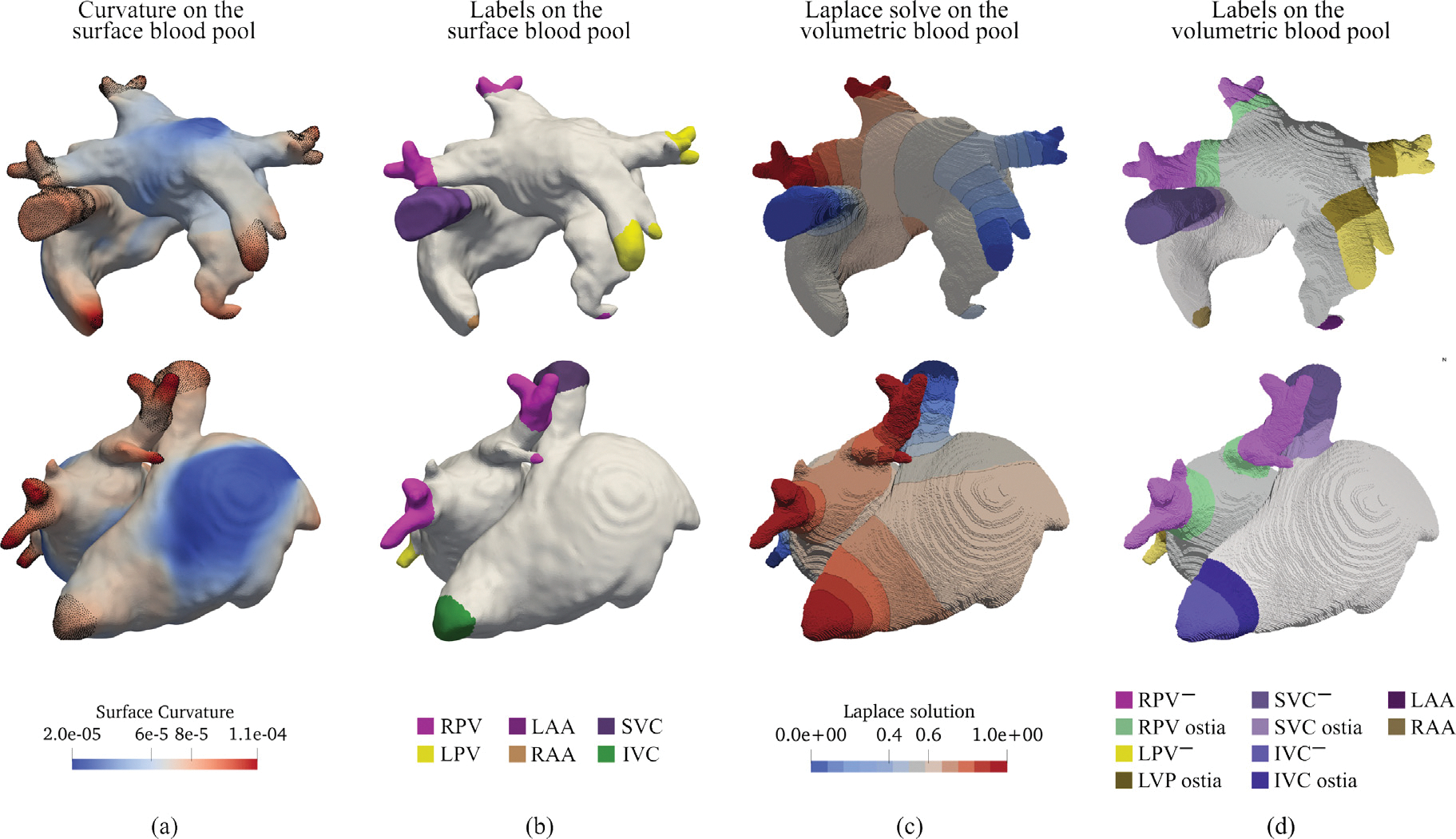
(a) Point-wise curvature on the blood pool surface mesh. The figure also highlights the correlation between the surface region of high curvature and anatomical structures to mark. (b) Minimal set of labels selected based on surface curvature, including LPV, RPV and LAA on the LA, and IVC, SVC and RAA on the RA. (c) Instance of LD solution and isosurfaces on the blood pool volumetric mesh. (d) Vein ostiae and the discardable tissue labeled on the blood pool volumetric mesh.

In the first stage, beginning with the segmentation, triangular surface meshes of the LA and RA blood pools are generated. Key landmarks to identify include the LPV, RPV, and the LAA in the LA, and the SVC, IVC, CS, and RAA in the RA. The automatic landmarking process is based on the computation of the curvature of the surface mesh, as presented in Azzolin et al. (2023) (see Fig. 2(a)). The curvature is defined point-wise, allowing for clustering of the mesh nodes based on curvature thresholding. Clusters of nodes with the highest curvature – five on the LA and four in the RA – are considered for anatomical structure selection and marked using the following algorithm (see Fig. 2(b)):

- **LA:** The first landmark selected is the vertex of the LAA, identified as the central point of the LA cluster of node closest to the ascending aorta, or, if the aorta is not labeled, as the cluster with the highest curvature. A point cloud algorithm based on principal component analysis (PCA) is then employed to divide the remaining clusters into two groups: the two clusters nearest to the LAA are designated as LPV, while the others are identified as RPV.
- **RA:** Similarly to the LA, the vertex of the RAA is first selected as the central point of the RA cluster closest to the ascending aorta or the one with the highest curvature. PCA is then applied to the two largest clusters in terms of nodes to identify the vena cavae. The SVC is designated as the cluster nearest to the RAA, while the other is marked as IVC. Among the remaining clusters with the highest curvature, the one nearest to the IVC is labeled as CS.

The second stage entails the generation of a volumetric hexahedral mesh of LA and RA blood pools from the initial volumetric segmentation of the SCN. For each atrium, a series of LD problems is solved to define a set of measures of distance between the previously defined veins and appendages. Dirichlet boundary conditions are generated by projecting the landmarks defined on the blood pool surface mesh onto the volumetric mesh (see Fig. 2(c)). Isosurfaces of the LD solves are then employed to label the veins ostiae according to prescribed thresholds. The remaining portions of the veins are considered discardable tissue, as they will not be part of the final anatomical model. Therefore, they are marked for removal. Hereon, we will define this landmark by employing the name of the vessel followed by a negative sign, e.g. the portion of discardable tissue of the SVC will be denoted as SVC^−^ (see Fig. 2(d)). The same procedure is also applied to the CS. The labels defined on the volumetric hexahedral mesh are finally projected back onto an image stack that will then contain all augmented fine-grained labeling information.

The high variability in the atrial anatomy occasionally led the automatic algorithm to mislabel some structures. Specifically, errors in the positioning of the IVC and CS were witnessed, or in the identification of a reasonable portion of discardable IVC blood pool volume. In such cases, the workflow visualization modality allows for a swift manual correction.

As the atrial walls are thin, their accurate segmentation from current routine clinical imaging with CT and MRI is, in general, not feasible (Bishop et al., 2016; Dewland et al., 2013). In our workflow, we therefore use *a priori* knowledge (Azzolin et al., 2020; Whitaker et al., 2016; Varela et al., 2017; Beinart et al., 2011) on the RA and LA endo- and epicardial width to construct the atrial walls. A two steps process is applied. First, atrial walls are grown by image operations applied to the image stack, followed by a mesh generation step to create conformal unstructured meshes with smooth boundaries.

Using the augmented volumetric segmentation, atrial walls are grown by imposing a series of rule-based extrusion operations at the interface of two different landmarks, or at the interface of marked tissue with the image background. Extrusion operations may comprise both growth in an inward direction, *i.e.* erosion, or outward, corresponding to negative and positive growth along the surface normal of the labeled domain. The spatial granularity of extrusion is prescribed in physical length units, i.e. mm, which is converted to discrete steps corresponding to the voxel size of 0.40mm of the segmented image.

Specifically, volumetric representations of the atrial walls are then generated by the following rules: (i) an inclusion set of labels where interfaces between differently labeled domains are allowed to grow into each other, comprising the blood pool labels of RA, and LA, the ostiae of both caval veins, SVC and IVC, and the pulmonary veins, RPV and LPV; (ii) an exclusion set comprising domains labeled as not pertaining to the atrial blood pool, to impede extrusion along these interfaces. This set includes the LV and RV blood pools, the CS, the SVC^−^, IVC^−^, as well as right and left pulmonary veins, RPV^−^, LPV^−^ (see Fig. 2). The extrusion operations are performed then on the inclusion set, with the exclusion set serving as boundaries that impede any further growth. First, an inward extrusion is performed to create a minimal endocardial surface layer, followed by an outward dilation to thicken the endocardial layer to the prescribed width, and a further outward extrusion to create an epicardial layer. Distinct values for the thickness of the of LA and RA endocardial and epicardial layers, as well as the wall width of each vein can be specified independently. As the exclusion set impedes any inward or outward extrusion, growth is then blocked at the interfaces of RA and RV, and LA and LV blood pools, respectively, to create the orifices of tricuspid valve (TV) and mitral valve (MV). Similarly, atrial walls are not grown at the interface of the RA and LA blood pool with the tissue of the veins and CS to be dismissed, thus effectively creating the openings of all in- and outflow anatomical structures. Additional rules may be applied to mitigate effects due to limited segmentation accuracy. For instance, the growth of the epicardial wall may be constrained around the ostiae of the LSPV in cases where LAA and the LSPV are in close proximity, to avoid merging.

**Fig. 3.**
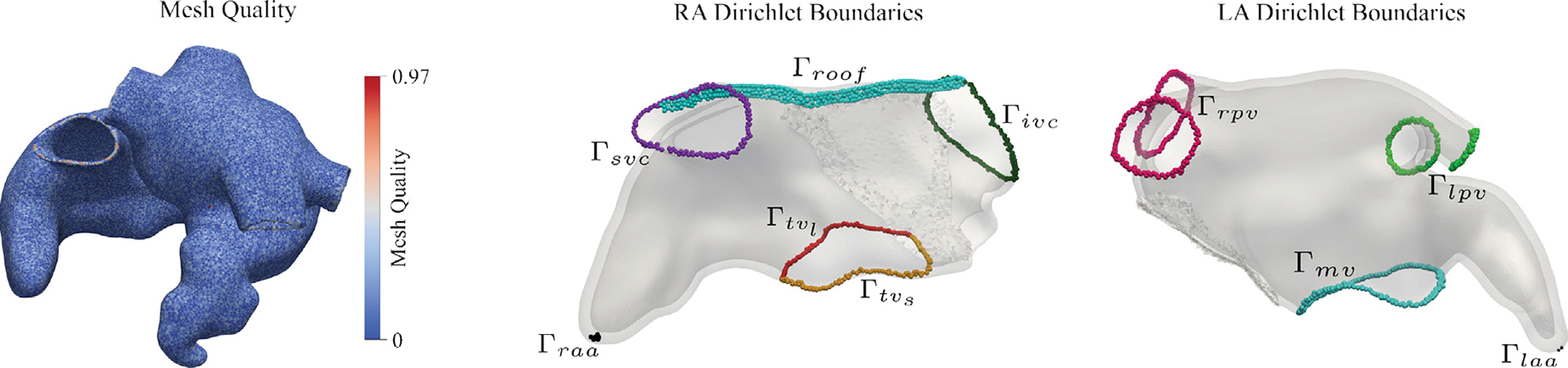
Left: Mesh quality computed on the biatrial model. Right: Boundaries on the RA and LA 15 LD problems employed to determine atrial anatomical structures and tissues known to have different EP properties.

After the extrusion phase, the walls of both atria are volumetrically defined on the augmented image stack. The generation of a volumetric anatomical mesh starts with extracting a surface mesh that encloses all atrial wall labels. This initial surface mesh, conforming to a jagged voxel representation, is subjected to several processing steps to obtain a smooth representation of all atrial walls, with smooth transitions between wall segments of different widths. Automatic correction of intersecting elements, dangling triangles or co-localized points that may arise during meshing, surface smoothing to mitigate terracing effects due to voxelation in clinical images of limited resolution, and mesh quality checks are implemented to ascertain topological correctness and enhanced mesh quality. Finally, the surface mesh is resampled to match a prescribed target resolution. Typically, for R-E models, a resolution of 0.90mm, sufficient to resolve endo- and epicardial atrial wall layers, was selected. For R-D simulation, a higher resolution of 0.25mm was used to resolve slowly propagating wavefronts without producing numerical artifacts (Bishop and Plank, 2025). As a final step, volumetric meshes of the atrial walls are created by the inward meshing of the labeled surfaces, again followed by an automatic correction of intersecting elements that may arise during meshing, reindexing, refinement, and mesh quality-enhancing procedures. The mesh quality q of the final volumetric mesh is computed following the approach described in Karabelas et al. (2018), based on metrics introduced in Batdorf et al. (1997), Knupp (2022), Kanchi and Masud (2007) (see Fig. 3-left). Specifically, the global mesh quality is defined as the average of the quality of individual elements $q_{e}$, where

$$q_{e}=1-\frac{V_{e}}{V},$$

with $V_{e}$ denoting the volume of the element e, and V representing the volume of an equilateral element with the same average edge length as element e.

Thus, at the end of this stage, the biatrial anatomy is represented by an unstructured volumetric tetrahedral mesh endowed with basic anatomical labels, including endo- and epicardial domains of RA, LA, RPV, LPV, IVC and SVC.

#### 2.1.3. Automated selection of the atrial orifices

The atrial orifices comprising LPV, RPV, MV, TV, SVC, IVC, and CS are individually labeled as separated anatomical entities. Specifically, following Azzolin et al. (2023), orifices are detected as mesh nodes of the biatrial surface that are shared between adjacent elements with disjoint endo- or epicardial labels of the same anatomical structure. Each orifice is therefore represented as a cluster Γ of mesh nodes arranged along a ring, and is readily identified based on anatomical labels, except for the differentiation into superior and inferior of the RPV and LPV. There, each entity, LSPV, LIPV, right superior pulmonary vein (RSPV), RIPV, consists of four clusters which are discriminated based on the Euclidian distances between their centroid and those of the tips of the LAA. For instance, if the LAA is available, the cluster with its centroid closest to the LAA is labeled as LSPV. Then, between the remaining unclassified clusters, the one with its centroid closest to the LSPV is defined as LIPV. The RSPV is identified as the remaining cluster with centroid nearest to the LAA, which allows to label the last cluster as LIPV. Given the identified orifices, the anatomical regions of the LA labeled as RPV and LPV are updated to separate the inferior and superior pulmonary veins.

**Fig. 4.**
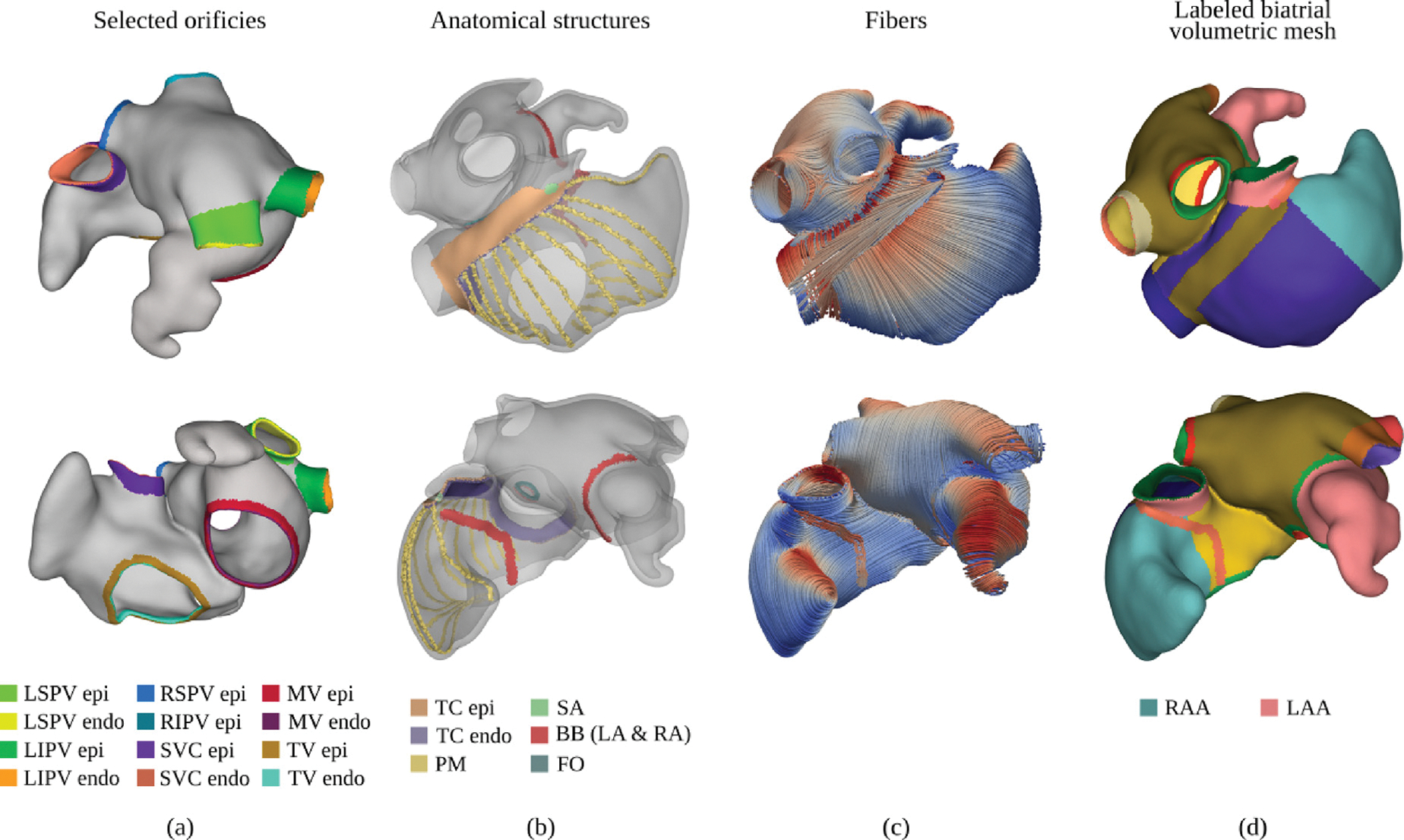
(a) Identification and labeling of the atrial orifices. Different labels are used for endocardial and epicardial tissue layers. (b) Anatomical structures identified on the biatrial anatomy, including TC, SAN, PMs, FO, and part of the BB. (c) Generated rule-based atrial fiber architecture. (d) Final biatrial volumetric mesh, enriched with anatomical structures by merging the labels produced in stages (a) and (b).

At this stage, the sets of nodes spanning the orifices $\Gamma_{ivc},\;\Gamma_{svc},\;\Gamma_{tv},\;\Gamma_{cs}$ on the RA, and $\Gamma_{rpv}=\Gamma_{ripv}\cup \Gamma_{rspv},\;\Gamma_{lpv}=\Gamma_{lpv}\cup \Gamma_{lpv}$, and $\Gamma_{mv}$, are known and can be used as boundary conditions (see Fig. 3 for a graphical representation of the identified clusters), along with the corresponding anatomical regions, as represented in Fig. 4(a).

#### 2.1.4. Automated labeling of anatomical structures and fiber generation

Following Zheng et al. (2021), Azzolin et al. (2023), a further refined sub-classification of anatomical structures is performed on the RA and LA walls to define anatomical structures and tissue regions of known differences in EP behaviors. Such structures – including the SAN, TC and PMs, the BB, and the muscular rim of the FO, as well as RAA and LAA – are automatically annotated on a per-rule basis defining location and width of the sought-after anatomical structures (refer to Fig. 4b).

The implemented rules are based on solutions of multiple instances of LD problems on the domain $\Omega_{\mathrm{myo}}$, i.e. the right $\Omega_{RA}$ or left $\Omega_{LA}$ atrium, with varying boundary conditions. Specifically, given a generic unknown φ, this involves solving

$$\left\{\begin{array}{ll} -\Delta \varphi =0 & \mathrm{in}\;\Omega_{\mathrm{myo}},\\ \varphi =\varphi_{i} & \mathrm{on}\;\Gamma_{i},i=1,\ldots ,m\\ \nabla \varphi \cdot n=0 & \mathrm{on}\;\Gamma_{n}, \end{array}\right.$$

for m=2,3,4 suitable DBC $\varphi_{i}\in R$, set on generic partitions of the atria boundaries $\Gamma_{i}$, and Neumann boundary conditions $\Gamma_{n}$, such that $\cup_{i=1}^{m}\Gamma_{i}\cup \Gamma_{n}=\partial \Omega_{myo}$. The LD solutions are used as measures of distance between the orifices and/or, the appendages. Moreover, a plane passing through the center of the IVC and SVC, and the center of mass of the RA is computed to separate the RA into lateral and septal regions. Hereon, we will call the intersection of such plane with the RA wall as RA roof. Similarly, the TV annulus is partitioned into a lateral and a septal tricuspid valve annulus, $\Gamma_{tv_{l}}$ and $\Gamma_{tv_{s}}$, respectively. A representation of the defined set of boundaries is shown in Fig. 3.

Compared to previous work (Zheng et al., 2021; Azzolin et al., 2023), three additional LD solutions were computed on the RA: (i) $\varphi_{v3}$ with DBCs $\varphi_{1}=0$ and $\varphi_{2}=1$ imposed at the rings of SVC and IVC, respectively; (ii) $\varphi_{r2}$ with $\varphi_{1}=0$ at the rings of the SVC and IVC, and on the RA roof, and $\varphi_{2}=1$ at the ring of the MV; and (iii) $\varphi_{w2}$ with DBCss $\varphi_{1}=0$ on the roof, $\varphi_{2}=1$ and $\varphi_{3}=-1$ on the septal and lateral parts, respectively, at the MV annulus. The complete set of DBCs is summarized in Table 1. These additional solutions are designed to improve the definition of the TC and PMs.

The labeling of the BB follows Zheng et al. (2021). Using the additional solutions, we marked the area of the exit site of the SAN, and, optionally, the muscular rim of the FO, and the anterior-central band of the BB. The SAN is defined as a sphere with a radius of 2.50mm and centered on the node closest to the SVC with minimum $\varphi_{v2}$. The FO is defined as an annulus with thickness of 2.00mm, located in the center of the atrial septum. The start and end points of the anterior-central band of the BB are selected as two points in the middle of the BB lateral bands of both RA and LA. Additionally, an intermediate point is chosen as the epicardial node of the LA nearest to the midpoint of the RA lateral BB. A geodesic path is, moreover, created using Dijkstra’s algorithm between the starting and intermediate points, and between the intermediate and end points. Around this path, a region of 2.00mm width is marked as BB. Unlike in Zheng et al. (2021), Azzolin et al. (2023), no additional volumetric mesh is added to define the central bundle of the BB. While our workflow allows for marking the complete BB, in this work, we replace the anterior-central band of the BB, as well as all other inter-atrial conduction pathways, by conducting cables (see Section 2.2.1), increasing the flexibility of the modeling approach.

Finally, the fiber architecture is computed using the rule-based method presented in Piersanti et al. (2021), including the enhancements previously reported in Zheng et al. (2021), Azzolin et al. (2023). A graphical overview of all anatomical structures, tissues, and fibers, is given in Fig. 4.

#### 2.1.5. Universal atrial coordinates generation

**Fig. 5.**
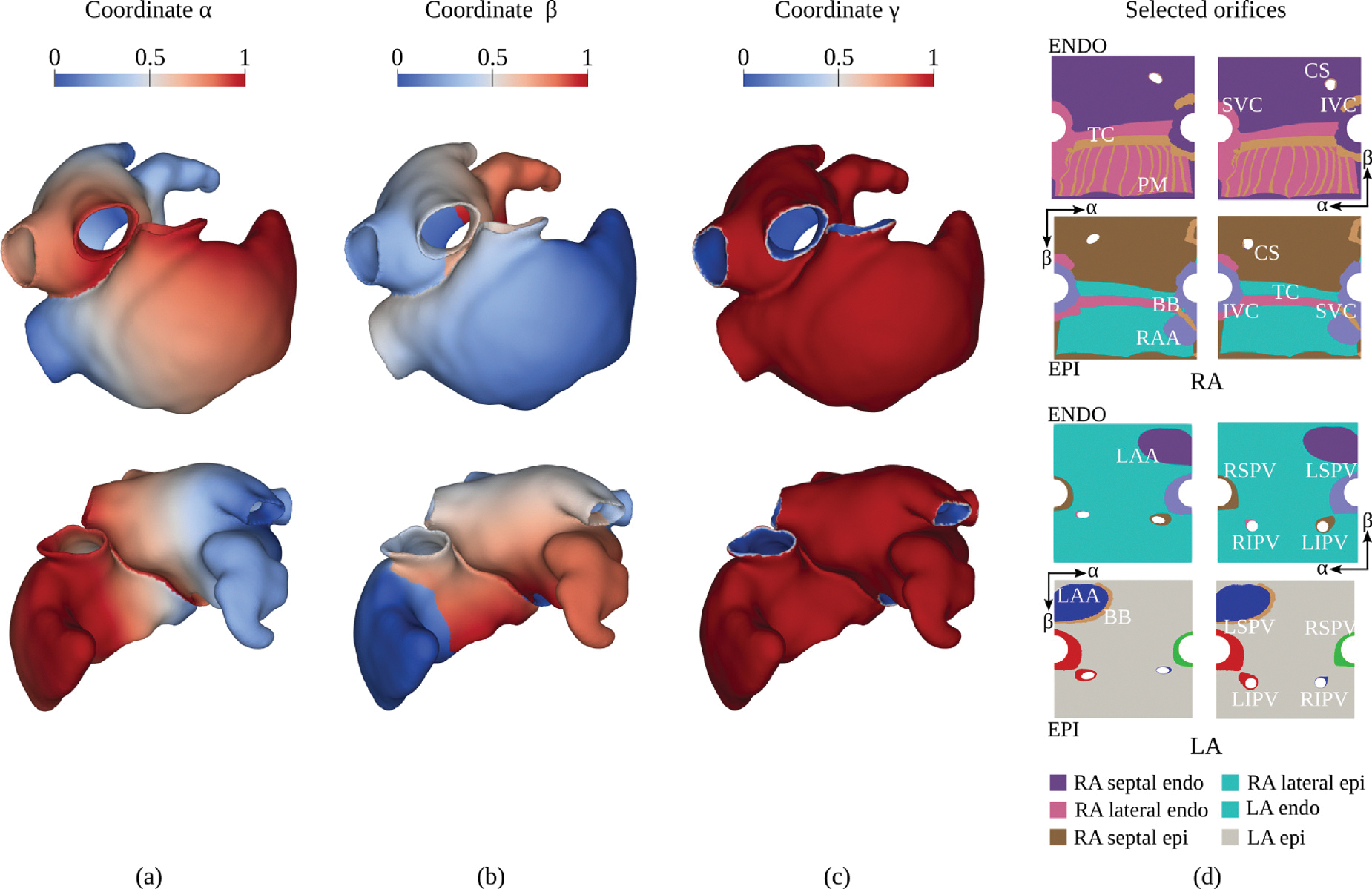
(a) Distribution of the coordinate α, representing the SVC-to-IVC coordinate for the RA, and the lateral-to-septal coordinate for the LA. (b) Distribution of the coordinate β, representing the lateral-to-septal coordinate for the RA, and the posterior-to-anterior coordinate for the LA. (c) Distribution of the coordinate γ, representing the endocardial-to-epicardial coordinate for both atria. (d) Projection of the labels on the space generated by the UAC before (left) and after (right) the solution of the linear elasticity problem. The colors represent the projection on the UAC space of the anatomical structures labeled in the atrial model represented in Fig. 4.

To compute the UACs directly on the volumetric models we expand upon the method outlined in Roney et al. (2021, 2023). For each atrium, we define four coordinates: (i) $\alpha_{RA}$, the IVC-to-SVC coordinate on the RA, and $\alpha_{LA}$, the lateral-to-septal coordinate on the LA (see illustration in Fig. 5(a)); (ii) $\beta_{RA}$, the lateral-to-septal coordinate on the RA that starts at the lateral TV annulus, runs through the roof, and ends at the septal TV annulus, and $\beta_{LA}$, the posterior-to-anterior coordinate on the LA that starts at the posterior MV annulus, runs through the roof, and ends at the anterior MV annulus (see Fig. 5(b)); (iii) $\gamma_{RA}$ and $\gamma_{LA}$, the relative transmural distance between endocardium and epicardium in RA and LA, respectively (see Fig. 5(c)); (iv) a binary value encoding to which atrium the other coordinates belong, always assigning shared points at the LA–RA interface to the LA. To compute the four coordinates, for each atrium, we first solve three LD problems, imposing DBC at pre-defined boundary surfaces. Secondly, to ascertain equal locations of the CS, LIPV, and RIPV in the UAC space across different atrial models, a linear elasticity problem is solved.

**Fig. 6.**
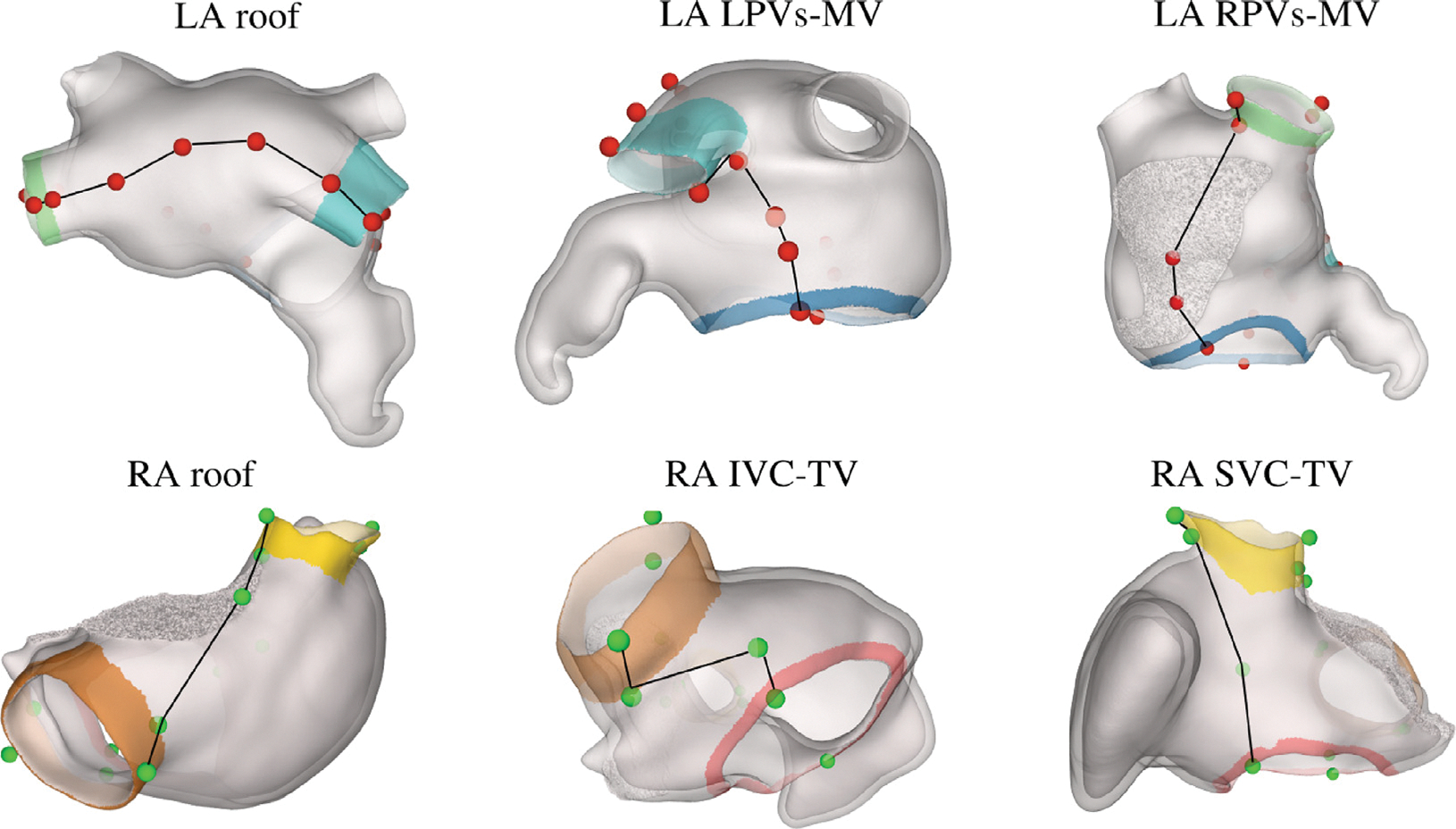
Set of reproducible points selected on the LA and RA to define the boundaries for the UAC computation.

The selection of the boundary surfaces is fully automated and based on the anatomical labels. Specifically, the marked atrial orifices are utilized to define a set of reproducible points on both the vessel openings and the atrial walls. Such points are selected according to the general shape of the atria for both LA and RA, to follow the roof of the RA, between the SVC and the IVC, and of the LA, between the LSPV and RSPV, and to set lateral and septal boundaries in both atria, as depicted in Fig. 6. Specifically, for the LA, five points are selected between the LSPV and the RSPV, two of which corresponding to the intersection of the LSPV and RSPV ostiae with the LA body, and the others following the LA roof. For the lateral and septal boundaries, two reference points are first identified on the MV ring. These correspond to the intersections of a plane — defined by the center of the MV, the RSPV, and the LSPV - with the MV ring. These points serve as anchors to connect the boundaries to the RSPV and LSPV. On the LSPV side, 3 more points are selected: one at the base of the LSPV ostium (i.e., the intersection of the LSPV ostium with the LA body), and two additional points along the lateral LA wall, extending posteriorly past the LAA, following the anatomical shape of the LA. Similarly, on the septal side near the RSPV, we define three further points: one at the intersection of the RSPV ostium with the LA body, and two more tracing the shape of the septal wall, connecting back to the MV. For the RA, the boundary connecting the SVC and the IVC is constructed by selecting three points: two at the intersections of the SVC and the IVC ostiae with the RA body, and an additional point that follows the general curvature of the RA roof. To define the remaining RA anterior/posterior boundaries between the SVC/IVC and the TV, two initial points are selected on the TV ring, analogous to the MV method. These correspond to the intersections of the TV ring with a plane defined by the centers of the TV, IVC, and SVC. Then, two intermediate points are defined between the IVC and one of the selected TV points: one at the intersection of the IVC ostium with the RA body, and one that traces the general contour of the posterior RA wall. From the SVC to the RV, two more points are selected: one at the intersection of the SVC ostium with the RA body, and another between this point and the previously defined point on the TV. Lastly, all nodes of the RAA are considered part of the posterior RA wall, which may potentially modify the final definition of this boundary.

**Fig. 7.**
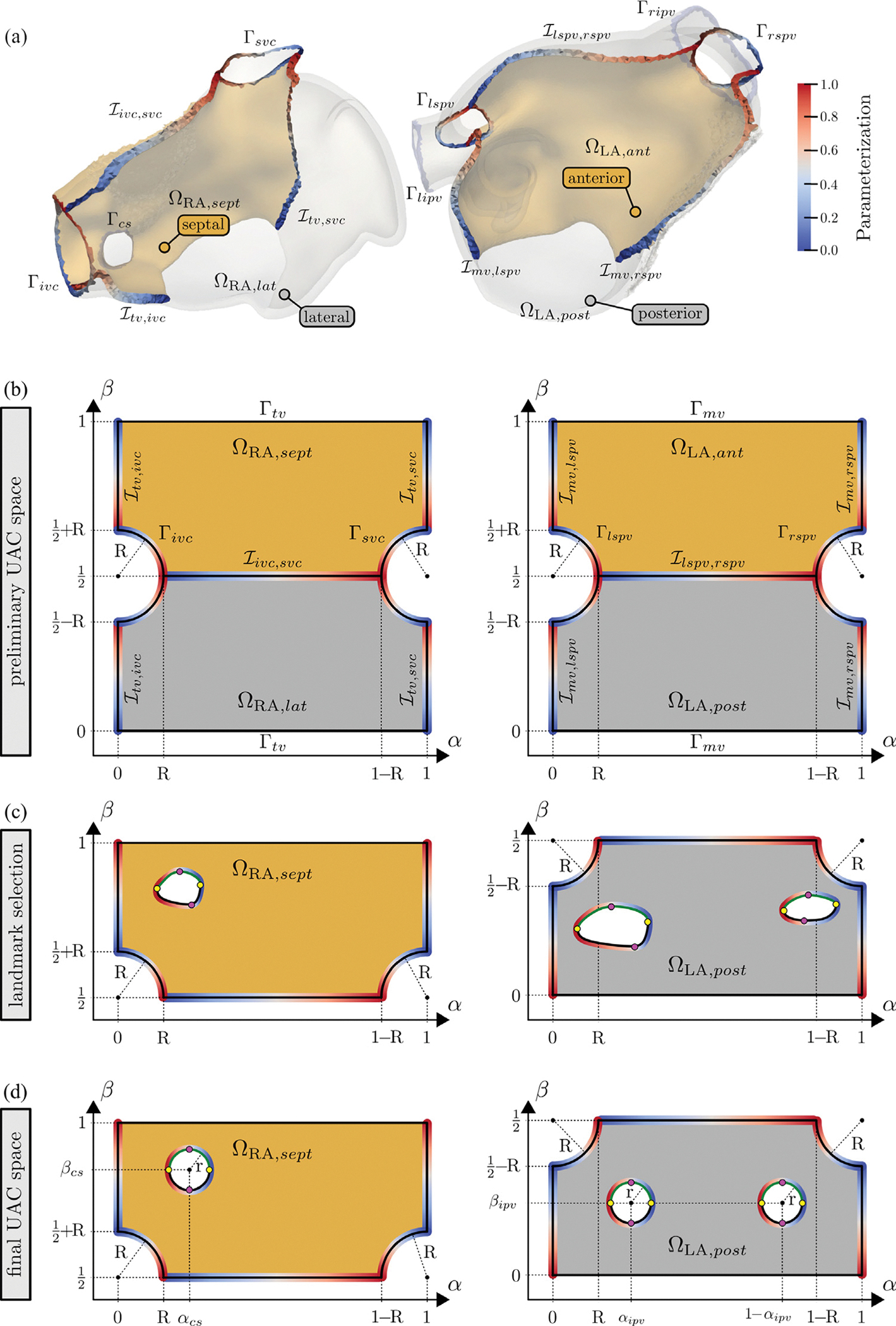
(a) Interfaces and boundary surfaces in the RA and LA and their parametrization. (b) Dirichlet values at the boundaries and interfaces used to compute the preliminary α and β components for the RA and LA are shown. (c) Selected landmarks and the parametrization of the CS, the LIPV, and the RIPV. (d) Final α and β components after moving the orifices. The color are coded from blue =0 to red =1.

The points are then connected with quadrilateral surfaces. The intersections of such surfaces with the atrial volumetric walls define six auxiliary surface meshes, three for each atrium, effectively representing the interfaces separating the RA into a septal $\Omega_{RA,\mathrm{sept}}$ and a lateral $\Omega_{RA,\mathrm{lat}}$ part, and the LA in an anterior $\Omega_{LA,\mathrm{ant}}$ and a posterior $\Omega_{\mathrm{LA},\mathrm{post}}$ part. Moreover, to avoid splitting of the appendages, the RAA is assigned to the lateral side of the RA, and the LAA is assigned to the anterior side of the LA. Therefore, for the RA we obtain two interfaces, $I_{\mathrm{tv},\mathrm{ivc}}$ and $I_{\mathrm{tv},\mathrm{svc}}$, from the TV to the IVC and SVC, respectively, and one interface ${ℐ}_{ivc,svc}$ along the roof from IVC to SVC. Similarly, the two interfaces ${ℐ}_{mv,lspv}$ and ${ℐ}_{mv,rspv}$, from the MV to the LSPV and RSPV, respectively, are computed for the LA, together with the interface ${ℐ}_{\mathrm{lspv},\mathrm{rspv}}$ along the roof from LSPV to RSPV. The defined interfaces are furthermore used to partition the TV, IVC, and SVC into septal and lateral parts, and the MV, LSPV, and RSPV into anterior and posterior (see Fig. 7 for a graphical representation). Such partition will be used in the following when solving the linear elasticity problem.

Both the interfaces and the atrial openings are then parameterized with a value s∈[0,1] with a Bi-Eikonal approach (Schuler et al., 2021). The α and β coordinates are computed by solving LD problems on the partitioned $\Omega_{RA}$ and $\Omega_{LA}$, with DBC assigned based on s, as defined in Table 2. For the γ component, only two Dirichlet boundary conditions are imposed, 0 at the endocardium and 1 at the epicardium.

The linear elasticity problem is solved in the UAC space, by projecting the first three coordinates onto two thin cuboids, one for each atrium, where α and β are described along the edges of the square face, and γ represents the thickness of the cuboid. The IVC, SVC, RSPV, and LSPV are moreover recast in circumferential form, on the edges of the square face representing the β coordinate. The square faces of each cuboid are then representing the endocardial and epicardial surfaces of the atrial walls. An updated Lagrangian formulation is then used to solve a linear elasticity problem, prescribing rule-based Dirichlet boundary conditions for the CS, RIPV, and LIPV. To achieve this, the parametrization of the CS, RIPV, and LIPV is carried out as follows. Beginning with the mesh nodes of each orifice, i.e., $\Gamma_{\mathrm{ripv}},\;\Gamma_{\mathrm{lipv}}$, and $\Gamma_{cs}$ as defined in Section 2.1.3, a node selection is grown across the biatrial surface in all directions. The outermost selected nodes form two rings, one on the endocardium and one on the epicardium, which enclose a transmural surface for each orifice. For each ring, four points are identified, corresponding to the minimum and maximum values of the α and β components and for each of the four endocardial-epicardial point pairs, the shortest transmural path is extracted on the corresponding transmural surface. The CS node selection is then divided into a roof and a TV section and parameterized using a LD solve, with Dirichlet boundary conditions applied to the eight previously extracted paths. Similarly, the LIPV and RIPV node selections are divided into a roof and a MV section and parameterized in the same manner. The final position of the CS, RIPV, and LIPV after convergence of the linear elasticity problem is defined in Table 3 with $R\;=\;0.1,\;r\;=\;0.04,\;\alpha_{cs}=0.2,\;\beta_{cs}=0.8,\;\alpha_{ipv}=0.25$, and $\beta_{ipv}=0.25$ and in Fig. 5(d), where the anatomical labels are also reported.

#### 2.1.6. Torso volume conductor model

**Fig. 8.**
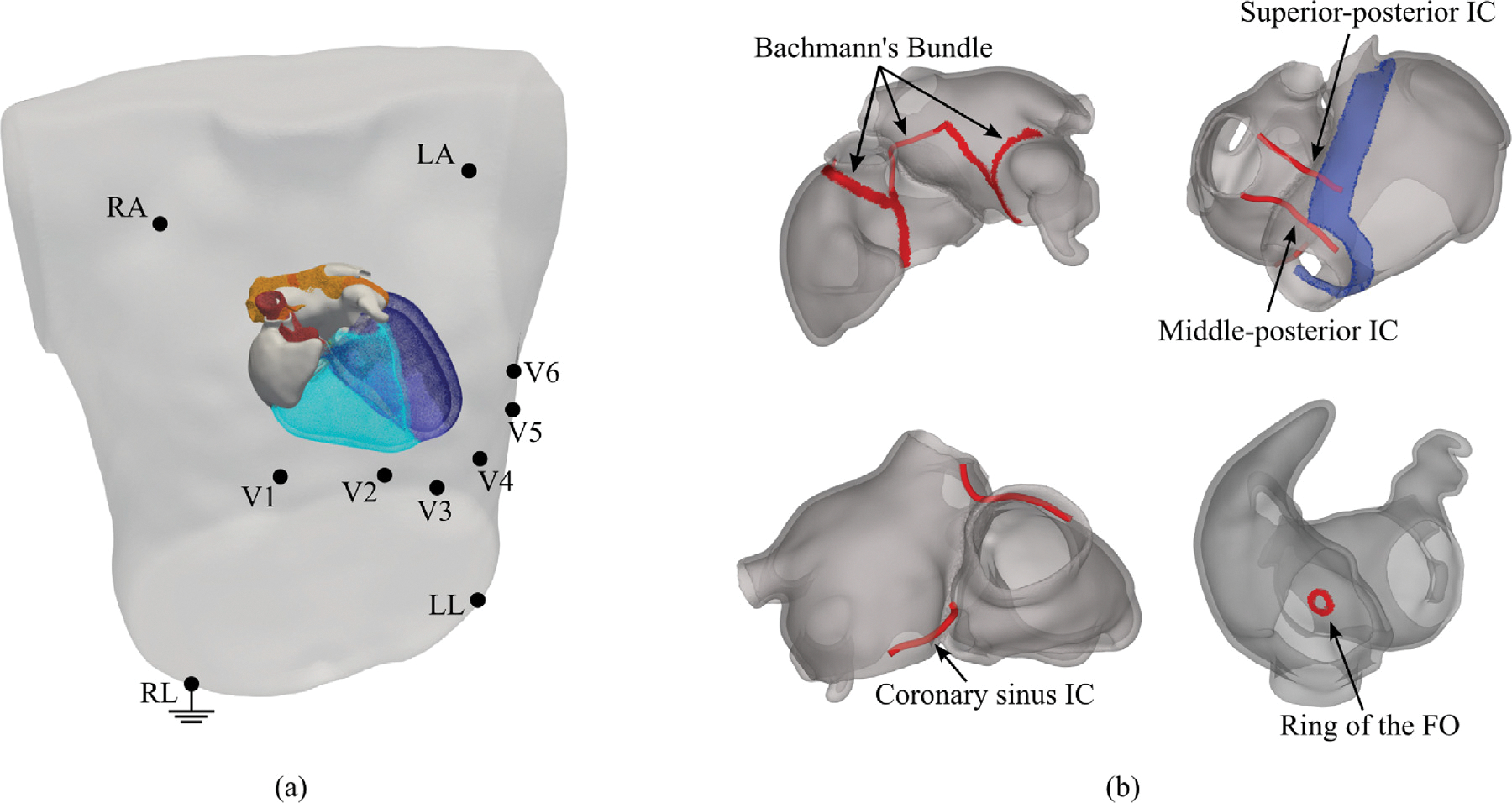
(a) Combined conformal atria-torso mesh generated by registering a patient atrial model, generated with our workflow, to a selected template torso model with positioned ECG electrodes. The ventricles for the template heart are also represented. (b) Representation of the electrical connections between the RA and LA, including four IC and the FO.

Since most clinical observations of atrial EP are obtained by sampling the potential fields generated within the volume conductor, such as ECGs or EGMs, volumetric models must be embedded in a torso domain to enable the prediction of these observations. However, the field of view of the CT scans of the patients within the Graz AF registry was restricted to the heart to minimize the patient’s exposure to X-ray radiation. Thus a full torso view including all ECG recording sites was not available. Thus, to predict atrial P-waves in the ECG, an automated procedure was implemented for registering a template heart-torso model with known electrode locations around the generated atria. The template was selected from a previously generated virtual cohort (Gillette et al., 2021a, 2022). More precisely, the atrial surfaces of the template were registered to the generated patient models by employing a global optimal 3D iterative closest point (GO-ICP) algorithm (Yang et al., 2013, 2015). The transformation was then stored and applied to the surface of the template torso along with the known positions of the electrodes (Gillette et al., 2021a, 2022) (see Fig. 8(a)).

A volumetric mesh was subsequently generated between the surfaces of the torso and the atria, then merged with the volumetric mesh of the atria. For simplicity, other organs represented in the template model, such as the lungs, were excluded to avoid potential meshing issues caused by intersections with the atrial geometry.

The obtained atria-torso model is suitable for the accurate simulation of ECGs and EGMs, either based on a bidomain model for simulating the entire extracellular potential field, $\phi_{e}(x,t)$ (Neic et al., 2017b; Bishop and Plank, 2011a; Zappon et al., 2024), or on a lead field approach (Geselowitz, 1989; Potse, 2018; Multerer and Pezzuto, 2021), yielding time traces of the extracellular potential sampled at discrete points $\phi_{e,x}(t)$.

### 2.2. Functional modeling stage

#### 2.2.1. Parametric modeling of inter-atrial connections

Action potential propagation between RA and LA is limited to a few electrically excitable muscular strands of tissue called ICs, that serve as conduction pathways between the otherwise electrically insulated atria. Known pathways include BB, the sheath of the CS, the muscular rim of the FO and, posteriorly, a superior-posterior and a middle-posterior bridge. Histologically, ICs arise sub-epicardially and branch outside of the atrial walls (van Campenhout et al., 2013; Platonov et al., 2002). While the BB (Bachmann, 1916; Lemery et al., 2003; van Campenhout et al., 2013) and the CS musculature (Antz et al., 1998a; Chauvin et al., 2000) have been shown to be the most relevant electrical IC (Sakamoto et al., 2005; Knol et al., 2019), the specific number and location of the complete set of IC remains unclear (Platonov et al., 2002; Sakamoto et al., 2005; Platonov, 2007).

In this study, we considered all five aforementioned ICs as the only electrical connections between the RA and LA. Except for the rim of the FO, which forms an electrically conductive bridge within the volumetric biatrial mesh, RA and LA are electrically fully decoupled using a nodal splitting approach (Costa et al., 2014). All other ICs traversing the space outside the atrial walls are modeled as discrete electrically conducting strands, that are anchored within the RA and the LA at given sites defined through UACs (see Fig. 8(b)), and constrained to the sub-epicardium. These strands are defined as auto-generated cables as previously utilized in Gillette et al. (2022), and their electrical behavior is modeled with the approach used to define the His-Purkinje System (HPS) in the ventricles in Vigmond and Clements (2007), Neic et al. (2017b). The cables used to define the ICs were generated along the shortest path connecting the anchoring location in RA and LA, respectively.

Of the five represented ICs, the BB is composed of both an intra- and an inter-atrial component, where the intra-atrial component is modeled as tissue embedded within the atrial epicardial wall, as described in Section 2.1.4, and only the inter-atrial central component is modeled as a cable. The BB emerges at the junction of the RA body and the SVC, in close proximity to the sino-atrial node (SAN), and runs in an anterior direction around the RAA, where it branches into two bands, one towards the RA vestibule, and the inter-atrial central BB running towards the anterior LA wall, anchoring in the LA epicardium. From there another intra-atrial band runs epicardially towards the LAA, encircles the LAA, and terminates superiorly between the LAA and the LSPV, and inferiorly, at the MV annulus (van Campenhout et al., 2013).

The two posterior ICs were defined as in Wachter et al. (2015), Loewe et al. (2016). The superior-posterior bridge emerges in proximity of the IVC at the 90% of the TC, and connects the RA with the LA wall near the RIPV, on the septal side. The middle-posterior IC starts at the junction of the IVC with the atrial body, on the TC, and ends at the junction of the RIPV, on the inferior side of the RIPV.

The musculature of the CS was represented by a single cable providing a pathway from the RA wall, near the CS ostium, to the posterior LA at the annulus of the MV. The origin of the cable in the RA was chosen superior to the CS, at a distance of 3.50mm, that is included between the 3 and 8.00mm range defined in Chauvin et al. (2000) from the CS ostium, while the LA anchoring site was selected according to Wachter et al. (2015).

The muscular rim of the FO was modeled as tissue using previously defined labels, endowed with slow conduction properties (Padala et al., 2021; Kharbanda et al., 2019; Harrild and Henriquez, 2000).

#### 2.2.2. Baseline calibration of the atrial activation sequence and P-wave simulation

The genesis of the atrial P-wave is governed by the spatio-temporal evolution of electrical depolarization wavefronts traversing the atria. At each point in the atria, the propagation of the wavefronts is governed by (i) a conduction velocity tensor that is determined by intra- and extracellular conductivities along the eigenaxes, (ii) the tissue composition condensed into a scalar bidomain surface-to-volume ratio, and (iii) the tissue’s EP properties related to cellular dynamics. Beyond these intrinsic conduction properties, the site of initiation of a depolarization wavefront, typically at an exit site of the SAN or at a pacing site, and the location of anchoring sites of inter-atrial conduction pathways and their associated conduction velocities influence the distribution of electrical sources during depolarization and, thus, the morphology of the P-wave. As such, the parameter vector controlling atrial activation is high dimensional. While all these parameters are exposed for unattended manipulation in parameter sweeps, their calibration as a whole is computationally not feasible with current technology, and, further, observable data are very sparse and insufficient to constrain the calibration procedure. As such, we only demonstrate the ability of our fully parametric 3D biatrial model to sweep over selected key parameters to generate physiologically meaningful sets of atrial P-waves.

Our workflow universally supports all common EP modeling approaches that build on the volumetric representation of atrial electrical sources, including all R-D mono- and bidomain models (Potse et al., 2006; Bishop and Plank, 2011a), as well as variants of R-E models (Neic et al., 2017b), including plain Eikonal models with $V_{m}$-recovery (Pezzuto et al., 2017). To demonstrate the feasibility of a sampling-based model calibration using the P-wave, we chose two popular approaches, the computationally expensive R-D monodomain model, requiring a high average spatial resolution of Δx≈0.25mm, and a lightweight R-E model at Δx≈0.90mm. Both models are combined with the lead field approach for the P-wave computation. We refrain here from providing a mathematical model formulation and refer to previous studies where all methodological underpinnings of the forward EP modeling have been described in detail (Bishop and Plank, 2011a; Neic et al., 2017b; Gillette et al., 2021b). All simulations are executed using CARPentry (Neic et al., 2017b) for R-D and R-E simulation runs, respectively, as well as openCARP (Plank et al., 2021) only suitable for R-D simulations.

##### Baseline parameter setting for electrophysiological simulations.

The dimensionality of the parameter space governing the spatial variation in conduction velocity is reduced as follows. First, the overall fiber arrangement is kept constant, and target conduction velocities are prescribed in the TC, PMs, IC, FO, and the wider LA and RA walls, on a per-region basis using reported ranges (D.d. et al., 2012), eventually scaled to ascertain that the total atrial activation times computed with the R-E model fall within the physiological ranges (Lemery et al., 2007).

Anisotropy in conduction velocity between longitudinal and transverse to the fiber directions was set to a value of 1.3 within the reported range of 1.0 to 1.6 (Gray et al., 1996; Hansson et al., 1998). Atrial cellular dynamics were represented by the Courtemanche model (Courtemanche et al., 1998), parameterized for various EP regions following Azzolin et al. (2023) with increased sodium channel conductivity to obtain the prescribed conduction velocities. To identify the electrical conductivities for the R-D model at the targeted mesh resolution that match the R-E conduction velocities, the ForCEPSS framework (Gsell et al., 2024) was employed, using reported values (Roberts et al., 1979) for initialization. The chosen parameter settings are summarized in Table 4. Conduction velocities in the IC cables were considered parameters, tuned to obtain physiological inter-atrial activation delays and, thus, the separation of the contributions of RA and LA to the P-wave.

All simulations were conducted for a duration of 150ms to cover the total atrial activation time and, thus, the entire duration of the P-wave. All ECGs were filtered with a 150.00Hz low-pass and a 0.50Hz high-pass, and scaled by a factor of 0.2 to obtain ECG signals with the observed magnitudes (Gillette et al., 2022).

We assessed the accuracy of the R-E model relative to a gold standard R-D monodomain model for simulating atrial activation and associated P-waves. Using the baseline setup (see Table 4), a normal sinus activation with associated P-wave was simulated on two models of the same biatrial geometry discretized at different spatial resolutions of 0.90mm and 0.25mm for R-E and R-D, respectively, according to their specific numerical requirements (Neic et al., 2017a). A comprehensive description of the EP setup is compactly encoded in a .par file, that is made accessible through the Zenodo platform, doi: 10.5281/zenodo.17143583 to facilitate the seamless reproduction of R-D simulations with openCARP (Plank et al., 2021).

##### Effect of the BB insertion in the LA on the P-wave.

Activation of the LA is mediated through discrete ICs with BB being a primary pathway (Sakamoto et al., 2005; Knol et al., 2019). A site of insertion, x, of an IC defines an earliest activation site, ε(x,t), of the LA and, thus, constitutes a major factor in shaping the LA contribution to the P-wave morphology. With traditional modeling approaches, relying on discrete meshing of the ICs, elucidating such effects is challenging, as any change in the insertion site requires major remeshing of the model. In this work, we aim at investigate the impact of the location of the central BB insertion on the LA activation and P-wave, by sweeping over the spatial parameter $x_{BB_{LA}}$ representing the BB insertion point on the LA wall, that is $\varepsilon \left(x_{BB_{LA}},t_{la}\right)$. The temporal parameter $t_{la}=l_{bb}/v_{bb}$ represents the activation time of ε, and depends on the length of the cable $l_{bb}$, and the designated conduction velocity $v_{bb}$. Here, we chose to prescribed $v_{bb}$, while varying $l_{bb}$ dependently on the insertion point $x_{BB_{LA}}$. Therefore, changes in $x_{BB_{LA}}$ will impact both the LA activation pattern, that is, the P-wave shape, and the initial activation time of the LA, corresponding to a shift in the portion of the P-wave related to the LA activation. The importance of these parameters is investigated concerning the sensitivity of the P-wave shape, and their ability to yield a large enough physiological envelope of P-waves to cover the true measured P-wave.

However, using a single IC cable to mediate BB conduction limits the model ability to produce single point-like LA activation sites, and potentially the ECG shape generation. As the number of inter-atrial cables is not limited in our modeling approach, we investigate a more realistic volumetric-like approximation of the BB band, by using multiple cables. Specifically, we select three points, $x_{BB_{LA}}^{i}(i=1,2,3)$, on the anterior wall of the LA as locations for anchoring three reference cables representing strands of the BB, all of which originate from the midpoint of the RA lateral band of the BB. As per the single-cable case, each location $x_{BB_{LA}}^{i}$ was then varied within a circular area of ≈ 5.00mm, defining a set of alternative entry points $x_{BB_{LA_{j}}}^{i}j=1^{Ni}$, and corresponding automatically generated cables. By running a R-E simulation for each tuple of cables ${\left(x_{BB_{LA_{j}}}^{1},x_{BB_{LA_{k}}}^{2},x_{BB_{LA_{\ell }}}^{3}\right)}_{j=1,\ldots ,N_{1},k=1,\ldots ,N_{2},\ell =1,\ldots ,N_{3},\;}$, the set of P-waves was finally computed.

##### Effect of the discrete RA endocardium on the P-wave.

The endocardium of the RA consists of discrete structures in the form of muscular strands comprising TC, BB and PMs, that are partially attached to a thin and smooth epicardial layer (Corradi et al., 2011; Ho and Sánchez-Quintana, 2009).

The effect of the added endocardial tissue in atrial EP simulation, especially the inter-pectinate tissue, on the P-wave has never been study before. To investigate the role of the RA endocardium on the P-wave, R-E model simulations were run using two different parameter settings for each biatrial model. In one setting, the RA endocardium was considered cardiac conductive tissue, while in the other, the tissue labeled as RA endocardium was treated as part of the bath surrounding the heart. In the former case, conduction velocity tensors, as presented in Table 8, were assigned to the RA endocardium tissue. In the latter case, this tissue was marked as torso tissue.

##### Effect of varying the SAN position and conduction velocities for calibrating P-wave.

We investigate the feasibility of calibrating the atrial activation sequence to the corresponding patient P-wave by sampling key parameters to generate a P-wave envelope covering the observed P-wave data. Due to the high dimensionality of the parameter space, we restrict sampling to three key parameters that are expected to be the most influential in altering the different phases of the P-wave morphology (Loewe et al., 2015). These are:

- the location of the leading pacemaker site within the SAN, responsible for the initial RA activation. This site is known to be variable, allowing modulation of the onset phase of the P-wave. The SAN is an elongated and highly heterogeneous RA region with action potential properties, cell size, and capacity as well as conductance changing from the center to periphery (Honjo et al., 1996), and along inferior-superior and caudal-cranial gradients (Muñoz et al., 2011). For calibration purposes, we avoided modeling the physiological function of the SAN with biophysical detail and instead used only the spatial location of the exit site $x_{\mathrm{SAN}}$ as a parameter. Specifically, the SAN was modeled as a focal activation site, with a short, cigarette-shaped band of 2.20cm in length (Monfredi et al., 2010; Boyett et al., 2000), located in the inferior-central part of the TC. Moreover, the SAN was electrically isolated by a non-conductive region along the boundary of the TC, replicating the effective block observed in physiological studies (Dobrzynski et al., 2005).
- Calibration to the overall duration of earlier phase of the P-wave was based on varying conduction velocities globally within the RA. Velocity in the longitudinal direction $v_{l}$ was uniformly sampled in steps of 0.05m/s between 0.60 and 1.1m/s. Assuming rotational isotropy, velocity in the transverse direction $v_{t}$ was sampled in steps of 0.04m/s between 0.45 and 0.7875m/s, but kept smaller to the longitudinal velocity $v_{l}$, in accordance with D.d. et al. (2012).
- Calibration of the shape and duration of the late phase of the P-wave, was based on manual variation of the anchoring sites of the ICs and of the global conduction velocities in the LA. Specifically, the BB was modeled as a three-cable band initiating at the same origin in the RA, and anchoring in the LA at three independent insertion sites. The other three ICs were also varied in the anchoring position on the LA, and conduction velocities in the cables were manually calibrated to control the timing of the initial LA activation.

##### Calibration of the atrial activation sequence based on the P-wave of the 12 lead ECG.

As a proof-of-concept we demonstrate the feasibility of using our framework for calibrating both global and space-varying EP parameters to match clinical P-waves in four atrial anatomical models randomly selected from our cohort. Specifically, we aimed to identify a subject-specific parameter vector $\omega_{\mathrm{opt}}$ capable of matching the P-wave morphology for each of the four subjects with high fidelity. We employed a Saltelli sampling approach, as described in Gillette et al. (2021b), a quasi-Monte Carlo method designed to uniformly sample the parameter space across the defined variation intervals (Kucherenko et al., 2015). The chosen parameters were varied within physiological bounds for healthy subjects, following the previous studies presented in this work. Specifically, $\omega_{\mathrm{opt}}$ comprised:

$$\begin{array}{l} \omega_{\mathrm{opt}}=({CV}_{\mathrm{ratio}},{CV}_{TC},{CV}_{PM},\\ {CV}_{BB},{CV}_{RA},{CV}_{LA},\alpha_{SAN}^{i},\beta_{SAN}^{i},\\ t_{SAN}^{i},\alpha_{BB}^{j},\beta_{BB}^{j},t_{BB}^{j},\alpha_{CS},\beta_{CS},t_{CS},\alpha_{PC},\beta_{PC},t_{PC}), \end{array}$$

with i=1,2 and j=1,2. Here ${CV}_{\mathrm{ratio}}$ is the ratio between the intrinsic conduction velocities in longitudinal and transverse directions, which is kept constant throughout all anatomical regions, and ${CV}_{\xi }$ is the conduction velocity in the longitudinal to fiber direction for a specific anatomical region ξ=TC,PM,BB,RA,LA. Three possible locations of SAN exit sites are considered on the posterior wall of the RA, together with two BB locations on the front of the LA, and two connections on the posterior wall of the LA, one corresponding to the CS, and one posterior connection (PC) in between the RPVs. All initial activation sites were assumed to be located at the epicardium, and defined based on their UACs. For each of them, the activation time t was also optimized. A summary of the bounds for all 24 parameters comprising $\omega_{\mathrm{opt}}$ is reported in Table 5.

All other EP parameters possibly influencing atrial activation were kept constant. Assuming 500 samples per parameter, a total of 12,000 simulations were conducted. All reference and simulated ECGs were low-pass filtered at 60 Hz. Following Gillette et al. (2021b), the best match was selected as the run achieving the smallest $\ell_{2}$ norm as defined in Eq. (2).

##### Data analysis.

We performed both qualitative and quantitative analyses of EP and P-wave variations resulting from the previously described modeling assumptions. The overall tissue activation was qualitatively analyzed by recording the total activation time of both atria. Changes in P-waves were quantitatively investigated using three different metrics.

In the presence of a reference signal $\varphi_{r}^{j}$, either simulated or given as clinical ECG data, we analyzed amplitude variation with the RMSE in percentage, as described in Keller et al. (2010), Zappon et al. (2024). For each lead j, the RMSE is expressed as:

$$RMSE\left(\varphi_{v}^{j}\right)[\%]=\sqrt{\frac{\sum_{n=1}^{N}\;{\left(\phi_{v}^{j}(n)-\varphi_{r}^{j}(n)\right)}^{2}}{\sum_{n=1}^{N}\;{\left(\varphi_{r}^{j}(n)\right)}^{2}}}\cdot 100.$$

We computed the RMSE when comparing the R-E and R-D models when exploring the effect of the variation of the SAN and RA conduction velocity on the P-wave.

When analyzing the variations of the BB, whether using one or three cables, none of the computed ECG signals matched the real ECG data and could not be used as a reference to calculate the RMSE. For these test cases, we first defined an average P-wave, ${\overset{‾}{\varphi }}^{j}$, for each lead j. We then evaluated the overall morphological and amplitude P-wave variation by computing the mean absolute distance (MAD) of each resulting P-wave $\varphi_{v}^{j}$ from the average one as:

(1) $$MAD\left(\varphi_{v}^{j}\right)=\frac{\sum_{t=1}^{N}\;\left|\varphi_{v}^{j}(t)-{\overset{‾}{\varphi }}^{j}(t)\right|}{N},$$

where $\varphi_{v}^{j}(t)$ and ${\overset{‾}{\varphi }}^{j}(t)$ are the varying computed signal and the average signal at time t, and N is the total number of ECG time samples. The MAD is then averaged over all leads.

Additionally, in all cases, we accounted for the variation of PWD, obtained using a sloping approach (Tan et al., 2000). The PWD was measured for each lead. Moreover, the PWD averaged over all leads was given.

For matching computed and measured P-wave employing the Saltelli sampling approach, a $\ell_{2}$ norm was adopted to define the best fit (Gillette et al., 2021b), computed as the average of the $\ell_{2}^{j}$ norm of each lead j:

(2) $$\ell_{2}^{j}\left(\varphi_{v}^{j}\right)=\frac{\sqrt{\sum_{n=1}^{N}\;{\left(\varphi_{v}^{j}-\varphi_{r}^{j}\right)}^{2}}}{N}.$$

## 3. Results

### 3.1. Model generation workflow performance evaluation

We evaluated our atrial model generation workflow with respect to the required processing time and the achieved degree of automation. 50 contrast CT datasets of AF patients were processed to generate biatrial models of target resolution of ≈ 0.25mm and ≈ 0.90mm, and the execution times of individual stages of the automated workflow were measured. Part of the generated models will be available on the Zenodo platform, doi: 10.5281/zenodo.17143583.

After each stage, visual checks were performed to detect processing errors. Errors that required manual correction were recorded for each stage and at both target resolutions, and are summarized in Table 6. Benchmark results are reported for the execution of the workflow on a compute workstation equipped with an AMD Ryzen Threadripper PRO5965wx processor, using 16 CPU cores.

**Fig. 9.**
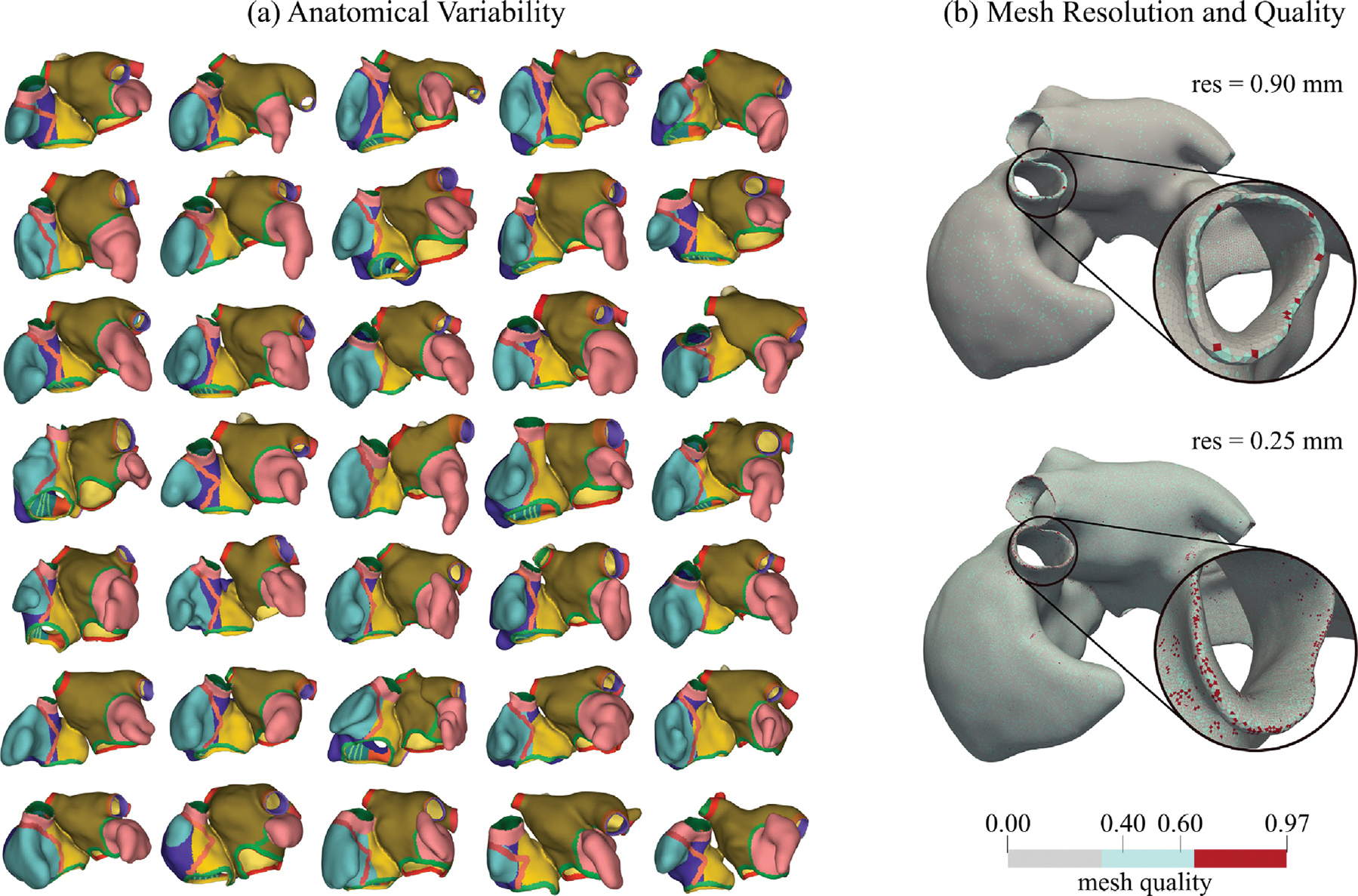
(a) Representation of 35 out of 50 generated biatrial models. Only a subset of the generated geometries is represented to allow for a better visualization. (b) Examples of computed meshes of resolution of ≈ 0.90mm and ≈ 0.25mm and corresponding obtained mesh quality.

Overall, all 50 cases in both resolutions were processed automatically in the majority of cases (38), with only minimal user intervention required in 22 cases. The workflow produced anatomically highly detailed computational meshes, with fine-grained domain annotation, fiber arrangement, and an anatomical reference frame in the form of UACs (referred to Fig. 9). For the lower resolution R-E model, the overall processing per model lasted, on average, less than ten minutes. Higher resolution R-D compatible meshes were more costly to generate, specifically, the UAC generation stage where more than ≈84% of the costs incurred. A comparison with existing UAC software and computations from Roney et al. (2023) was conducted to evaluate the effectiveness of our framework in terms of UAC quality distribution, usability, and computational cost. The results are provided in Appendix. Importantly, a fully automatic meshing processes was achieved in all cases, yielding meshes free of topological errors and of, overall, excellent mesh quality. Worst element quality was always below 0.99, according to the quality metric (Karabelas et al., 2018), which is considered a critical threshold in simulations using R-D solvers such as openCARP. Lower quality elements close to 0.99 clustered mostly in high curvature regions, around the orifices of the thin-walled atria (referred to Fig. 9).

#### 3.1.1. Automation failure

A manual intervention was needed to separate the LSPV from the LAA in 19 cases, and the CS from the LA in 2 cases. The incorrect segmentation was however not due to the SCN, but to the low contrast in the acquired images. At the second stage, during the labeling of the blood pools, manual intervention was required to switch the CS with the IVC landmarks in 21 cases. The final marking of the veins and the corresponding tissue to discard from the blood pool, including the CS, LAA, and RAA, was automatically done by the workflow in 28 cases. For the remaining 22 cases, manual intervention was only needed to define or adjust the tissue to discard from the IVC to ensure a better opening.

### 3.2. EP simulation workflow performance evaluation

**Fig. 10.**
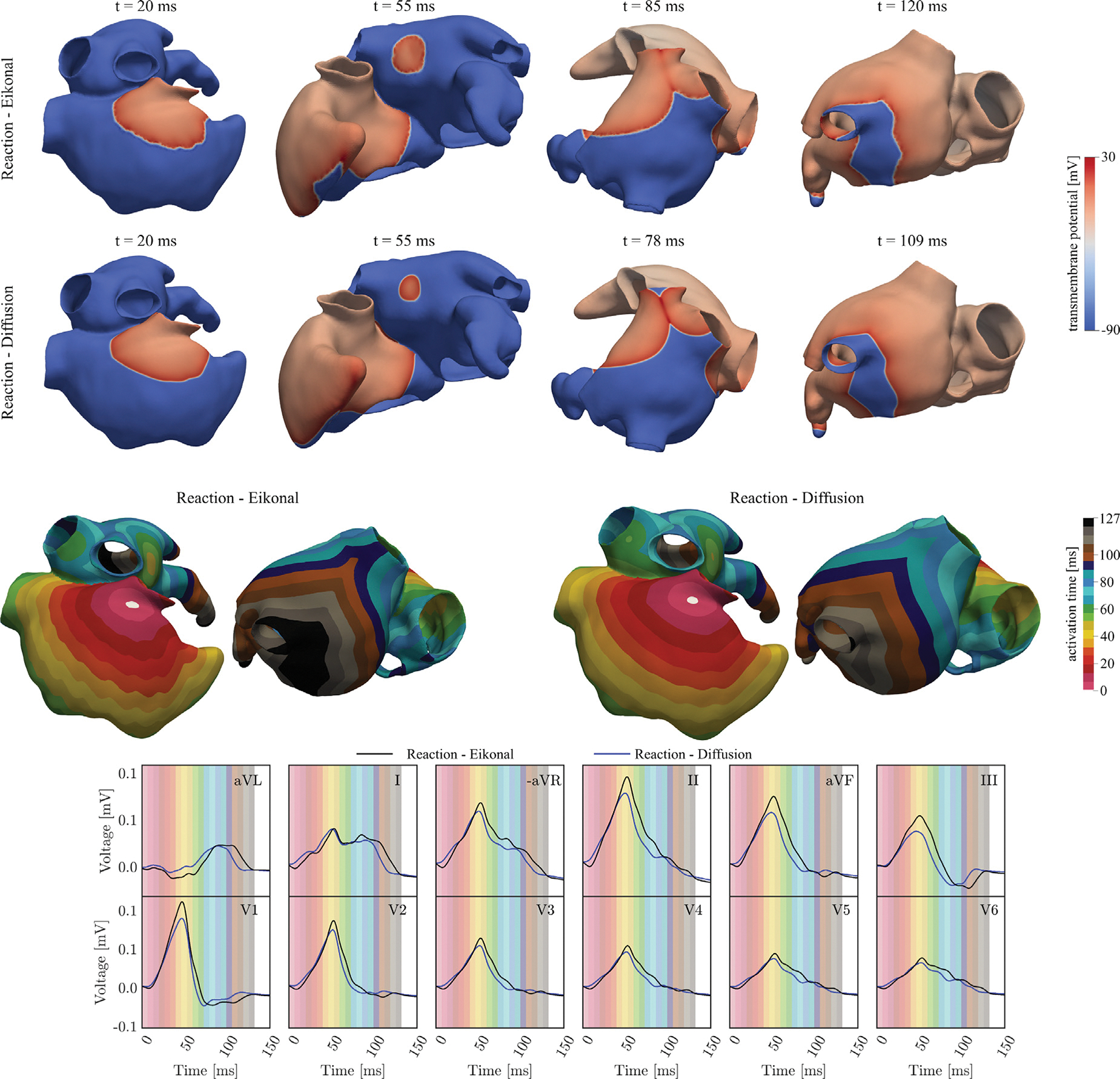
First/Second row: Transmembrane potential $V_{m}$ propagation computed with the R-E and R-D models, at different time instants. Third row: Activation map obtained by solving the R-E and the R-D model. Fourth row: ECGs obtained by solving the R-E (black) and R-D (blue) models coupled with the lead field. The background colors refer to the activation map. By coloring the background with time-bands of colors corresponding to the activation map, the activation map is compared with the ECG, highlighting the relation between regional atrial activation and P-wave.

The efficiency of our workflow in the function twinning stage, that is of simulating atrial EP for high fidelity ECG generation, was tested for both R-E and R-D models for all 50 patients. Execution times of individual processing stages – comprising torso generation, setting up of ICs, computation of the ECG lead fields, and the simulation of an entire atrial activation sequence initiated at the SAN – were measured (refer to Table 7 and Fig. 10). As shown previously in detail (Gillette et al., 2021b), owing to its relaxed mesh resolution dependency, the R-E model is significantly more lightweight, facilitating the setup of an EP model in ≈ 3.00min and the computation of a full biatrial activation sequence with a high fidelity ECG in ≈ 1.00s. All steps of the workflow are computationally more costly due to the stricter mesh resolution requirements, requiring ≈ 32min for setup, and ≈ 19min for computing the activation sequence and the ECGs. While formally the same torso surface was employed, extra costs were incurred due to the required mesh conformity at the atrial surfaces, leading to a markedly higher number of elements in the R-D case.

The computational costs of creating the IC cables also showed slight differences between the coarse 0.9mm mesh and the fine 0.25mm mesh, averaging 19s and 39s, respectively. However, this difference is negligible compared to the total costs of generating the biatrial and torso geometry, as well as the CPU time required for the computation of the lead field solution, which is of 35s for the R-E mesh, and 9min for the R-D grid, and the EP simulation, corresponding to 27s of computation for the R-E model, and to 19min for the R-D gold-standard monodomain model.

### 3.3. Assessing discrepancy between R-E and R-D model

To evaluate the computational fidelity of the R-E model compared to a gold standard R-D monodomain model (Nagel et al., 2022), we compare the spatiotemporal distribution of electrical sources, $V_{m}(x,t)$, the activation maps, τ(x), and the P-wave generated by both models, employing the baseline parameter settings in Section 2.2.2. Results are illustrated for a representative test case in Fig. 10.

Overall, the model parameters calibrated to match R-D and R-E conduction velocities led to nearly identical activation patterns. Minor differences emerged due to imperfect matching of conduction velocities in the RA and LA tissue, as well as in the IC cables, and differences in anterograde and retrograde activation by the ICs cables mediated by an electrotonic source–sink mismatch at the interface between cable and tissue (refer to Fig. 10, top panels). These combined effects led to a slightly longer total activation time of the atria, which manifested in minor differences in the PWD, with an average variation of 2ms.

Differences in P-wave morphology and magnitude were negligible, with a maximum amplitude difference in lead II of 5%, and an average RMSE across all leads of 3.71% (refer to Table 8). Magnitude differences could be attributed to the difference in spatial resolution, as these disappeared when running the R-E simulation on the higher resolution mesh (not shown).

### 3.4. Impact of Bachmann’s Bundle insertion on the P-wave

**Fig. 11.**
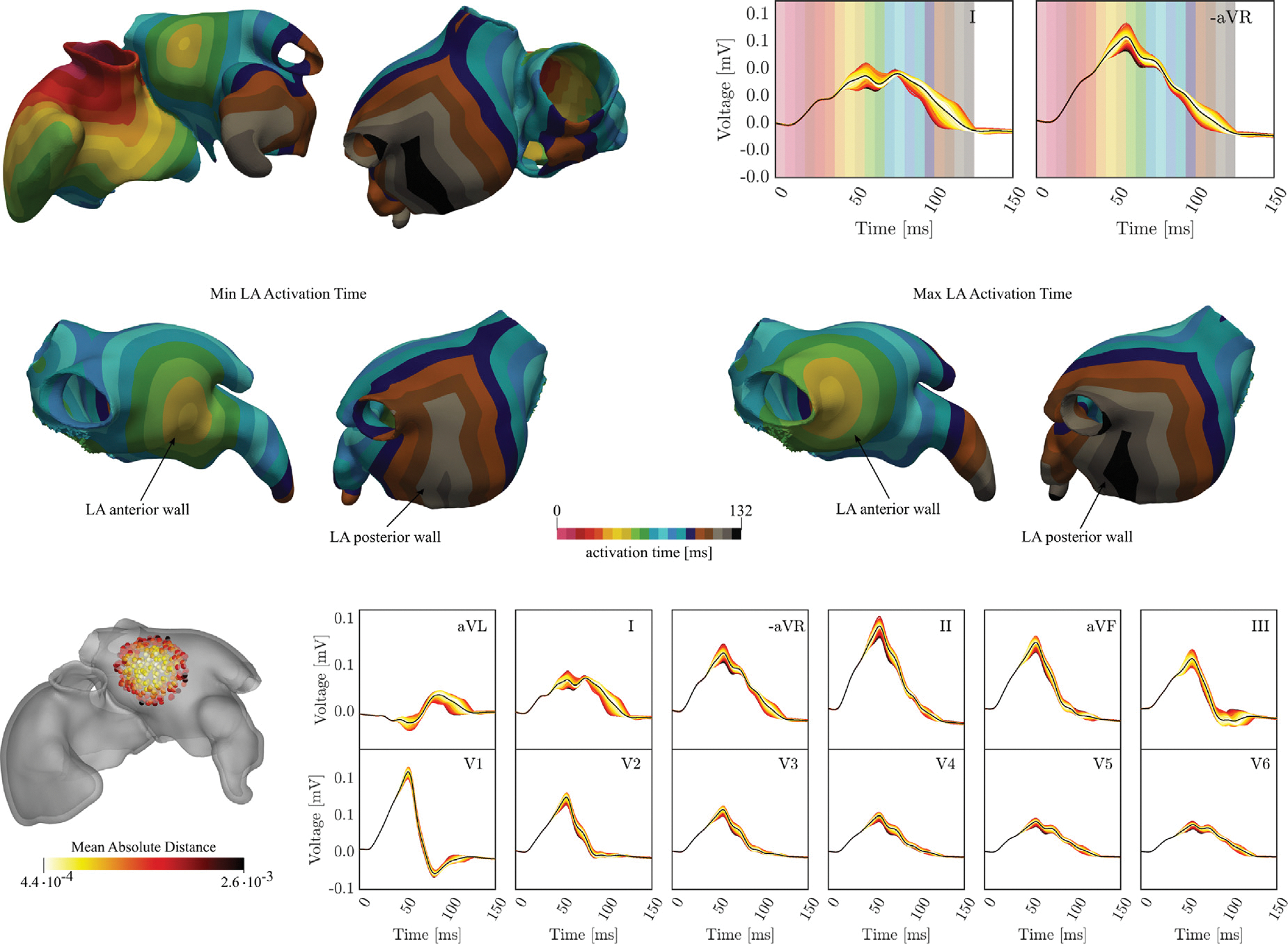
Influence of BB LA insertion site. First row: Biatrial activation map obtained for BB reference insertion site along with P-waves in leads I and -aVR. The background of P-wave traces is color coded according to the activation map to highlight the relation between the dipole layer of depolarization wave fronts and the associated amplitude in the P-wave. Second row: LA activation maps corresponding to the minimum and maximum total activation time. Third row: P-wave variation due to varying the BB insertion site in the LA. Insertion site an ECG are color coded corresponding to the absolute distance from the mean ECG (black), averaged over time and the 12 leads.

The role of BB insertion site $x_{BB_{LA}}$ on the LA activation and P-wave morphology was investigated. The use of a cable-based IC formulation readily facilitated a parametric sweeping of $x_{BB_{LA}}$, where $x_{BB_{LA}}$ was varied within a radius of ≈ 5mm around the reference location, ${\overset{ˆ}{x}}_{BB_{LA}}$, as used above. Shifts in $x_{BB_{LA}}$ altered the location and timing of wavefronts collisions with waves initiated through the other ICs. These were predominantly the rim of the fossa ovalis, activating the LA almost synchronously with the BB, and the posterior-superior IC, activating the carina of the LPVs with a delay of 10ms. This entailed a change in the total activation time of the LA, and induced a spread in PWD and magnitude of the P-wave, with very limited effect on the corresponding morphology (refer to Fig. 11).

Variation of $x_{BB_{LA}}$ alone led to differences in total activation time of 23ms, in PWD between 12.00ms to 24.00ms in individual leads, with a minimum and maximum average PWD of 104 and 120ms across all leads, respectively, and in magnitude to a mean absolute distance (1) between 4.4 × 10^−4^ and 2.6 × 10^−3^ mV. A quantitative summary on PWD variability is given in Table 9.

### 3.5. Effect of a fan-like LA insertion Bachmann’s Bundle on the P-wave

**Fig. 12.**
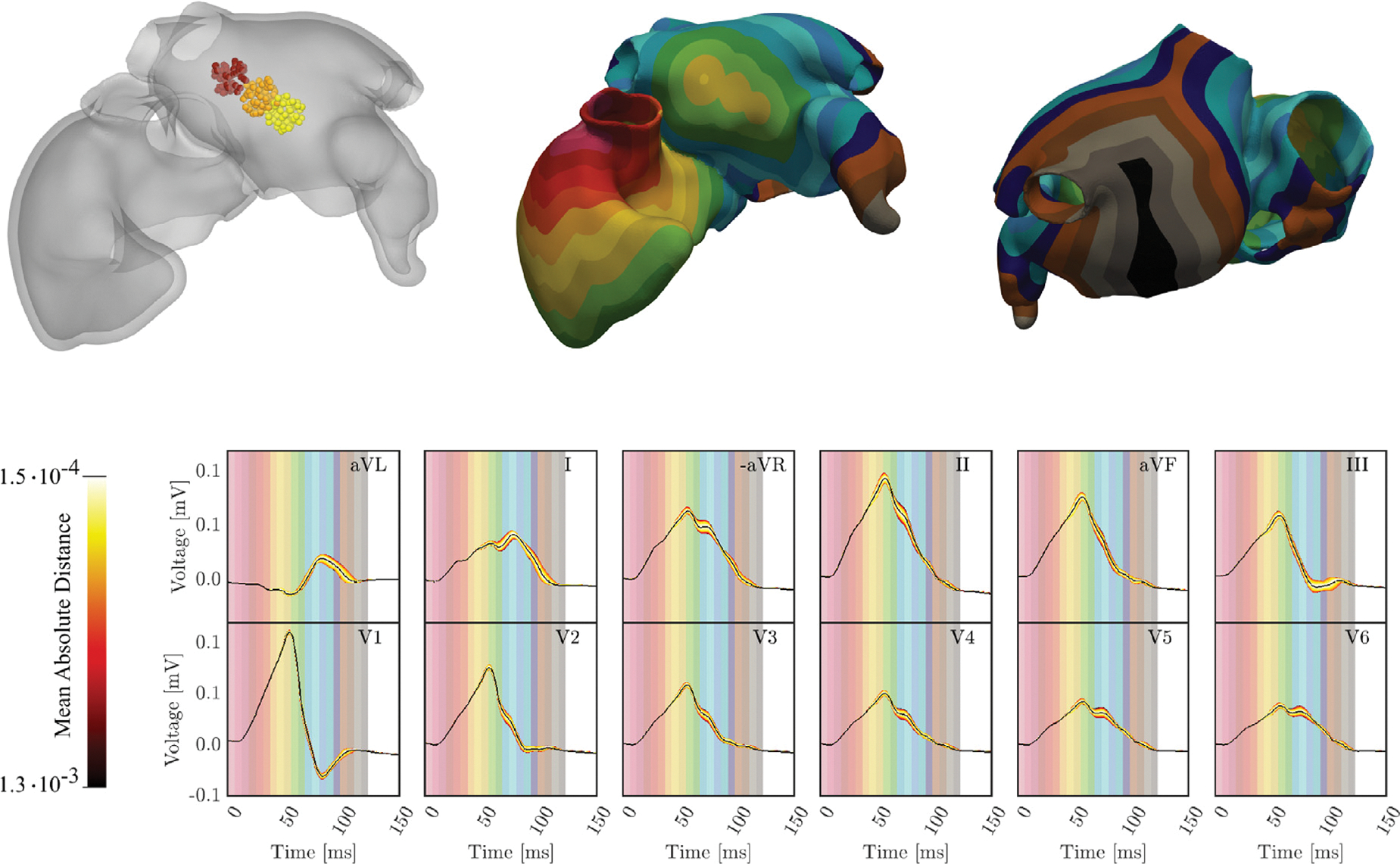
First row-left: Investigated LA entry site areas of the three cables representing the BB. The different colors identify the three different regions of search. First row-center and right: An example of activation map obtained with the three-cable BB. Second row: P-wave variation obtained by moving the LA entry sites of the three cables representing the BB. The signals coloration correspond to the absolute distance from the mean ECG, averaged over time and the leads. The mean ECG is shown in black. By coloring the background of the lead with time-bands of colors corresponding to the activation map, the activation map is compared with the ECG, highlighting the relation between regional atrial activation and P-wave.

Our modeling approach flexibly supports an arbitrary number of ICs cables which can be bundled to increase source strength for generating ECGs and EGMs, or to model inter-atrial coupling with higher anatomical complexity. This ability is showcased for investigating the influence of a distributed fan-like insertion of BB into the LA with three independent coupling locations. An exemplary activation map along with the resulting distribution of P-waves is shown in Fig. 12. As the same velocity was assumed in all cables the initial activation at the three LA insertion sites was not synchronous but led to an elongated area of early activation along the location of the wide BB.

The total activation time was observed between 118 and 125ms, comparable smaller than the BB single-cable case.

The highest variation in the P-wave was observed between 80 and 132ms, corresponding to the activation of the posterior wall of the LA. The average range of variation of the PWD was registered from 102 to 110ms, thus smaller than the single-cable case. The maximum and minimum PWD over the leads was also reduced compared to the single-cable case, with maximum variation in lead aVL and II (see Table 10).

### 3.6. Effect of the RA endocardium on the P-wave

**Fig. 13.**
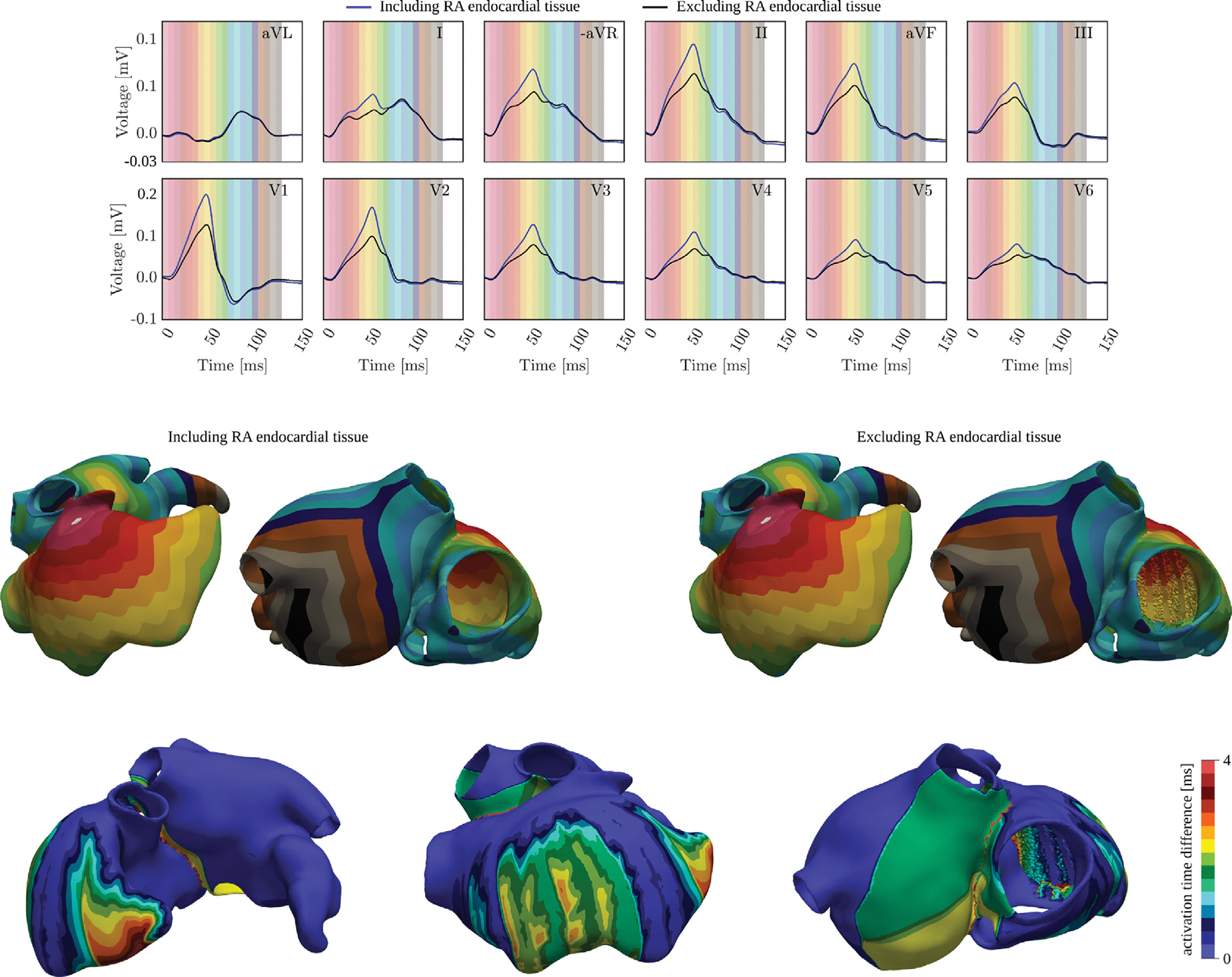
First row: P-wave obtained when including (blue) and excluding (black) the RA endocardial tissue outside of the PMs and TC. The P-wave of all leads is compared to the activation map by coloring the background. Second row: Activation maps obtained when including (left) and excluding (right) the RA endocardial tissue. Third row: Absolute difference between the activation maps obtained when including and excluding the RA endocardial tissue.

The ECGs obtained by simulating the atria, both including and excluding the RAs endocardial layer outside of the PM and TC, are shown in Fig. 13. A decrease in wave amplitude between 14 and 71mV, corresponding to the activation of the RA, was observed in all leads. The RMSE in percentage is reported in Table 11. The major amplitude variation, exceeding 1%, was observed in leads II, V1, and V2, which capture the signal propagation towards the RAA and the PM. Variations between 0.49% and 0.91% were observed in all other leads, except for aVL, where the variation was almost null. The PWD, as reported in Table 11, remained unchanged in all leads.

The ECG variations were related to the computed activation maps and their absolute difference, depicted in Fig. 13. When the RA endocardial wall was included, the signal propagated faster on the posterior part of the RAA, on the posterior-lateral wall of the RA near the PMs, and on the septum. A total activation delay of about 2.1 ms was observed at the tip of the RAA, and 2.7 ms on the epicardium between the PMs. The BB, RA anterior wall, and the TC were activated at the same speed. However, the removal of the RA endocardial layer between the TC and the septum caused a delay in the activation of the septum and the RA wall near the CS. Although this delay did not affect the overall RA activation, it slightly impacted the activation of the posterior wall of the LA, causing a total delay of up to 2 ms in the posterior-inferior region. Nonetheless, the total activation map of the LA remained almost unchanged, consistent with the computed ECGs.

### 3.7. Effect of varying the location of the SAN exit site and conduction velocities for calibrating P-wave

**Fig. 14.**
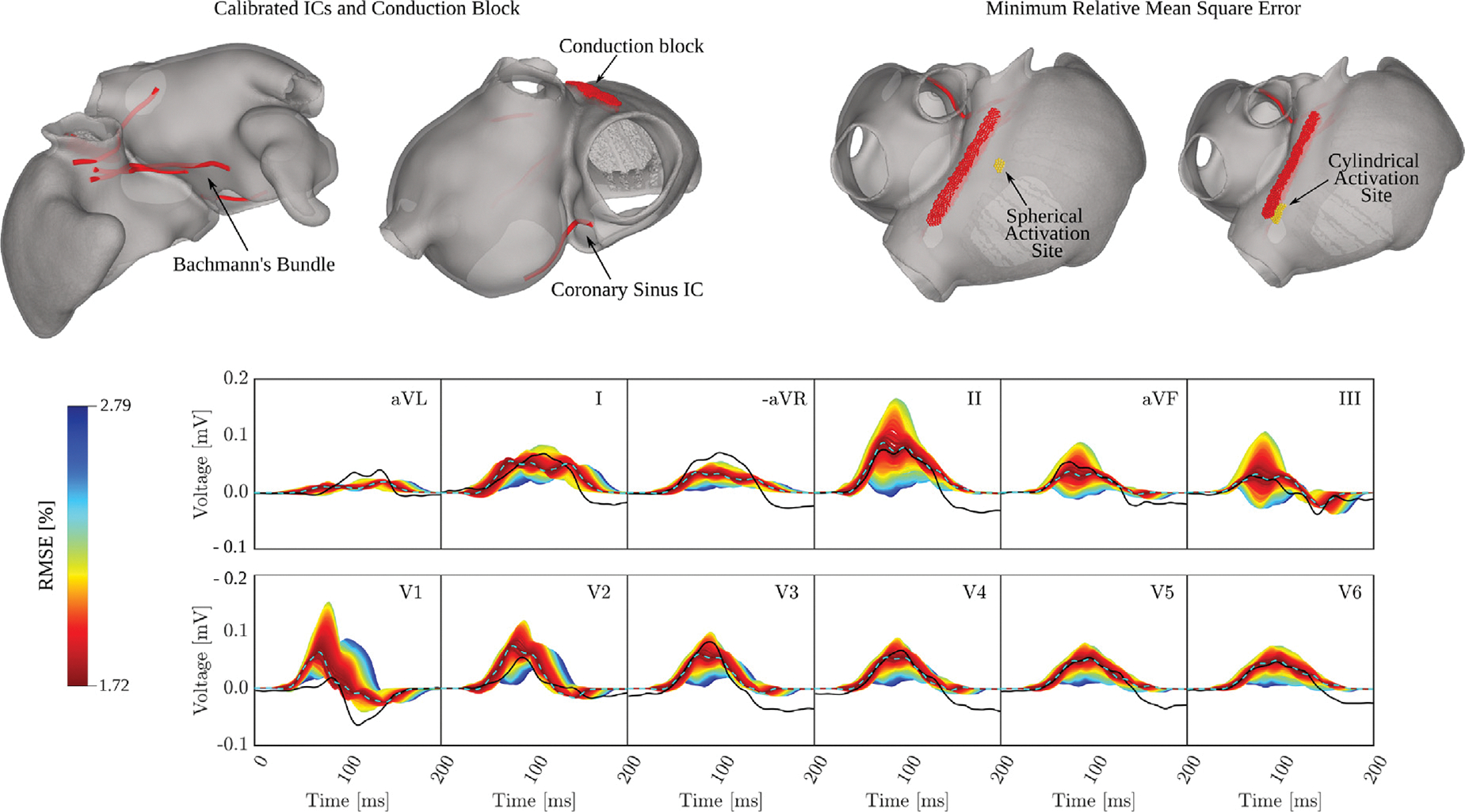
P-wave based calibration of the atrial activation sequence. Top row, left: Constant ICs configuration obtained by interactive pseudo-calibration. Top row - right: Spatial sampling ranges of SAN exit sites with optimal location that minimized the mismatched as measured by the RMSE. Bottom row: Physiological envelope of P-waves (colored traces) obtained by varying SAN exit site, and the velocities $v_{f}$ and $v_{s}$ in the RA together with the clinical P-wave (black line), and the computed P-wave with minimum RMSE (light blue and dashed line). Traces are color-coded according to their distance to the clinical P-wave measured as RMSE.

To keep the calibration procedure tractable, the parameter space to be explored was restricted to the initial exit site at the SAN, the conduction velocity in the RA, and the location and timing of three insertion sites of BB in the LA. In a first pseudo-calibration step for a fixed SAN exit site and baseline velocities in the RA, the location of BB was varied in interactive simulation runs to obtain a close approximation of the terminal half of the P-wave. One configuration yielding a good morphological fit in all leads, assessed only by visual inspection, was selected (see Fig. 14, top row).

Keeping the BB fixed, the physiological envelope of the P-wave signals was computed by sampling over $x_{SAN},\;v_{f}$, and $v_{s}$ (see Fig. 14, mid row). The P-wave envelope under this sampling covered the observed clinical signal in most leads quite well, with the exceptions of leads aVL, -aVR and V1. While the discrepancy in the low amplitude P-waves in leads aVL and -aVR were rather small, this was not the case in lead V1 where the biphasic P-wave could not be approximated. The terminal negative deflection of the measured P-wave was likely mediated by fibrotic tissue in this AF patients with impaired EP excitability and repolarization properties.

Quantitatively, the P-waves were well approximated, with a RMSE in the range of [1.72%,2.79%]. The minimum RMSE was achieved when the SAN was positioned at 62% along the IVC-SVC axis and 35% along the lateral-to-septal axis (see Fig. 14, top row), with conduction velocities of $v_{f}=1.1\;m/s$ and $v_{s}=0.45\;m/s$. The RMSE values, as well as the PWD for each lead, obtained with the optimal parameter set that minimized RMSE are presented in Table 12. On average, simulated PWD was longer than clinical PWD, with 139ms versus 130ms, suggesting that calibration could be further improved by including IC and LA velocities in the sampled parameter space.

### 3.8. Calibration of the atrial activation sequence based on the P-wave of the 12 lead

As proof-of-concept we selected four atrial anatomical models, P1,…,P4, to demonstrate the feasibility of employing our framework for automatically sweeping of both global and space-varying parameters to identify the $\omega_{\mathrm{opt}}$ of optimal EP parameters that minimize the mismatch between predicted and clinical P-wave. The optimal parameters for each patient are reported in Table 13. Activation sites triggering after depolarization by a propagating wave front were removed from $\omega_{\mathrm{opt}}$.

**Fig. 15.**
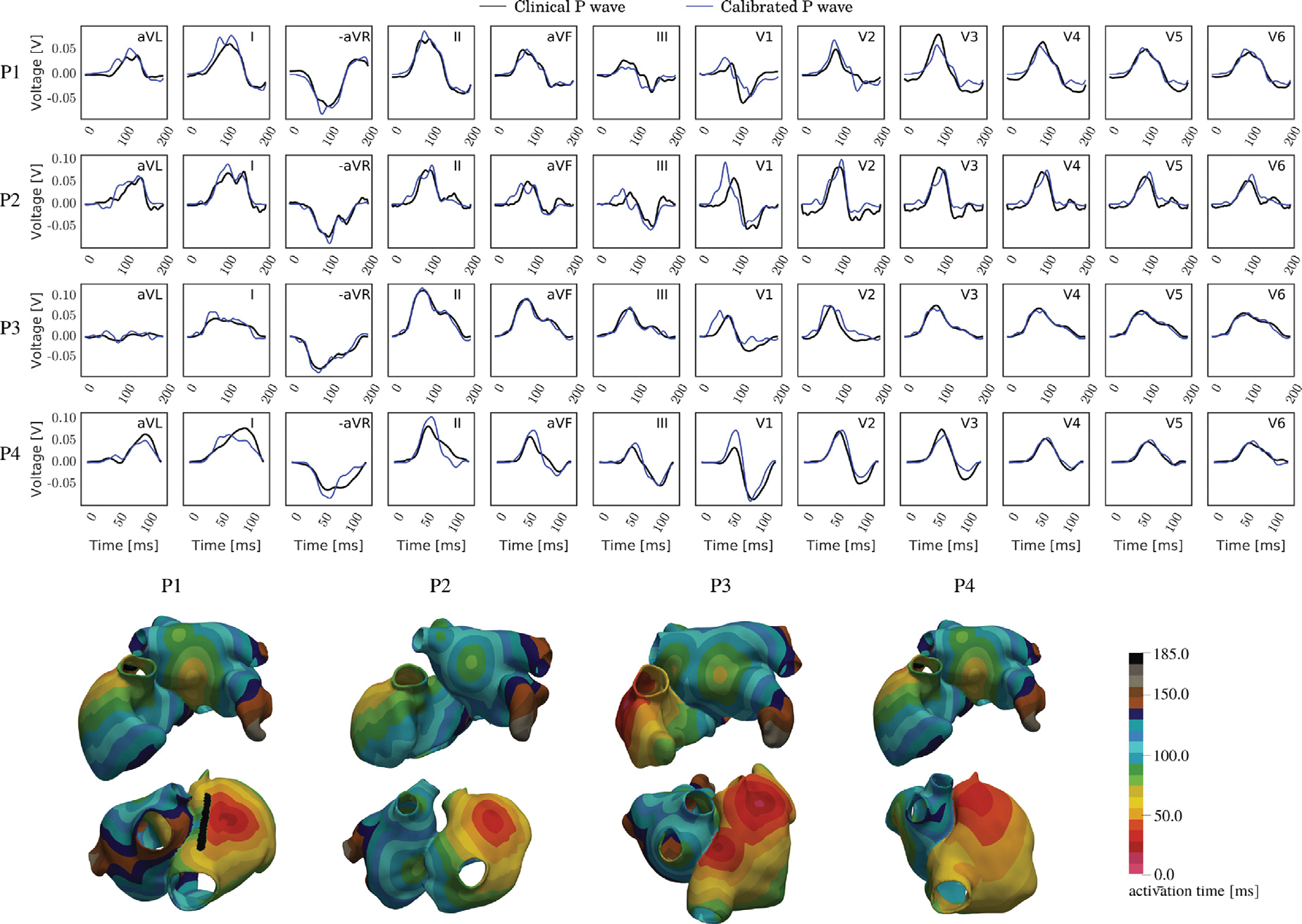
P-wave based calibration of the atrial activation sequence of four atrial anatomies randomly selected between the available in the AF dataset. Top: Qualitative comparison between the clinical P-waves (black) and the simulated ones (blue). Bottom: Atrial activation sequences obtained from the calibration process.

The match between simulated and measured P-waves is qualitatively illustrated in Fig. 15, along with the corresponding activation maps. For all four patients, the simulated P-wave exhibited the correct polarity across all leads, and a close match in signal morphology could be achieved. Unlike with advanced gradient-based optimization methods (Grandits et al., 2024a), achieving a near-zero loss perfect match proved unattainable. Discrepancies emerged predominantly in the leads V1 - V3 closest to the heart, and to a lesser extent in the more remote limb leads.

## 4. Discussion

In this study, we present a highly automated scalable end-to-end workflow for generating patient-specific anatomical digital twins of human atria for their efficient calibration based on clinical data, e.g. the ECGs. Our novel workflow is comprehensive, including the generation of an anatomical reference frame, that facilitates parametric encoding of all space-varying EP model properties, as required for model calibration, a detailed annotation of all relevant anatomical landmarks and structures, including a fiber architecture based on a detailed anatomy-informed set of rules, and a new method for lightweight and flexible ICs.

Our workflow, implemented in a single software building on meshtool (Neic et al., 2020), generates smooth biatrial anatomical representations of sufficient mesh quality for a range of resolutions suitable for the entire spectrum of atrial EP simulations. Both coarser meshing at ≈ 0.90mm for real-time calibration using R-E type models (Neic et al., 2017b; Pezzuto et al., 2017), as well as finer meshing with sufficient resolution for fully mechanistic R-D modeling studies, is supported. The workflow efficiently generates simulation-ready models within less than 10 min at a reference resolution of approximately 0.90mm. In contrast to previous approaches for building volumetric biatrial models, that used statistical shape models to generate virtual anatomies (Roney et al., 2023; Nagel et al., 2021), our approach generates accurate representations of an individual patient’s atrial anatomy, with fidelity being limited only by image quality and segmentation accuracy. A key advantage of our approach is its robustness, as the description of anatomy is built on a volumetric image stack. This avoids the more common manifold extrusion procedure, prone to topological mesh errors. A major limitation hindering a full automation of the procedure is the identification of the IVC, which required manual correction in almost 40% of the cases. However, the manual correction is straightforward to implement, requiring only a few seconds.

Building on this anatomical model generation pipeline, the workflow is moreover extended for ECG based model calibration, employing either R-E-based real-time EP modeling, suitable for EP calibration, or a full fidelity computationally more demanding R-D model, suitable for predictive simulations. Inter-atrial conduction is modeled by initially imposing electrical insulation between the RA and LA (Costa et al., 2014), and then discretely reconnecting them using a novel, highly flexible, and physiologically constrained method. This approach employs auto-generated cables to establish inter-atrial conduction pathways, accurately representing all anatomically significant ICs between the RA and LA. Our approach facilitate the generation of pathways with prescribed conduction velocities to connect arbitrary parametrically steerable locations within RA and LA on the fly, and, thus, avoids rigid and error-prone explicit meshing of inter-atrial bundles (Nagel et al., 2021).

Finally, we employ a forward ECG generation framework based on the reaction-eikonal lead field (RELF) model that yields full fidelity ECGs with real-time performance, to support a clinically compatible model calibration procedure. To this end, volumetric atrial models are swiftly registered with reference torso models with corresponding electrodes, and integrated into volumetric conformal atria-torso meshes. All combinations of cardiac EP source and field models are supported. As mesh conformity is preserved, high fidelity ECGs and EGMs can be computed over the entire solution domain based on either bidomain or lead field formulation, without being restricted to lower fidelity methods such as potential recovery (Bishop and Plank, 2011a), or boundary element approaches, that require smooth low pass pass-filtered coarse representations of the atrial sources (Schuler et al., 2019).

The ability to compute realistic ECGs across different modeling scenario with close to real-time performance is demonstrated by exploring important parameters governing the genesis of the P-wave, which were impossible or notoriously difficult to explore with previous approaches, e.g., the anatomy of ICs, and by calibrating sinus rhythm P-wave of four atrial anatomical models with a forward sampling approach. We show the equivalence in activation patterns and ECGs between the lower resolution R-E model, and the high resolution R-D model, with discrepancies well below the overall model uncertainty. These combined features make our end-to-end workflow suitable for large-scale atrial EP modeling studies. The achieved model efficiency supports a fast exploration of the high-dimensional parameter space spanned by atrial anatomy, structure, and EP, by facilitating unattended sweeps over important space-varying parameters, which is needed for the automation of optimization loops using ECG or EGM observations as target for calibration. While these numerical tests are not intended to demonstrate a perfectly calibrated atrial model, they highlight the flexibility of our modeling framework in easily exploring a wide range of scenarios related to atrial EP and P-wave generation, a task that was previously hindered in existing works by limited anatomical model flexibility and the high computational costs associated with EP modeling choices.

### 4.1. Scalable generation of volumetric biatrial anatomy models

Robust computational workflows capable of generating comprehensive volumetric biatrial anatomical models, with fiber architecture and anatomical reference frames at scale remain challenging. Only a few approaches have been reported to date, exhibiting significant variation in the degree of automation, anatomical and structural fidelity, flexibility in prescribing fiber architecture, and their ability to generate clinically observable signals such as EGMs and ECG with sufficient accuracy.

The majority of atrial modeling studies using larger cohorts relied upon a bilayer formulation (Labarthe et al., 2014) which greatly simplifies the meshing procedure and reduces computational expenses at the cost of decreased biophysical fidelity. For instance, it has been demonstrated that the behavior of bilayer models may differ strikingly from full 3D models with respect to propagation patterns and arrhythmia dynamics (Roney et al., 2021). Discrepancies may stem from various factors such as e.g. altered source–sink relations, the impact of mediating transmural conduction through resistive coupling between the endocardial and epicardial layers using finite elements of incompatible dimensionality, or differences in inter-atrial conduction pathways. Another important limitation of bilayer models is their reduced accuracy in representing the atrial sources, and a lack of suitable approaches for computing higher-fidelity extracellular potential fields (Bishop and Plank, 2011a), due to incompatibility with standard Finite Element Method (FEM). As such, modeling clinical data, such as the EGMs or ECG, with high fidelity is challenging. Moreover, modeling extracellular potential field-related physiological events, such as bath loading effects, cannot be represented at all (Bishop and Plank, 2011b). These factors combined limit their trustworthiness and application scope.

Volumetric biatrial meshes generated in early pioneering studies used artisanal handcrafted non-scalable methods that were tractable only for constructing small-size cohorts, often consisting of a single model only (David M. Harrild, 2000; Seemann et al., 2006). Later, model-generation pipelines were proposed, gradually refined, and increasingly automated towards a more industrialized process for producing models *en masse*. A first mostly complete workflow was reported in a study by Krueger et al. (2012) which was applied to generate a cohort of nine biatrial models. However, as numerous manual interventions were required in segmentation and landmark selection, for fiber mapping, and anatomical region definition, scalability remained limited.

A further automated approach using a segmentation-derived endocardial surface mesh as input was reported and tested in 29 patients in Azzolin et al. (2023). The approach bears similarities to ours in that e.g. surface curvature measures are used to identify structures such as the pulmonary veins, but also differs markedly in other processing steps which are more challenging to perform when using manifold meshes only. For instance, the identification of the mitral valve orifice, which is trivially identified as a label interface in our volumetric approach, required a rigid registration with a mean statistical shape model. Moreover, important anatomical landmarks such as the FO and its rim or the CS were not identified, and additional manual procedures were required to identify anatomical regions such as the LAA.

The largest atrial modeling cohort study comprising 1000 biatrial models has been reported in Roney et al. (2023). While this is indicative of robustness and scalability, the numerous interactive manual interventions required suggest a labor intense procedure. Moreover, the workflow was primarily tailored for generating patient-specific bilayer models, as only these were derived from clinical imaging data. Support for generating volumetric models was limited to volumetric remeshing of pre-existing manifold meshes built from a statistical shape model (Rodero et al., 2021), and auxiliary data such as fiber architecture and UACs were transferred over from an atlas. A direct generation of anatomically accurate patient-specific 3D models from images, as per our approach, is currently not supported. Similarly, in the study by Nagel et al. (2021), volumetric biatrial models were also generated using statistical shape model manifolds as input to a heterogeneous workflow that integrated five different mesh generation and manipulation tools. Furthermore, in Roney et al. (2023), the considered volumetric models did not include veins ostia, which were cutted to leave a corresponding hole on the atrial body. However, the atrial veins and ostia are critical component of the model as ectopic triggers tend to be located there, and electro-anatomical mapping also often extends into the sleeves of the veins. Our approach offers a tight control over the length of the muscular sleeves within the orifices of the veins. This is critical to include with regard to modeling P-waves associated with these triggers. Also the muscular width of the sleeves in 3D is an important factor to consider as the formation of ablation lesions to isolate the veins will depend on this.

### 4.2. Performance considerations

The scalability of model generation methods depends on two major factors, the degree of automation to limit time-consuming interactive processing, and the robustness and speed of the processing steps, from medical image to final mesh. Our workflow achieves the highest degree of automation reported so far, with minimal to no interactive processing at all. Using a SCN (Thaler et al., 2021) all CT data sets were automatically segmented within <10.00 s. As all required labels were detected robustly and accurately from all contrast CT datasets used in this study, with no need for manual label correction. The additional landmarking of veins and appendages can be fully automated, requiring minimal user input to verify or, if necessary, correct the auto-generated labels. This process includes an interactive step supported by a custom tool designed for quick label correction, reducing the overall operation time to less than 1.00 min. For all subsequent steps, including the generation of the volumetric atrial walls, the assignment of a rule-based fiber architecture and the computation of UACs, full automation has been achieved. Performance measured over 50 models yielded execution times of only <10.00 min and <30.00 min for generating an atrial model at mesh resolutions suitable for R-E and R-D simulations, respectively. Furthermore, although the cohort used in this study included contrast CT scans only, the SCN has been previously tested and validated with other imaging modalities, including MRI, LGE-MRI as well as most recent photon counting detector CT (Thaler et al., 2021). As such, these imaging modalities are also suitable inputs for our workflow.

### 4.3. Mesh quality

A common limitation of most previous approaches is the mesh quality of the generated models. To evaluate this, we randomly sampled publicly available datasets of biatrial anatomies and found degenerated elements in all the sampled meshes (Roney et al., 2023; Nagel et al., 2021). These deficiencies went unnoticed as all studies employed R-D monodomain models which cope well with poor mesh quality. However, employing these meshes for higher fidelity bidomain-based EGM generation is often challenging, since it requires the solution of sensitive elliptic problems, whose solution may be associated with slow convergence or even solver divergence in the presence of degenerated elements when employing classical solvers (Conley et al., 2016; Schneider et al., 2018). Moreover, for the sake of versatility, workflows for generating anatomical models of the human atria should be ideally developed with a broader range of potential applications beyond electrophysiology in mind, such as simulations of atrial mechanics and blood flow, where mesh quality has been shown to play a critical role (Karabelas et al., 2018). Mesh quality issues and topological errors may arise from volumetric mesh construction methods that depend on the direct extrusion of the atrial walls from the mesh manifolds, Azzolin et al. (2023), Nagel et al. (2021). In contrast, our approach is more robust, as the atrial wall volume is generated directly from the image stack in a volumetric manner. This ensures that the initial volumetric representation of the atrial myocardium is topologically sound. Although our approach is less prone to topological issues, mesh topology errors may still occur at later stages, such as during surface smoothing operations aimed at removing jaggedness from the voxel-based grid or during remeshing to align mesh resolution with a prescribed target. To enhance robustness, a set of clean-up operations over the mesh elements - including removing intersecting elements, surface smoothing, improving mesh quality, and reindexing – is repeatedly applied after each critical meshing step. All 50 models in this study were meshed fully automatically, achieving element quality exceeding a threshold of 0.99.

### 4.4. Universal atrial coordinates

Anatomical reference frames are key for scalable modeling studies, as they provide a parametric encoding of all spatial model properties and, thus, facilitate an unattended parameter manipulation as required for parameter sweeps (Bayer et al., 2018; Roney et al., 2019a). Accurate and robust mapping based on such reference frames relies on features such as geometric linearity, that is, isolines are evenly distributed in space, and uniqueness to ensure that any parameter set encodes a unique location in space. For atrial models, the first universal spatial reference system has been reported for manifold models by Roney et al. (2019b). This approach was modified through anatomical normalization (Roney et al., 2021) and later extended to volumetric models (Roney et al., 2023). The volumetric extension relies on UACs defined on endocardial and epicardial manifold surfaces, which serve as boundary conditions for solving a LD problem on the volumetric mesh to achieve transmural interpolation. Additionally, by solving another LD problem, a normalized transmural distance field is computed.

While this approach is straightforward to implement, the generated volumetric UACs were of moderate quality. Shortcomings of this approach include manual selection of initial landmark on the LA and RA surface, which impedes a fully automated computation of the UAC, and the construction of geodesic path to define interfaces between lateral and septal, and anterior and posterior regions of the atria, to be used as boundary conditions for LD solves, which does not guarantee the consistent identifications of anatomical structures and reference point locations across different geometries. Moreover, UACs are always initially built and normalized over surface biatrial models and later extended to volumetric tissue by linear projection on the transmural mesh nodes, thus impairing a sound definition of transmural interfaces and UAC distribution. Lastly, the volumetric UACs were not tested across meshes with different resolutions.

Our approach overcomes these limitations by utilizing automatically selected anatomical landmarks and paths, and directly implementing UACs on volumetric meshes. We replace the anatomical paths previously used to divide the LA into posterior-anterior parts and the RA into lateral-septal parts with well-defined volumetric interfaces that separate not only the endo- and epicardial surface regions, but also the transmural mesh nodes. The computation of these interfaces is fully automated, based on pre-labeled anatomical regions and a set of rules to define reproducible points across anatomies. The resulting interfaces therefore ensure consistent identification of anatomical structures across different anatomical models. Moreover, they represent a well-defined transmural boundary for LD computation, thus avoiding previous ambiguity in the distribution of the transmural coordinate.

We demonstrate the robustness of our method for computing UACs by testing it on 50 biatrial geometries from real AF patients. Additionally, we show the applicability of our method across different mesh resolutions. The computation of our UACs is efficient on meshes suited for the R-E model, though it requires longer computational times when applied to finer grids appropriate for R-D models. The majority of the computational costs were attributed to solving the linear elasticity problem to normalize the UAC solutions. This was mainly due to the poorly deformed elements that could potentially result from the projection of the Laplace-Dirichlet solutions on the UAC subspace, and hinder the solver convergence. Enhancing the computational efficiency of UACs for finer meshes will be a focus of future work.

### 4.5. Modeling of inter-atrial connections

Electrical conduction between the atria is mediated by discrete ICs which play an important EP role. During normal sinus rhythm, the activation of the LA is mediated by ICs, determining the points of LA earliest activation which critically shape the mid- to terminal part of the P-wave. Further, ICs provide pathways for inter-atrial reentrant circuits, which represents a substrate for maintaining atrial flutter tachycardia.

Detailed histological studies identified five electrical main connections between the otherwise electrically isolated atria (Sakamoto et al., 2005): the BB, the coronary sinus bundle, an inferior-posterior bundle, a superior-anterior bundle, and the muscular ring around the fossa ovalis. These histo-anatomical insights are helpful for providing fundamental anatomical constraints to a model, but individual anatomy and EP properties may be highly variable. For instance, not all ICs may be present or electrically active in a given individual, the locations of RA origin and LA insertion, as well as the conduction properties of the ICs in between, may also vary to a significant extent. Thus, this leads to different ICs dominating inter-atrial activation and, thus, govern the site of early activation of the LA, or the RA for a retrograde atrial activation. As such, all factors defining the ICs must be considered variable, and, thus, flexibility represented to support their inference.

Three different approaches for incorporating the ICs in a biatrial model have been considered previously. These were: (i) timed electrical stimuli on the RA to mediate inter-atrial conduction, without including any ICs physical representation (ii) anatomical bridges, explicitly represented by meshing geometries of assumed ICs (Krueger et al., 2012; Wachter et al., 2015; Loewe et al., 2016), and, (iii) single ohmic resistor connecting RA and LA (Roney et al., 2021, 2023). All these approaches are limited in terms of flexibility and generalizability. Timed stimulation is flexible, but does not represent the mechanism mediating inter-atrial conduction, and as such does not generalize to physiological representations such as the atrial flutter. Explicit anatomical meshing is of limited flexibility and robustness, as any change in the origin or insertion site requires remeshing. This approach has been employed previously in Krueger et al. (2012), where, in absence of UACs, complex *ad-hoc* algorithms have been used to parameterize and explicitly ICs mesh (Wachter et al., 2015; Loewe et al., 2016). While feasible in principle, these approaches are complex and challenging to implement robustly, as the integration of thin inter-atrial strands with 3D atrial walls is highly prone to topological mesh errors. To provide an example, the explicit meshing of BB used in the study by Nagel et al. (2021) led to degenerated elements in all models throughout the cohort. Finally, fixed resistive 0D coupling, as used in various studies, using bilayer manifold models (Roney et al., 2021, 2023) is flexible, but the approach is not suitable for connecting volumetric atria due to dimensionality mismatch between 0D and 3D elements, which may cause source–sink mismatches. In principle, this problem may arise also in manifold models where 0D resistors are used to couple the two 2D surfaces representing RA and LA. In the absence of a physiological interpretation of the coupling resistor, viable ranges for which transduction between the atria is feasible have to be found by trial and error (Roney et al., 2023). Moreover, a further limitation is the inability to impose physiological constraints on the inter-atrial conduction delays, as transduction is rigidly dictated by the membrane time constant and the choice of coupling resistance.

Our novel approach to incorporating ICs in atrial models overcomes these limitations, as it is highly flexible, versatile, robust, and computationally efficient, and adds IC anatomy as a parameter that can be probed during model calibration. An arbitrary number of ICs can be included to connect arbitrary sites of origin and insertion on RA and LA, respectively, with prescribed conduction velocities governing the activation delay across the IC. The use of UACs to define origin and insertion sites, facilitates a seamless mapping of ICs between different anatomies. Further, modeling the ICs using a cable formulation computed with the same approach of generating the HPS, overcomes dimension mismatch problems between 1D and 3D model components.

### 4.6. Calibration of atrial EP models - using the P-wave as objective

Atrial EP modeling studies aiming to achieve a patient-specific calibration by 1:1 matching with direct observations – EAMs, EGMs or the ECG – are rare. Rather, adjustments of conductive and cellular dynamics properties informed by literature data are implemented to match global metrics such as the total atrial activation time. For a more accurate spatial calibration, EAM activation maps have been used to infer intrinsic tissue conductivities or conduction velocities (Lubrecht et al., 2021). High density EAM datasets covering both the RA and the LA appear ideal for calibrating atrial EP models, as they may provide a detailed view of the overall atrial activation sequence. However, while such datasets can be acquired, in principle, they are not widely available, as their acquisition tends to prolong procedures. Moreover, the spatiotemporal registration uncertainty between measurements of an EAM manifold and the image-derived model is significant, posing major challenges for calibration procedures. Using EGMs from which EAM maps are derived is even more challenging, as EGMs provide only a very local view on tissue activation. These may be helpful for inferring tissue characteristics from fibrotic patches, e.g. EGM magnitude and temporal separation of fractionated complexes in EGMs, correlate with the severity and structure of fibrosis, but do not offer benefits over using EAM maps directly.

In principle, the P-wave in the ECG appears a most natural choice for calibrating atrial EP models, as it is abundantly available and can be non-invasively recorded. However, the P-wave provides only a global view of the atrial activation sequence, and inferring space-varying model parameters from such limited data is challenging. Owing to the ill-posed nature of this inverse problem, this may not even be feasible. Also challenging but, in principle, feasible, as shown recently for the ventricular activation sequence (Gillette et al., 2021b; Grandits et al., 2024b), is the identification of model parameters that produce atrial activation sequences and replicate, with high fidelity, the P-wave in a clinical standard ECG. However, there may be more than one, potentially many, parameter sets yielding the same P-wave which raises questions of identifiability and uniqueness (Grandits et al., 2024a). Due to these challenges, only a few attempts have been made to calibrate an atrial EP model to the P-wave (Loewe et al., 2015), mostly limited to qualitative visual comparisons between simulated and recorded P-waves, without a quantitative assessment of the differences, as shown in this work.

Further obstacle to P-wave-based model calibration can stem from incomplete parametrization that fails to accurately produce the actual activation sequence, and insufficient computational performance that hinders a feasible calibration process, especially when relying on traditional R-D models (D.d. et al., 2012; Loewe et al., 2015; Ferrer et al., 2015; Fedele et al., 2023; Roney et al., 2023; Nagel et al., 2022) combined with high fidelity pseudo-bidomain or lower fidelity extracellular potential recovery models (Labarthe et al., 2014; Nagel et al., 2021), or limited fidelity in the predicted P-wave. Bilayer models are popular as they mitigate performance issues by reducing the overall problem size, but are still orders of magnitude slower compared to volumetric R-E models as used in this study, and the fidelity of predicted P-waves is limited. Volumetric biatrial R-D models immersed in a torso model using a full- or pseudo-bidomain are able to produce high-fidelity P-waves (Loewe et al., 2015; Ferrer et al., 2015). They offer the advantage of providing the entire potential field ϕe throughout the torso, in which ECG or more extensive body surface potential maps are embedded (Ferrer et al., 2015; Zappon et al., 2024). However, the approach is computationally by far too expensive for calibration studies, and the additional information on the torso potential field is not readily exploited in the absence of body surface potential mapping data.

Our analysis clearly demonstrates that the RELF model, combined with an extensive parameter space – including also the anatomy of the ICs – is able to produce activation sequences and associated P-waves with full bidomain fidelity, and at real-time performance. As shown previously for a ventricular ECG model (Gillette et al., 2021b), and in line with atrial ECG model (Nagel et al., 2022), the RELF model yields EGMs and ECGs that are not discernible from those produced with a full fidelity R-D bidomain model, as anticipated on theoretical grounds (Geselowitz, 1989). As shown in Fig. 10, differences in activation sequence and P-wave are minor, and can be deemed negligible in view of the overall model uncertainties.

In our study we focused on demonstrating the flexibility and efficiency of our framework in supporting comprehensive automated exploration of physical and geometrical parameter spaces. As proof-of-concept we demonstrated the feasibility of using our framework in a simple sampling-based optimization scheme to calibrate the atrial EP to match simulated to measured clinical P-wave. In four atrial anatomys of selected patients, 24 parameters were optimized, including conduction velocities in different anatomical regions, six possible activation sites corresponding to SAN exit sites on the RA, and the entry sites of the ICs on the LA. While achieving a good fit with the corresponding clinical P-wave for all patients, differences in P-wave morphology were primarily observed in leads V1 and V2, and in one subjects on the limb leads. These might have different causes. As the atrial model was integrated with torso geometry and electrode positions taken from a different subject, and none of the pathologies of the patient treated by AF ablation were considered, discrepancies can be anticipated, and the feasibility of achieving a good fit is not even guaranteed. Nonetheless, P-wave features such as positivity in all leads, as well as the biphasic characteristic of lead III and V1 on some patients, were correctly replicated by the model.

Importantly, the fidelity and efficiency of our framework support, in principle, a comprehensive exploration of the parameter space spanned by atrial anatomy and EP, and, thus, provides a basis for building future applications geared towards creating digital twins based on P-wave calibration. A full single forward simulation lasted ≈ 27.00s only where the actual evaluation of the EP model amounted only to ≈ 3.00s.

### 4.7. Role of left and right atrial electrophysiology in the genesis of the P-wave

Despite the vast acceleration in speed achieved with high fidelity EP forward models, such as the RELF, the use *a priori* knowledge on the genesis of the P-wave is key to constrain the inference by limiting the admissible parameter space and the potentially large number of different atrial activation sequences that produce the same P-wave.

From a macroscopic perspective, the genesis of the P-wave is well understood. However, at a mesoscopic size scale, that is the relation between the physics of depolarization wavefronts traversing individual atrial structures, and their relative contribution to the global P-wave, is still unclear. These aspects have been investigated only in a limited number of studies (Loewe et al., 2015, 2016; Ferrer et al., 2015). In Loewe et al. (2015), the separate contribution of RA and LA depolarization to the P-wave was investigated in two atrial and torso models derived from healthy subjects. Using a pre-defined activation sequence, the study showed that the RA predominantly influences the P-wave in precordial leads V1 and V2, as well as limb leads II, aVF, and III, while the LA governs the central to terminal portion of the P-wave. The effect of varying the atrial activation sequence on P-wave features such as positivity or negativity in different leads was investigated in Loewe et al. (2016) in eight biatrial models of healthy subjects by initiating atrial activation at a fixed set of locations representing the SAN exit sites. The relative contributions of different atrial regions to the P-wave was moreover analyzed in Ferrer et al. (2015), for a fixed atrial activation sequence, with prescribed SAN exit sites and IC locations.

In our study, we investigate the envelope of P-waves associated with changes in both RA and LA anatomical and physiological factors, including the location and number of early activation sites in the LA, the position and shape of the SAN, and the conduction velocity in the fiber and sheet directions of the RA body. By separately analyzing the ECG variations due to these factors, we also highlight the roles of RA and LA activation in shaping the P-wave.

The generated P-wave envelop did not fully cover the P-wave observed in the modeled patient, most evident in the leads aVL and aVR (refer to Fig. 14). The terminal phase of the P-wave is not well covered, most prominently visible in the more lateral precordial leads V3-V6, and in the leads aVR and II. This discrepancy may stem from various factors. Firstly, the model was built from a patient treated by AF ablation, with a significant fibrotic burden, which remained unaccounted for in our model, and was instead assumed to be structurally healthy. As such, the dipoles at the interface between healthy atrial tissue and fibrotic patches are missing, leading to a nearly zero dipole once both atria were fully activated. Finally, torso and lead positions were taken from another subject and were not specific to the given subject.

#### 4.7.1. Discrete interatrial conduction

The site of earliest activation of the LA is governed by the RA activation sequence and the location and conductive properties of the ICs. In this study, we only investigated the effect of varying location and size of the insertion site of BB on the anterior wall of the LA upon the P-wave. The use of bundles for modeling ICs facilitates automated sweeps over these important parameters that govern the coupling spatio-temporal coupling of RA and LA. Consistent with Loewe et al. (2015), our simulations show that LA activation influences only the mid to terminal portion of the P-wave (see Fig. 11). Major differences due to varying BB insertion in the LA were witnessed in leads aVL, I, aVR, and III, while effects in other limb leads and precordial leads were minor. Using three ICs to model a fan-like insertion of the BB into the anterior LA had led only to a minor spread in the P-wave envelope (see Fig. 12).

#### 4.7.2. Sino-atrial node

The SAN was originally described as a crescent-shaped area at the junction of the SVC and the RA (Keith and Flack, 1907; Anderson et al., 1983), and later found to extend along the TC, and towards the IVC (Monfredi et al., 2010; Boyett et al., 2000; Anderson et al., 1978; Akima, 1978; Csepe et al., 2016; Fedorov et al., 2012). Normal SAN automaticity depends on its depolarized resting potential of ≈ −60.00mV relative to neighboring atrial tissue, with a more hyperpolarized resting potential of ≈ −85.00mV (Csepe et al., 2016). To prevent hyperpolarization of SAN and, thus, inhibition of automaticity, the SAN is electrically mostly insulated to safeguard pacemaker function (Joyner and Van Capelle, 1986; Fedorov et al., 2012). Electrical coupling of the SAN with surrounding atrial myocardium is limited to specific discrete sites, referred to as SAN exit pathways, which can number up to five (Li et al., 2017; Fedorov et al., 2010). As such, the location of exit pathways and their activity determine the earliest atrial activation site and the shape of the initial depolarization wavefront, which not only affects the activation sequence of the RA, but may also influence the order of activation of the ICs, and, in turn, alter the earliest activation site on the LA (Li et al., 2017; Antz et al., 1998b). Consequently, SAN anatomy and exit pathways are crucial parameters to consider when modeling atrial activation and its reflection in the P-wave.

In modeling studies, the SAN is often represented as a spherical region at a fixed location (Loewe et al., 2016; Ferrer et al., 2015; Fedele et al., 2023), with only a few studies accounting for the anatomical shape of the SAN (Labarthe et al., 2014; Gillette et al., 2022). In line with previous work (Loewe et al., 2016; Ferrer et al., 2015), here we demonstrate a marked dependency of the P-wave upon exit sites (refer to Fig. 14). By easy variation of SAN size and location, we show that our framework may easily allow for inferring both SAN anatomy and exit sites based on ECG calibration.

#### 4.7.3. Effect of the RA endocardium on the P-wave

The anterior and lateral endocardial walls of the RA are primarily composed of PMs and TC tissue and are attached to a thin-walled LA epicardium, consisting of only a few layers of myocytes (T.a. et al., 2004; Lang et al., 2022). While explicitly accounted for in bilayer models, this is often not the case in volumetric models (Roney et al., 2023). There, two different approaches have been used. Either, TC, PMs and the RA portion of BB were explicitly meshed, or a continuous right endocardial layer of prescribed thickness was generated to represent the RA endocardium, onto which the locations of the TC and PMs are projected either by using an anatomical atlas, or on a per-rule basis. Atrial EP in the RA endocardium is then simulated by assigning faster conduction velocities in the TC and PMs, and slower conduction velocities to the remaining endocardial tissue between the protruding structures (Loewe et al., 2015; Ferrer et al., 2015; Azzolin et al., 2023; Roney et al., 2023). As such, the absence of tissue between the TC and PMs is typically not accounted for which can be interpreted as an increase in the effective epicardial wall width and the associated source strength.

We investigate the effect of modeling the RA endocardial tissue as an electrically active layer upon atrial sources and the P-wave. Treating the RA endocardial layer in between TC and PMs as blood pool slowed down the activation of the posterior-lateral wall of the RA and the RAA. While this had a minor impact on the total activation time of the RA, a noticeable reduction in peak P-wave amplitude by ≈1% of was witnessed, with no change in P-wave morphology. The observed deviations in P-wave can be deemed as minor relative to the overall uncertainty. This may be not the case when simulating endocardial recorded EGMs, when electrodes are located near the atrial sources.

## 5. Limitations

While our comprehensive end-to-end workflow for generating biatrial anatomies combined with a fast forward RELF model is able to deliver anatomically accurate models for predicting ECG and EGMs with full physical fidelity, numerous notable limitations exist.

Our study introduces a technological framework for the calibration of atrial EP models using observable extracellular recordings such as the EGMs and the ECG – specifically, the P-wave – as objective to infer the high dimensional parameter space of such models. While we show that employing a brute force sampling approach as in Gillette et al. (2021b) an initial calibration of the P-wave is feasible, the computational costs to achieved these results are extremely high. Similar to previous work on identifying the ventricular activation sequence, a dedicated approach combining *a priori* knowledge to constrain the search space in combination with an optimization approach that facilitates a gradient-based search for parameters is likely to be more accurate and efficient (Grandits et al., 2021, 2024b). Sampling narrow, physiologically constrained parameter corridors, as done here, may provide a better approximation than current standard methods. However, this improvement cannot be guaranteed for two key reasons. First, as the atrial activation sequence cannot be measured accurately, not even invasively with most advanced EAM methodology, a solid ground truth reference is missing. Secondly, it is highly likely that a large number of parameter choices exist which yield identically perfect matches of the ECGs. It is therefore important to note that the achieved close match in P-wave morphology does not imply that the activation sequence of the calibrated model is an accurate representation of the real physical activation of the patient’s atria. Thus, uniqueness and identifiability is a serious concern that must be adequately addressed to narrow down the number of different sampled parameters. The development of suitable strategies and their evaluation was beyond the scope of this study, and substantial further research efforts are required.

An important factor limiting the anatomical accuracy of models generated by our end-to-end workflow is the assumption of uniform wall thicknesses. As the accurate segmentation of the atrial walls from current clinical images is challenging, in our volumetric wall reconstruction, the atrial wall widths were prescribed and held constant over individual anatomical regions. However, atrial wall widths in a patient’s heart may be different and more heterogeneous than represented in the model. As a consequence, the local variation in local conduction velocity mediated by altered source–sink relation may not be accurately represented.

A further limitation is the reduced computational efficiency in generating finer resolutions for R-D models. This is largely caused by the computation of the UACs, and not due to the extra cost of generating finer meshes per se. Specifically, projecting the biatrial mesh onto the UAC space to solve the linear elasticity problem can induce mesh deformations and, consequently, iterative solver convergence issues. More advanced preconditioners will be incorporated (Augustin et al., 2016) in the future to enhance computational efficiency. Due to the nearly identical anatomical meshes, a faster alternative may be the Euclidian interpolation of UACs from the lower resolution R-E to the higher resolution R-D mesh, but this has not been investigated here.

While our novel flexible and rapid approach to modeling ICs is advantageous for model calibration, the used of single cables only approximates the effects of conduction between the atria, but not the actual sources contributing to the extracellular potential fields. The actual anatomical structures underlying the ICs are rather muscular bands or 3D strands. As such, the volumetric source density of the cable approach underestimates the contribution to the potential field in their vicinity. While this does not constitute a problem for simulating activation sequences and the ECG, simulating the sensing of EGM will be inaccurate and also methodologically challenging to implement, owing to the non-conformity of IC grid with the atrial or the volume conductor mesh. This problem is readily mitigated by using bundles instead of single cables, which may be straightforwardly incorporated, as shown for modeling a fan-like insertion of the BB, where a bundle of three ICs was used (see Fig. 12). Alternatively, the cable-based representation can be replaced with an explicit mesh of the ICs once the model has been calibrated.

Further, the cables representing the ICs are automatically generated, following the geodesic paths connecting two mesh nodes on RA and LA, respectively. This might result in a mismatch in trajectory and length between modeled and real IC and, thus, physiologically non-feasible conduction velocities may be required to meet prescribed inter-atrial conduction delays. Specifically, this may complicate the transfer of parameters from R-E to R-D models where conduction velocity ranges are limited.

Further, we have not included the segmentation of the torso along with the corresponding atrial geometries. Large clinical data cohorts often lack CT or MRI scans of the torso. Nevertheless, our workflow supports the meshing of a generic torso surface around the generated atrial geometries, making the integration of automatic torso segmentation and corresponding meshing into our workflow a straightforward enhancement.

Finally, while all anatomical models were generated from clinical data acquired from AF patients undergoing ablation therapy, no information on the underlying pathology have been taken into consideration. Activation mapping reveals altered atrial conduction properties, substrate mapping is indicative of fibrotic remodeled tissue, and the signal fractionation in EGM recordings during AF episodes carry information on the structure of the fibrotic substrate. These data could be used in two ways, for improving P-wave prediction and for an independent validation of the calibrated model. However, in this study only routine clinical data were used which are largely insufficient for a thorough validation as coverage and density of acquired maps is limited. Further, more complex reentrant AF-like activation patterns cannot be simulated efficiently with the RELF forward model which does not support multi-frontal wave propagation. However, such simulation technologies have been developed (Barrios Espinosa et al., 2025) and integrated into widely available simulation platforms such as openCARP (Plank et al., 2021).

Advancements in altering atrial depolarization profiles may be necessary. Furthermore, real damaged tissue data from clinical sources is not yet integrated into our workflow. However, our reference framework is designed to easily accommodate new functionalities and define complex parameter variations, paving the way for the automatic and near-real-time creation of atrial digital twins.

## 6. Conclusions

In this study, we developed an automated, efficient, and flexible multi-scale workflow for creating anatomically accurate biatrial models with high-quality meshes, suitable for various forward EP models, and extended it to a function twinning stage, where efficient model calibration based on ECG data is possible. The workflow utilizes a segmented blood pool and a deep learning-based segmentation network to automatically identify and label principal atrial structures. It moreover generates endocardial and epicardial walls based on predefined rules, ensuring robust model openings and high mesh quality. Detailed anatomical and functional structures, including fiber architecture and space-varying parameters, are incorporated into the models. We further introduced a flexible method for representing ICs using auto-generated cables, allowing for realistic and customizable conduction properties. Our framework finally includes a clinically compatible ECG forward generation system, integrating both R-D and R-E models with Lead Field methods for accurate ECG trace generation, and possibly allows for the integration of the Pseudo-bidomain approach or $\phi_{e}$-recovery. Among these, we show that the RELF method proved effective for real-time ECG computation and reliable source approximation in sinus rhythm.

Overall, our workflow facilitates efficient exploration of the EP parameter space for calibration of biatrial models based on ECG data, making it a valuable tool for advancing cardiac EP research.

## Figures and Tables

**Table 1:** DBCs for the 15 LD problems employed to determine atrial anatomical structures and tissues known to have different EP properties.

Atrium	φ	$\varphi_{1}$	$\Gamma_{1}$	$\varphi_{2}$	$\Gamma_{2}$	$\varphi_{3}$	$\Gamma_{3}$	$\varphi_{4}$	$\Gamma_{4}$

RA	$\varphi_{\mathrm{trans}}$	0	$\Gamma_{\mathrm{endo}}$	1	$\Gamma_{\mathrm{epi}}$				
	$\varphi_{ab}$	−1	$\Gamma_{\mathrm{raa}}$	0	$\Gamma_{\mathrm{svc}}$	1	$\Gamma_{tv_{l}}\cup \Gamma_{tv_{s}}$	2	$\Gamma_{\mathrm{ivc}}$
	$\varphi_{v}$	0	$\Gamma_{\mathrm{svc}}\cup \Gamma_{\mathrm{raa}}$	1	$\Gamma_{\mathrm{ivc}}$				
	$\varphi_{v2}$	0	$\Gamma_{\mathrm{ivc}}$	1	$\Gamma_{\mathrm{raa}}$				
	$\varphi_{v3}$	0	$\Gamma_{\mathrm{svc}}$	1	$\Gamma_{\mathrm{ivc}}$				
	$\varphi_{r}$	0	$\Gamma_{\mathrm{roof}}$	1	$\Gamma_{tv_{l}}\cup \Gamma_{tv_{s}}$				
	$\varphi_{r2}$	0	$\Gamma_{\mathrm{svc}}\cup \Gamma_{\mathrm{roof}}\cup \Gamma_{\mathrm{ivc}}$	1	$\Gamma_{tv}$				
	$\Gamma_{w}$	−1	$\Gamma_{tv_{l}}$	1	$\Gamma_{tv_{s}}$				
	$\Gamma_{w2}$	−1	$\Gamma_{tv_{l}}$	0	$\Gamma_{\mathrm{roof}}$	1	$\Gamma_{tv_{s}}$		

LA	$\varphi_{\mathrm{trans}}$	0	$\Gamma_{\mathrm{endo}}$	1	$\Gamma_{\mathrm{epi}}$				
	$\varphi_{\mathrm{ab}}$	−1	$\Gamma_{\mathrm{laa}}$	0	$\Gamma_{\mathrm{lpv}}$	1	$\Gamma_{mv}$	2	$\Gamma_{rpv}$
	$\varphi_{\mathrm{ab}2}$	0	$\Gamma_{\mathrm{rpv}}$	1	$\Gamma_{\mathrm{laa}}$				
	$\varphi_{v}$	0	$\Gamma_{lpv}$	1	$\Gamma_{rpv}$				
	$\varphi_{r}$	0	$\Gamma_{rpv}\cup \Gamma_{lpv}\cup \Gamma_{mv}$	1	$\Gamma_{\mathrm{mv}}$				
	$\varphi_{r2}$	0	$\Gamma_{rpv}\cup \Gamma_{\mathrm{lpv}}$	1	$\Gamma_{mv}$				

**Table 2:** Dirichlet boundary conditions for the Laplace problems that are solved to obtain the preliminary UAC space in the septal and lateral RA and the posterior and anterior LA where s∈[0,1] is a parametrization value of the corresponding boundary surface and interface, respectively. In our framework, R = 0.1.

	$\Omega_{RA,\mathrm{sept}}$ and $\Omega_{\mathrm{LA,}\mathrm{ant}}$		$\Omega_{R\mathrm{A,}\mathrm{lat}}$ and $\Omega_{LA,post}$	
		
	α	β	α	β

$\Gamma_{tv}$, $\Gamma_{\mathrm{mv}}$	–	1	–	0
$\Gamma_{\mathrm{ivc}}$, $\Gamma_{\mathrm{lspv}}$	$R\;cos\left((1-s)\frac{\pi }{2}\right)$	$\frac{1}{2}+R\;sin\left((1-s)\frac{x}{2}\right)$	$R\;cos\left((1-s)\frac{\pi }{2}\right)$	$\frac{1}{2}+R\;sin\left((3+s)\frac{\pi }{2}\right)$
$\Gamma_{\mathrm{svc}}$, $\Gamma_{rspv}$	$1+R\;cos\left((1+s)\frac{x}{2}\right)$	$\frac{1}{2}+R\;sin\left((1-s)\frac{x}{2}\right)$	$1+R\;cos\left((1+s)\frac{\pi }{2}\right)$	$\frac{1}{2}+R\;sin\left((3+s)\frac{\pi }{2}\right)$
${ℐ}_{tv,ivc}$, ${ℐ}_{mv,\mathrm{ls}pv}$	0	$\left(\frac{1}{2}+R\right)+(1-s)\left(\frac{1}{2}-R\right)$	0	$s\left(\frac{1}{2}-R\right)$
${ℐ}_{\mathrm{tv,suc}}$, ${ℐ}_{\mathrm{mv,rspv}}$	1	$\left(\frac{1}{2}+R\right)+(1-s)\left(\frac{1}{2}-R\right)$	1	$s\left(\frac{1}{2}-R\right)$
${ℐ}_{\mathrm{ivc},\mathrm{svc}}$,${ℐ}_{\mathrm{lspv,rspv}}$	R + (1 − 2 R) *s*	2	R + (1 − 2 R) *s*	$\frac{1}{2}$

**Table 3:** Final position of the CS, LIPV, and RIPV orifices with parametrization value s∈[0,1].

	α	β

$\Gamma_{cs,\mathrm{roof}}$	$\alpha_{cs}+r\;cos((2-s)\pi )$	$\beta_{cs}+r\;sin((2-s)\pi )$
$\Gamma_{cs,tv}$	$\alpha_{cs}+r\;cos(s\;\pi )$	$\beta_{cs}+r\;sin(s\;\pi )$
$\Gamma_{\mathrm{lipv,roof}}$	$\alpha_{ipv}+r\;cos(s\;\pi )$	$\beta_{ipv}+r\;sin(s\;\pi )$
$\Gamma_{lipv,mv}$	$\alpha_{ipv}+r\;cos((2-s)\pi )$	$\beta_{ipv}+r\;sin((2-s)\pi )$
$\Gamma_{\mathrm{ripv,roof}}$	$\left(1-\alpha_{ipv}\right)+r\;cos(s\;\pi )$	$\beta_{ipv}+r\;sin(s\;\pi )$
$\Gamma_{\mathrm{ripv,mv}}$	$\left(1-\alpha_{ipv}\right)+r\;cos((2-s)\pi )$	$\beta_{ipv}+r\;sin((2-s)\pi )$

**Table 4:** Conduction velocities in longitudinal and transverse directions, $v_{l}$ and $v_{t}$, prescribed in the R-E model along with calibrated conductivities yielding matching velocities in the R-D models at the target average mesh resolution of Δx≈0.25mm. The bidomain surface-to-volume ration was chosen as $\beta =1400.00{\;cm}^{-1}$.

	$v_{l}\;m/s$	$v_{t}\;m/s$	$g_{i,l}\;S/m$	$g_{e,l}\;S/m$	$g_{i,t}\;S/m$	$g_{e,t}\;S/\mathrm{mx}$

RA	0.97	0.74	0.583	0.742	0.232	1.162
LA	0.98	0.76	0.570	0.726	0.220	1.102
TC	1.21	0.92	0.904	1.112	0.416	0.899
PMs	1.30	0.99	1.053	1.339	0.408	2.042
BB (atrial)	1.40	1.08	1.229	1.564	0.479	2.399
Rim FO	0.33	0.24	0.080	0.102	0.034	0.168

**Table 5:** Bounds defined for varying the parameters in $\omega_{\mathrm{opt}}$.

	CV [m/s]	α	β	t[ms]

ratio	[1.0, 1.6]			
TC	[1.0, 2.4]			
PM	[1.0, 2.4]			
BB	[1.0, 2.4]	[0.345, 0.84]	[0.49, 0.97]	[30, 100]
RA	[0.8, 1.8]			
LA	[0.8, 1.8]			
SAN		[0.20, 0.92]	[0.32, 0.7]	[0, 50]
CS		[0.33, 0.82]	[0.7, 0.93]	[50, 120]
PC		[0.58, 0.84]	[0.22, 0.45]	[50, 100]

**Table 6:** Average timings for the generation of all the 50 biatrial geometries at target resolutions of ≈ 0.90 mm and ≈ 0.25 mm and the number of models requiring manual corrections are given for each processing stage.

	Mesh resolution/Model		Manual Correction
		
	0.90mm/R-E	0.25 mm/R-D	# Cases

Automatic multilabel segmentation	≈ 15 s	≈ 15 s	19
Label augmentation on the blood pools	297 ± 180 s	297 ± 180 s	22
Walls extrusion and meshing	187 ± 172 s	628 ± 557 s	0
Selection of the atrial orifices	4 ± 6 s	39 ± 36 s	0
Anatomical structures and fibers	30 ± 35 s	1084 ± 628 s	0
Universal atrial coordinates	8 ± 14 s	175 ± 37 min	0

Total processing time	9 ± 7 min	209 ± 51 min	–

**Table 7:** Execution times of setup and simulation using R-E or R-D models.

	Mesh resolution/Model	
	
	0.90 mm/R-E	0.25 mm/R-D

Volumetric torso	≈ 2 min 25 s	≈ 22 min 30 s
Interatrial connections	≈ 19 s	≈ 39 s
Lead field	≈ 35 s	≈ 9 min
EP simulation	≈ 27 s	≈ 19 min

**Table 8:** RMSE in percentage, between ECG signals computed with the R-E and R-D models coupled with the lead field. The PWD of the ECG traces obtained with the R-E and the R-D models coupled with the lead field are also reported.

Lead	aVL	I	-aVR	II	aVF	III	V1	V2	V3	V4	V5	V6	Average

RMSE [%]	3.56	3.44	3.54	5.16	4.87	5.17	4.64	3.66	3.09	1.69	2.39	2.34	3.71
PWD R-E [ms]	128	131	131	125	129	89	126	131	123	126	131	131	125.1
PWD R-D [ms]	121	124	121	124	129	90	120	131	123	126	131	131	122.6

**Table 9:** Maximum and minimum PWD, and their differences, for each lead and averaged over all leads.

Lead	aVL	I	-aVR	II	aVF	III	V1	V2	V3	V4	V5	V6	Average

Min PD [ms]	93	93	108	110	104	107	99	106	106	106	106	106	104,2
Max PWD [ms]	117	115	124	126	119	120	118	118	118	124	124	123	120,5
(Max – Min) PWD [ms]	22	16	13	16	24	15	19	12	12	18	15	14	16,3

**Table 10:** Maximum and minimum PWD, and their differences, for each lead and averaged over all leads.

Lead	aVL	I	-aVR	II	aVF	III	V1	V2	V3	V4	V5	V6	Average

Min PD [ms]	80	100	107	110	95	107	94	98	110	110	109	109	102,4
Max PWD [ms]	89	114	115	118	99	115	104	108	113	113	115	116	109,9
(Max – Min) PWD [ms]	9	14	8	8	4	8	10	10	3	3	6	7	7,5

**Table 11:** RMSE between the ECGs obtained when excluding the RA endocardial tissue outside of the PMs and TC, and the ECGs signal obtained including the RA endocardial tissue, and PWD, for each lead and averaged over all leads.

Lead	aVL	I	-aVR	II	aVF	III	V1	V2	V3	V4	V5	V6	Average

RMSE [%]	0,08	0,63	0,91	1,19	0,88	0,58	1,31	1,18	0,85	0,72	0,58	0,49	0,78
PWD [ms]	114	119	120	121	97	89	111	86	88	99	101	121	105,5

**Table 12:** Minimum RMSE, in percentage, PWD of the ECG data, of the computed ECG with minimum RMSE, and their difference, obtained by varying the position of the spherical or cylindrical activation impulse representing the SAN, and the conduction velocity $v_{f}$ and $v_{s}$ of the RA.

Lead	aVL	I	-aVR	II	aVF	III	V1	V2	V3	V4	V5	V6	Average

min RMSE [%]	1.16	1.68	2.27	2.06	1.33	1.02	2.60	1.51	2.22	1.87	1.58	1.37	1.72
PWD [ms]													
Clinical data	139	147	123	124	127	146	134	125	118	132	120	129	130.33
Simulated ECG	136	137	135	150	133	151	145	142	131	129	131	151	139.25
Diff with data	3	10	12	26	6	5	11	17	13	3	11	22	8.92

**Table 13:** Optimal values for the parameters of $\omega_{\mathrm{opt}}$ for each calibrated patient.

	P1	P2	P3	P4

${CV}_{\mathrm{ratio}}$	1.6	1.6	1.6	1.6
${CV}_{TC}[m/s]$	1.65	1.75	1.0	1.56
${CV}_{PM}[m/s]$	1.03	1.00	1.0	2.26
${CV}_{BB}[m/s]$	1.00	1.61	1.18	1.00
${CV}_{RA}[m/s]$	0.80	0.80	0.80	0.80
${CV}_{LA}[m/s]$	0.80	0.80	0.88	1.49
$\alpha_{SAN}^{1}$	0.59	0.45	0.78	0.59
$\beta_{SAN}^{1}$	0.32	0.38	0.37	0.42
$t_{\mathrm{SAN}}^{1}[ms]$	22.27	29.82	16.80	29.53
$\alpha_{SAN}^{2}$	0.57	0.53	0.49	0.29
$\beta_{SAN}^{2}$	0.34	0.57	0.52	0.42
$t_{SAN}^{2}[ms]$	34.95	30.0	25.58	38.53
$\alpha_{BB}^{1}$	0.62	0.80	0.83	0.80
$\beta_{BB}^{1}$	0.73	0.52	0.65	0.79
$t_{BB}^{1}[ms]$	60.04	89.79	72.85	30.03
$\alpha_{BB}^{2}$	0.69	0.57	0.55	0.55
$\beta_{BB}^{2}$	0.49	0.65	0.70	0.88
$t_{BB}^{2}[ms]$	78.23	74.73	70.06	49.95
$\alpha_{BB}^{3}$	0.75	–	–	–
$\beta_{BB}^{3}$	0.88	–	–	–
$t_{BB}^{3}[ms]$	57.81	–	–	–
$\alpha_{CS}$	0.49	0.90	0.73	0.61
$\beta_{CS}$	0.02	0.13	0.22	0.25
$t_{CS}[ms]$	81.49	59.36	71.06	50.02
$\alpha_{PC}$	–	0.84	–	0.84
$\beta_{PC}$	–	0.39	–	0.45
$t_{PC}$ [ms]	–	73.25	–	64.86

**Fig. A.16.**
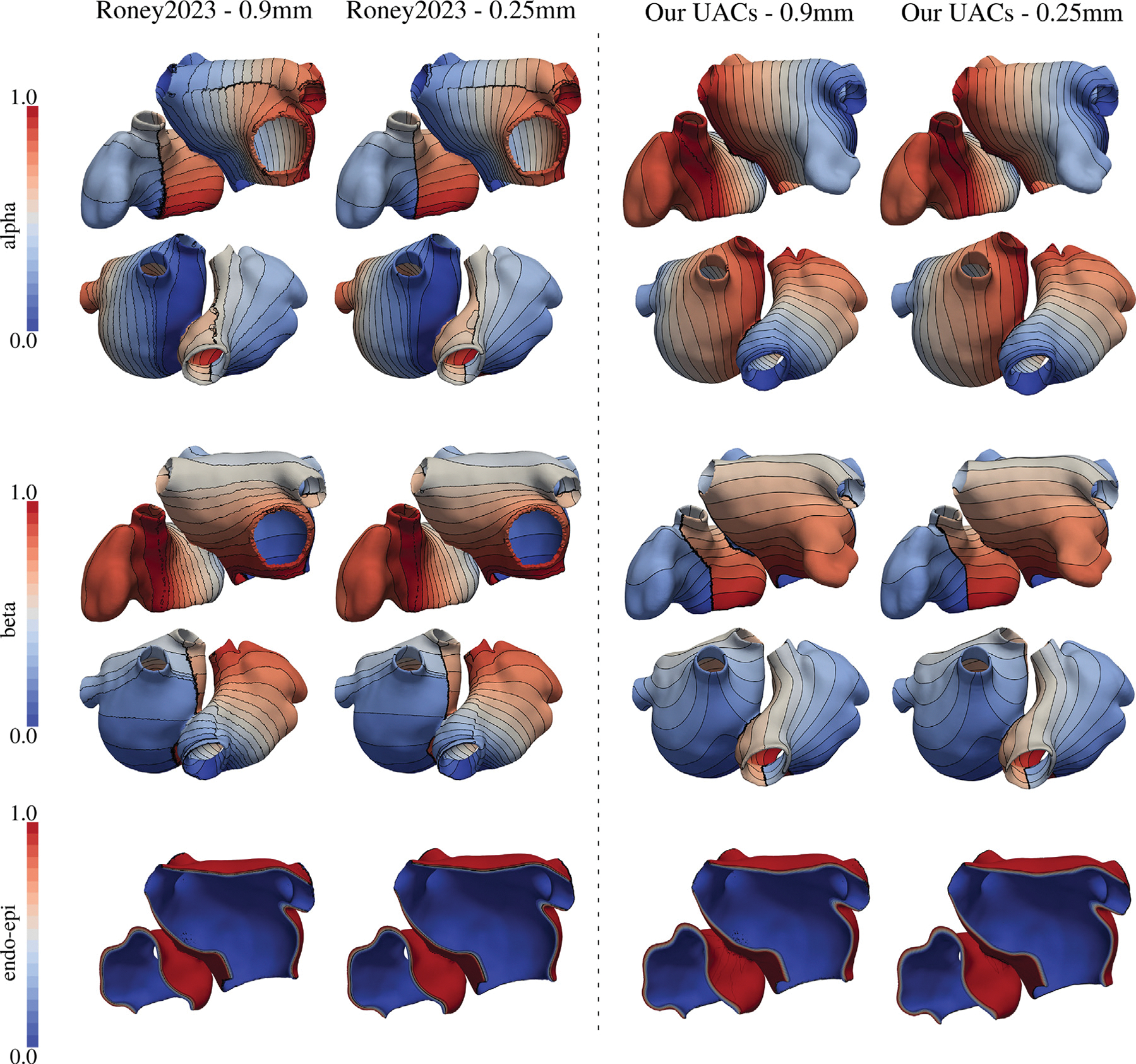
Qualitative comparison between the UAC generated on a model randomly selected in the AF dataset employing the method and code presented by Roney and coauthors in Roney et al. (2019a) (left), and with the method presented in this work (right), with a mesh resolution of 0.90mm (first and third column) and 0.25mm (second and fourth column).

## Appendix. UAC comparison

We compared our UAC generation approach with the publicly available software developed by Roney and coauthors in Roney et al. (2023), hereafter referred to as the Roney2023 approach. In Roney2023, UAC computation was integrated into a workflow for generating biatrial bilayer surface models. Volumetric models were generated only from a statistical shape model atlas and used in conjunction with the available software to produce volumetric UACs. More specifically, given a volumetric mesh and the corresponding endocardial and epicardial surfaces, the following steps are required:

- Manually select four sets of reference points — two for the RA and two for the LA — according to the package guidelines;
- Compute the anatomical regions of the atria on both the endocardial and epicardial surfaces, along with the corresponding normalized UACs;
- Extend the UACs to the volumetric mesh by interpolating the surface UAC.

While here presented as part of the proposed atrial model generation workflow, our UAC implementation can also be used as a standalone tool to generate a UAC system directly from a volumetric mesh, potentially already annotated with anatomical region labels.

We randomly selected one atrial anatomical model from those generated using our workflow within the AF dataset employed in this study. UACs were computed on the selected model at the two mesh resolutions of 0.90mm and 0.25mm, using both the Roney2023 software and our proposed tool. The comparison is presented in terms of user interaction, user-friendliness and reproducibility, qualitative outcomes, and computational time. For the latter, our UAC tool was executed in standalone mode to specifically measure the time required for UAC system generation of this anatomical model.

We observed several usability limitations in the Roney2023 software, primarily due to the absence of a streamlined script capable of automatically handling all the required steps for UAC system generation. Specifically, the three aforementioned steps must be executed manually by the user through four separate scripts, which must be run in a specific sequence. Filenames and paths are hardcoded across multiple source files, requiring manual editing of at least the main executables to correctly specify input files and directories. In contrast, our tool supports a fully integrated workflow that can be executed with a single terminal command, requiring only the path and filename of the input volumetric mesh.

Moreover, the Roney2023 software does not provide automated extraction of the endocardial and epicardial surfaces from a given volumetric mesh. These surfaces must instead be provided as input by the user, necessitating additional preprocessing steps. As a result, users must manually extract the atrial surfaces, a task that relies on user expertise and contributes to increased overall processing time. Conversely, our implementation includes automatic detection of anatomical regions, as well as extraction of the endocardial and epicardial surfaces, when these are not explicitly provided by the user.

While the Roney2023 software supports the generation of the UAC on a bilayer surface mesh of the LA that includes the LAA, this functionality does not appear to be available in the volumetric UAC implementation. Additionally, the software assumes the CS to be closed, treating it as part of the RA wall. To enable UAC computation on our anatomical models using this software, we manually modified the input model by closing the CS and removing the LAA, based on precomputed anatomical landmarks. Although our tool does not currently support the exclusion of the LAA during UAC computation, it does allow for the generation of UACs on the RA regardless of the presence or absence of the CS. This flexibility is particularly valuable in practical scenarios, as the CS is not always visible in image-based segmentations due to variability in imaging quality.

Furthermore, two of the four manually selected sets of landmarks are directly utilized to define the boundary conditions for the Laplace solutions used in UAC generation. As a result, the accuracy and reliability of the computed UACs are highly dependent on the user’s experience and judgment in selecting appropriate reference points, which may negatively affect reproducibility across different users and datasets. In contrast, our approach includes an automated selection of reference points and computation of the corresponding paths, thereby enhancing the robustness of the method and facilitating reproducible results across applications.

A qualitative comparison of the generated UAC at both mesh resolutions is presented in Fig. A.16. Although we successfully computed UAC using both the Roney2023 method and our approach at both resolutions, notable differences were observed in the quality of the distribution and the mesh dependency of the resulting UACs. In particular, the UAC generated with the Roney2023 method showed issues on the posterior wall of both the LA and RA. Misalignment of the α UAC was visible across the roof of the LA, and uniqueness of the coordinate was not maintained in the SVC-IVC region. In contrast, our method preserved the uniqueness of the α coordinate across all boundaries, achieved a more uniform distribution, and ensured value consistency across regions.

**Table A.14:** Execution times for computing the UAC system with Roney2023 method (first row), and our method (second row), at mesh resolution of 0.90 mm (second column), and 0.25 mm (third column).

	res = 0.90 mm	res = 0.25 mm

Roney2023	10.00 min 15.00 s	140.00 min 42.00 s
Our UACs	8.00 s	100.00 min 44.00 s

Transmurally, Roney2023 UAC showed irregularities, particularly in the mid-wall regions, likely due to the smoothing effects of the surface-based interpolation used to extend the coordinates into the volume. Our transmural coordinate, on the other hand, remained well distributed throughout the entire domain. Moreover, the quality of the UAC generated by the Roney2023 method showed a clear dependency on mesh resolution, as evidenced by the non-smooth isolines in the first column of Fig. A.16, with noticeable improvements at finer resolutions. In contrast, our method produced UACs of consistently high quality in different mesh resolutions, with minimal visual distinction between them.

Finally, the computational times required to compute the UACs using both the Roney2023 and our methods at the two mesh resolutions are reported in Table A.14, using the same machine for all tests. Our implementation consistently outperformed the Roney2023 method, achieving a reduction in computational time of approximately 98.7% for the 0.90mm mesh resolution and 28.4% for the 0.25mm mesh resolution.

## References

[R1] Akima H, 1978. A method of bivariate interpolation and smooth surface fitting for irregularly distributed data points. ACM Trans. Math. Softw. (TOMS) 4, 148–159.

[R2] Anderson RH, Ho SY, Becker AE, 1983. The surgical anatomy of the conduction tissues. Thorax 38, 408–420. 6348996 10.1136/thx.38.6.408 PMC459576

[R3] Anderson RH, Yen H.Siew., Becker AE, Gosling JA, 1978. The development of the sinoatrial node. In: The Sinus Node: Structure, Function, and Clinical Relevance. Springer, pp. 166–182.

[R4] Antz M, Otomo K, Arruda M, Scherlag BJ, Pitha J, Tondo C, Lazzara R, Jackman WM, 1998a. Electrical conduction between the right atrium and the left atrium via the musculature of the coronary sinus. Circulation 98, 1790–1795. 9788835 10.1161/01.cir.98.17.1790

[R5] Antz M, Otomo K, Arruda M, Scherlag BJ, Pitha J, Tondo C, Lazzara R, Jackman WM, 1998b. Electrical conduction between the right atrium and the left atrium via the musculature of the coronary sinus. Circulation 98, 1790–1795. 10.1161/01.CIR.98.17.1790, URL: https://www.ahajournals.org/doi/10.1161/01.CIR.98.17.1790. 9788835

[R6] Augustin CM, Neic A, Liebmann M, Prassl AJ, Niederer SA, Haase G, Plank G, 2016. Anatomically accurate high resolution modeling of human whole heart electromechanics: a strongly scalable algebraic multigrid solver method for nonlinear deformation. J. Comput. Phys. 305, 622–646. 26819483 10.1016/j.jcp.2015.10.045 PMC4724941

[R7] Azzolin L, Eichenlaub M, Nagel C, Nairn D, Sánchez J, Unger L, Arentz T, Westermann D, Dössel O, Jadidi A, Loewe A, 2023. AugmentA: Patientspecific augmented atrial model generation tool. Comput. Med. Imaging Graph. 108, 102265. 10.1016/j.compmedimag.2023.102265, URL: https://linkinghub.elsevier.com/retrieve/pii/S0895611123000836. 37392493

[R8] Azzolin L, Luongo G, Ventura SR, Saiz J, Dösse O, Loewe A, 2020. Influence of gradient and smoothness of atrial wall thickness on initiation and maintenance of atrial fibrillation. In: 2020 Computing in Cardiology. IEEE, pp. 1–4.

[R9] Bachmann G, 1916. The inter-auricular time interval. Am. J. Physiol.-Legacy Content 41, 309–320. 10.1152/ajplegacy.1916.41.3.309.

[R10] Barrios Espinosa C, Sánchez J, Appel S, Becker S, Krauß J, az P, Unger L, Houillon M, Loewe A, 2025. A cyclical fast iterative method for simulating reentries in cardiac electrophysiology using an eikonal-based model. Eng. Comput. 10.1007/s00366-024-02094-9.

[R11] Batdorf M, Freitag L, Ollivier-Gooch C, Batdorf M, Freitag L, Ollivier-Gooch C, 1997. Computational study of the effect of unstructured mesh quality on solution efficiency. In: 13th Computational Fluid Dynamics Conference. p. 1888.

[R12] Bayer J, Prassl AJ, Pashaei A, Gomez JF, Frontera A, Neic A, Plank G, Vigmond EJ, 2018. Universal ventricular coordinates: A generic framework for describing position within the heart and transferring data. Med. Image Anal. 45, 83–93. 29414438 10.1016/j.media.2018.01.005

[R13] Beinart R, Abbara S, Blum A, Ferencik M, Heist K, Ruskin J, Mansour M, 2011. Left atrial wall thickness variability measured by ct scans in patients undergoing pulmonary vein isolation. J. Cardiovasc. Electrophysiol. 22, 1232–1236. 21615817 10.1111/j.1540-8167.2011.02100.x

[R14] Bhagirath P, Strocchi M, Bishop MJ, Boyle P, Plank G, 2024. From bits to bedside: Entering the age of digital twins in cardiac electrophysiology. Europace. 10.1093/europace/euae295 PMC11649999 39688585

[R15] Bishop MJ, Plank G, 2011a. Bidomain ECG simulations using an augmented monodomain model for the cardiac source. IEEE Trans. Biomed. Eng. 58, 2297–2307. 10.1109/TBME.2011.2148718 PMC3378475 21536529

[R16] Bishop MJ, Plank G, 2011b. Representing cardiac bidomain bath-loading effects by an augmented monodomain approach: application to complex ventricular models. IEEE Trans. Bio-Medical Eng. 58, 1066–1075. 10.1109/TBME.2010.2096425. PMC3075562 21292591

[R17] Bishop M, Plank G, 2025. Stochastic behaviour of arrhythmia induction in virtual heart models suggests caution in offering mechanistic insights. Nat. Cardiovasc. Res. (in press). 10.1038/s44161-025-00641-1 40269206

[R18] Bishop M, Rajani R, Plank G, Gaddum N, Carr-White G, Wright M, O’Neill M, Niederer S, 2016. Three-dimensional atrial wall thickness maps to inform catheter ablation procedures for atrial fibrillation. Europace 18, 376–383. 25842272 10.1093/europace/euv073 PMC5841557

[R19] Boyett MR, Honjo H, Kodama I, 2000. The sinoatrial node, a heterogeneous pacemaker structure. Cardiovasc. Res. 47, 658–687. 10974216 10.1016/s0008-6363(00)00135-8

[R20] Boyle PM, Zghaib T, Zahid S, Ali RL, Deng D, Franceschi WH, Hakim JB, Murphy MJ, Prakosa A, Zimmerman SL, , 2019. Computationally guided personalized targeted ablation of persistent atrial fibrillation. Nat. Biomed. Eng. 3, 870–879. 31427780 10.1038/s41551-019-0437-9 PMC6842421

[R21] van Campenhout MJ, Yaksh A, Kik C, de Jaegere PP, Ho SY, Allessie MA, de Groot NM, 2013. Bachmann’s bundle. Circ.: Arrhythmia Electrophysiol. 6, 1041–1046. 10.1161/CIRCEP.113.000758. 24129206

[R22] Chauvin M, Shah DC, Haïssaguerre M, Marcellin L, Brechenmacher C, 2000. The anatomic basis of connections between the coronary sinus musculature and the left atrium in humans. Circulation 101, 647–652. 10673257 10.1161/01.cir.101.6.647

[R23] Conley R, Delaney TJ, Jiao X, 2016. Overcoming element quality dependence of finite elements with adaptive extended stencil fem (aes-fem). Internat. J. Numer. Methods Engrg. 108, 1054–1085.

[R24] Corradi D, Maestri R, Macchi E, Callegari S, 2011. The atria: from morphology to function. J. Cardiovasc. Electrophysiol. 22, 223–235. 20812935 10.1111/j.1540-8167.2010.01887.x

[R25] Corral-Acero J, Margara F, Marciniak M, Rodero C, Loncaric F, Feng Y, Gilbert A, Fernandes JF, Bukhari HA, Wajdan A, Martinez MV, Santos MS, Shamohammdi M, Luo H, Westphal P, Leeson P, DiAchille P, Gurev V, Mayr M, Geris L, Pathmanathan P, Morrison T, Cornelussen R, Prinzen F, Delhaas T, Doltra A, Sitges M, Vigmond EJ, Zacur E, Grau V, Rodriguez B, Remme EW, Niederer S, Mortier P, McLeod K, Potse M, Pueyo E, Bueno-Orovio A, Lamata P, 2020. The ‘digital twin’ to enable the vision of precision cardiology. Eur. Heart J. 1–11. 10.1093/eurheartj/ehaa159. PMC7774470 32128588

[R26] Costa CM, Campos FO, Prassl AJ, Dos Santos RW, Sanchez-Quintana D, Ahammer H, Hofer E, Plank G, 2014. An efficient finite element approach for modeling fibrotic clefts in the heart. IEEE Trans. Biomed. Eng. 61, 900–910. 10.1109/TBME.2013.2292320. 24557691 PMC3971459

[R27] Courtemanche M, Ramirez RJ, Nattel S, 1998. Ionic mechanisms underlying human atrial action potential properties: insights from a mathematical model. Am. J. Physiol.-Heart Circ. Physiol 275, H301–H321. 10.1152/ajpheart.1998.275.1.H301 9688927

[R28] Coveney S, Roney CH, Corrado C, Wilkinson RD, Oakley JE, Niederer SA, Clayton RH, 2022. Calibrating cardiac electrophysiology models using latent gaussian processes on atrial manifolds. Sci. Rep. 12, 16572. 36195766 10.1038/s41598-022-20745-z PMC9532401

[R29] Crozier A, Augustin C, Neic A, Prassl A, Holler M, Fastl T, Hennemuth A, Bredies K, Kuehne T, Bishop M, , 2016. Image-based personalization of cardiac anatomy for coupled electromechanical modeling. Ann. Biomed. Eng. 44, 58–70. 26424476 10.1007/s10439-015-1474-5 PMC4690840

[R30] Csepe TA, Zhao J, Hansen BJ, Li N, Sul LV, Lim P, Wang Y, Simonetti OP, Kilic A, Mohler PJ, Janssen PM, Fedorov VV, 2016. Human sinoatrial node structure: 3D microanatomy of sinoatrial conduction pathways. Prog. Biophys. Mol. Biol. 120, 164–178. 10.1016/j.pbiomolbio.2015.12.011, URL: https://linkinghub.elsevier.com/retrieve/pii/S0079610715002606. 26743207 PMC4808362

[R31] Harrild David M., C.S.H., 2000. A computer model of normal conduction in the human atria. Circ. Res. 87, e25–e36. 10.1161/01.RES.87.7.e25, publisher: American Heart Association, URL: https://www.ahajournals.org/doi/10.1161/01.res.87.7.e25. 11009627

[R32] Deng D.d., Gong Y.l., Shou G.f., Jiao P.f., Zhang H.g., Ye X.s., Xia L, 2012. Simulation of biatrial conduction via different pathways during sinus rhythm with a detailed human atrial model. J. Zhejiang Univ. Sci. B 13, 676–694. 22949359 10.1631/jzus.B1100339 PMC3437366

[R33] Dewland TA, Wintermark M, Vaysman A, Smith LM, Tong E, Vittinghoff E, Marcus GM, 2013. Use of computed tomography to identify atrial fibrillation associated differences in left atrial wall thickness and density. Pacing Clin. Electrophysiol. 36, 55–62. 23106219 10.1111/pace.12028 PMC3541430

[R34] Dobrzynski H, Li J, Tellez J, Greener I, Nikolski V, Wright S, Parson S, Jones S, Lancaster M, Yamamoto M, , 2005. Computer three-dimensional reconstruction of the sinoatrial node. Circulation 111, 846–854. 15699261 10.1161/01.CIR.0000152100.04087.DB

[R35] Dössel O, Krueger MW, Weber FM, Wilhelms M, Seemann G, 2012. Computational modeling of the human atrial anatomy and electrophysiology. Med. Biol. Eng. Comput. 50, 773–799. 22718317 10.1007/s11517-012-0924-6

[R36] Fedele M, Piersanti R, Regazzoni F, Salvador M, Africa PC, Bucelli M, Zingaro A, Quarteroni A, , 2023. A comprehensive and biophysically detailed computational model of the whole human heart electromechanics. Comput. Methods Appl. Mech. Engrg. 410, 115983.

[R37] Fedorov VV, Glukhov AV, Chang R, 2012. Conduction barriers and pathways of the sinoatrial pacemaker complex: their role in normal rhythm and atrial arrhythmias. Am. J. Physiol.-Heart Circ. Physiol 302, H1773–H1783. 10.1152/ajpheart.00892.2011, URL: https://www.physiology.org/doi/10.1152/ajpheart.00892.2011. 22268110

[R38] Fedorov VV, Glukhov AV, Chang R, Kostecki G, Aferol H, Hucker WJ, Wuskell JP, Loew LM, Schuessler RB, Moazami N, Efimov IR, 2010. Optical mapping of the isolated coronary-perfused human sinus node. J. Am. Coll. Cardiol. 56, 1386–1394. 10.1016/j.jacc.2010.03.098, URL: https://linkinghub.elsevier.com/retrieve/pii/S0735109710034571. 20946995 PMC3008584

[R39] Ferrer A, Sebastián R, Sánchez-Quintana D, Rodriguez JF, Godoy EJ, Martinez L, Saiz J, 2015. Detailed anatomical and electrophysiological models of human atria and torso for the simulation of atrial activation. PloS One 10, e0141573. 26523732 10.1371/journal.pone.0141573 PMC4629897

[R40] Geselowitz DB, 1989. On the theory of the electrocardiogram. Proc. IEEE 77, 857–876.

[R41] Gillette K, Gsell MA, Bouyssier J, Prassl AJ, Neic A, Vigmond EJ, Plank G, 2021a. Automated framework for the inclusion of a his–purkinje system in cardiac digital twins of ventricular electrophysiology. Ann. Biomed. Eng. 49, 3143–3153. 34431016 10.1007/s10439-021-02825-9 PMC8671274

[R42] Gillette K, Gsell MAF, Prassl AJ, Karabelas E, Reiter U, Reiter G, Grandits T, Payer C, Štern D, Urschler M, Bayer JD, Augustin CM, Neic A, Pock T, Vigmond EJ, Plank G, 2021b. A framework for the generation of digital twins of cardiac electrophysiology from clinical 12-leads ECGs. Med. Image Anal. 71, 102080. 10.1016/j.media.2021.102080, publisher: Elsevier B.V. 33975097

[R43] Gillette K, Gsell MA, Strocchi M, Grandits T, Neic A, Manninger M, Scherr D, Roney CH, Prassl AJ, Augustin CM, , 2022. A personalized real-time virtual model of whole heart electrophysiology. Front. Physiol. 13, 907190. 36213235 10.3389/fphys.2022.907190 PMC9539798

[R44] Grandits T, Effland A, Pock T, Krause R, Plank G, Pezzuto S, 2021. GEASI: Geodesic-based earliest activation sites identification in cardiac models. Int. J. Numer. Methods Biomed. Eng. 37, e3505. 10.1002/cnm.3505, arXiv:2102.09962. PMC8459297 34170082

[R45] Grandits T, Gillette K, Plank G, Pezzuto S, 2024a. Accurate and efficient cardiac digital twin from surface ECGs: Insights into identifiability of ventricular conduction system. 10.48550/arXiv.2411.00165, arXiv:2411.00165. 40479962

[R46] Grandits T, Verhülsdonk J, Haase G, Effland A, Pezzuto S, 2024b. Digital twinning of cardiac electrophysiology models from the surface ECG: A geodesic backpropagation approach. IEEE Trans. Biomed. Eng. 71, 1281–1288. 10.1109/TBME.2023.3331876, URL: https://ieeexplore.ieee.org/document/10339854, conference Name: IEEE Transactions on Biomedical Engineering. 38048238

[R47] Gray RA, Pertsov AM, Jalife J, 1996. Incomplete reentry and epicardial breakthrough patterns during atrial fibrillation in the sheep heart. Circulation 94, 2649–2661. 8921813 10.1161/01.cir.94.10.2649

[R48] Gsell MA, Neic A, Bishop MJ, Gillette K, Prassl AJ, Augustin CM, Vigmond EJ, Plank G, 2024. ForCEPSS—A framework for cardiac electrophysiology simulations standardization. Comput. Methods Programs Biomed. 251, 108189. 10.1016/j.cmpb.2024.108189, URL: https://linkinghub.elsevier.com/retrieve/pii/S0169260724001858. 38728827

[R49] Hansson A, Holm M, Blomström P, Johansson R, Lührs C, Brandt J, Olsson S, 1998. Right atrial free wall conduction velocity and degree of anisotropy in patients with stable sinus rhythm studied during open heart surgery. Eur. Heart J. 19, 293–300. 9519324 10.1053/euhj.1997.0742

[R50] Harrild DM, Henriquez CS, 2000. A computer model of normal conduction in the human atria. Circ. Res. 87, e25–e36. 11009627 10.1161/01.res.87.7.e25

[R51] Ho S, Sánchez-Quintana D, 2009. The importance of atrial structure and fibers. Clin. Anat.: Off. J. Am. Assoc. Clin. Anat. Br. Assoc. Clin. Anat. 22, 52–63. 10.1002/ca.20634 18470938

[R52] Honjo H, Boyett M, Kodama I, Toyama J, 1996. Correlation between electrical activity and the size of rabbit sino-atrial node cells. J. Physiol. 496, 795–808. 8930845 10.1113/jphysiol.1996.sp021728 PMC1160865

[R53] Hopman LH, Visch JE, Bhagirath P, van der Laan AM, Mulder MJ, Razeghi O, Kemme MJ, Niederer SA, Allaart CP, Götte MJ, 2023. Right atrial function and fibrosis in relation to successful atrial fibrillation ablation. Eur. Hear. J.-Cardiovasc. Imaging 24, 336–345. 10.1093/ehjci/jeac152 PMC9936834 35921538

[R54] Joyner R, Van Capelle F, 1986. Propagation through electrically coupled cells. How a small SA node drives a large atrium. Biophys. J. 50, 1157–1164. 10.1016/S0006-3495(86)83559-7, URL: https://linkinghub.elsevier.com/retrieve/pii/S0006349586835597. 3801575 PMC1329789

[R55] Kanchi H, Masud A, 2007. A 3d adaptive mesh moving scheme. Internat. J. Numer. Methods Fluids 54, 923–944.

[R56] Karabelas E, Gsell MAF, Augustin CM, Marx L, Neic A, Prassl AJ, Goubergrits L, Kuehne T, Plank G, 2018. Towards a computational framework for modeling the impact of aortic coarctations upon left ventricular load. Front. Physiol 9 (538), 10.3389/fphys.2018.00538, URL: http://www.ncbi.nlm.nih.gov/pubmed/29892227. PMC5985756 29892227

[R57] Keith A, Flack M, 1907. The form and nature of the muscular connections between the primary divisions of the vertebrate heart. J. Anat. Physiol. 41, 172. 17232727 PMC1289112

[R58] Keller DU, Weber FM, Seemann G, Dossel O, 2010. Ranking the influence of tissue conductivities on forward-calculated ecgs. IEEE Trans. Biomed. Eng. 57, 1568–1576. 20659824 10.1109/TBME.2010.2046485

[R59] Kharbanda RK, Özdemir EH, Taverne YJ, Kik C, Bogers AJ, de Groot NM, 2019. Current concepts of anatomy, electrophysiology, and therapeutic implications of the interatrial septum. JACC: Clin. Electrophysiol. 5, 647–656. 31221350 10.1016/j.jacep.2019.04.013

[R60] Knol WG, Teuwen CP, Kleinrensink GJ, Bogers AJ, de Groot NM, Taverne YJ, 2019. The bachmann bundle and interatrial conduction: comparing atrial morphology to electrical activity. Hear. Rhythm. 16, 606–614. 10.1016/j.hrthm.2018.10.021 30366158

[R61] Knupp P, 2022. Worst case mesh quality in the target matrix optimization paradigm. Eng. Comput. 38, 5695–5711.

[R62] Krueger MW, Seemann G, Rhode K, Keller DU, Schilling C, Arujuna A, Gill J, O’Neill MD, Razavi R, Dossel O, 2012. Personalization of atrial anatomy and electrophysiology as a basis for clinical modeling of radio-frequency ablation of atrial fibrillation. IEEE Trans. Med. Imaging 32, 73–84. 22665507 10.1109/TMI.2012.2201948

[R63] Kucherenko S, Albrecht D, Saltelli A, 2015. Exploring multi-dimensional spaces: a comparison of latin hypercube and quasi Monte Carlo sampling techniques. arXiv:1505.02350.

[R64] Labarthe S, Bayer J, Coudiere Y, Henry J, Cochet H, Jais P, Vigmond E, 2014. A bilayer model of human atria: mathematical background, construction, and assessment. Europace 16, iv21–iv29. 10.1093/europace/euu256, URL: https://academic.oup.com/europace/article-lookup/doi/10.1093/europace/euu256. 25362166

[R65] Lang RM, Cameli M, Sade LE, Faletra FF, Fortuni F, Rossi A, Soulat-Dufour L, 2022. Imaging assessment of the right atrium: anatomy and function. Eur. Hear. J.-Cardiovasc. Imaging 23, 867–884. 10.1093/ehjci/jeac011 35079782

[R66] Lemery R, Birnie D, Tang AS, Green M, Gollob M, Hendry M, Lau E, 2007. Normal atrial activation and voltage during sinus rhythm in the human heart: an endocardial and epicardial mapping study in patients with a history of atrial fibrillation. J. Cardiovasc. Electrophysiol. 18, 402–408. 17394455 10.1111/j.1540-8167.2007.00762.x

[R67] Lemery R, Guiraudon G, Veinot JP, 2003. Anatomic description of bachmann’s bundle and its relation to the atrial septum. Am. J. Cardiol. 91, 1482–1482. 12804741 10.1016/s0002-9149(03)00405-3

[R68] Li N, Hansen BJ, Csepe TA, Zhao J, Ignozzi AJ, Sul LV, Zakharkin SO, Kalyanasundaram A, Davis JP, Biesiadecki BJ, Kilic A, Janssen PML, Mohler PJ, Weiss R, Hummel JD, Fedorov VV, 2017. Redundant and diverse intranodal pacemakers and conduction pathways protect the human sinoatrial node from failure. Sci. Transl. Med. 9, eaam5607. 10.1126/scitranslmed.aam5607, URL: https://www.science.org/doi/10.1126/scitranslmed.aam5607. 28747516 PMC5775890

[R69] Loewe A, Krueger MW, Holmqvist F, Dössel O, Seemann G, Platonov PG, 2016. Influence of the earliest right atrial activation site and its proximity to interatrial connections on p-wave morphology. EP Eur 18, iv35–iv43. 10.1093/europace/euw349 28011829

[R70] Loewe A, Krueger MW, Platonov PG, Holmqvist F, Dössel O, Seemann G, 2015. Left and right atrial contribution to the p-wave in realistic computational models. In: Functional Imaging and Modeling of the Heart: 8th International Conference, FIMH 2015, Maastricht, the Netherlands, June (2015) 25–27. Proceedings 8. Springer, pp. 439–447.

[R71] Lubrecht JM, Grandits T, Gharaviri A, Schotten U, Pock T, Plank G, Krause R, Auricchio A, Conte G, Pezzuto S, 2021. Automatic reconstruction of the left atrium activation from sparse intracardiac contact recordings by inverse estimate of fibre structure and anisotropic conduction in a patient-specific model. EP Eur. 23, i63–i70. 10.1093/europace/euaa392, URL: https://academic.oup.com/europace/article/23/Supplement_1/i63/6158558. 33751078

[R72] Monfredi O, Dobrzynski H, Mondal T, Boyett MR, Morris GM, 2010. The anatomy and physiology of the sinoatrial node—a contemporary review. Pacing Clin. Electrophysiol. 33, 1392–1406. 20946278 10.1111/j.1540-8159.2010.02838.x

[R73] Multerer M, Pezzuto S, 2021. Uncertainty quantification for the 12-lead ecg: a lead field approach. arXiv preprint arXiv:2102.09960.

[R74] Muñoz MA, Kaur J, Vigmond EJ, 2011. Onset of atrial arrhythmias elicited by autonomic modulation of rabbit sinoatrial node activity: a modeling study. Am. J. Physiol.-Heart Circ. Physiol 301, H1974–H1983. 10.1152/ajpheart.00059.2011. 21856904 PMC3774554

[R75] Nagel C, Espinosa CB, Gillette K, Gsell MA, Sánchez J, Plank G, Dössel O, Loewe A, 2022. Comparison of propagation models and forward calculation methods on cellular, tissue and organ scale atrial electrophysiology. IEEE Trans. Biomed. Eng. 70, 511–522. 10.1109/TBME.2022.3196144 35921339

[R76] Nagel C, Schuler S, Dössel O, Loewe A, 2021. A bi-atrial statistical shape model for large-scale in silico studies of human atria: Model development and application to ECG simulations. Med. Image Anal. 74, 102210. 10.1016/j.media.2021.102210, URL: https://linkinghub.elsevier.com/retrieve/pii/S1361841521002553. 34450467

[R77] Neic A, Campos FO, Prassl AJ, Neiderer SA, Bishop MJ, Vigmond EJ, Plank G, 2017a. Efficient computation of electrograms and ecgs in human whole heart simulations using a reaction-eikonal model. 10.1016/j.jcp.2017.06.020 PMC5555399 28819329

[R78] Neic A, Campos FO, Prassl AJ, Niederer SA, Bishop MJ, Vigmond EJ, Plank G, 2017b. Efficient computation of electrograms and ecgs in human whole heart simulations using a reaction-eikonal model. J. Comput. Phys. 346, 191–211. 10.1016/j.jcp.2017.06.020. 28819329 PMC5555399

[R79] Neic A, Gsell MA, Karabelas E, Prassl AJ, Plank G, 2020. Automating image-based mesh generation and manipulation tasks in cardiac modeling workflows using meshtool. SoftwareX 11, 100454. 32607406 10.1016/j.softx.2020.100454 PMC7326605

[R80] Niederer SA, Aboelkassem Y, Cantwell CD, Corrado C, Coveney S, Cherry EM, Delhaas T, Fenton FH, Panfilov AV, Pathmanathan P, Plank G, Riabiz M, Roney CH, Dos Santos RW, Wang L, 2020. Creation and application of virtual patient cohorts of heart models. Philos. Trans. A Math. Phys. Eng. Sci. 378, 20190558. 10.1098/rsta.2019.0558. 32448064 PMC7287335

[R81] Padala SK, Cabrera JA, Ellenbogen KA, 2021. Anatomy of the cardiac conduction system. Pacing Clin. Electrophysiol. 44, 15–25. 33118629 10.1111/pace.14107

[R82] Payer C, Štern D, Bischof H, Urschler M, 2017. Multi-label whole heart segmentation using cnns and anatomical label configurations. In: International Workshop on Statistical Atlases and Computational Models of the Heart. Springer, pp. 190–198.

[R83] Pezzuto S, Kal’avský P, Potse M, Prinzen FW, Auricchio A, Krause R, 2017. Evaluation of a rapid anisotropic model for ECG simulation. Front. Physiol 8, 10.3389/fphys.2017.00265. PMC5411438 28512434

[R84] Piersanti R, Africa PC, Fedele M, Vergara C, Dedè L, Corno AF, Quarteroni A, 2021. Modeling cardiac muscle fibers in ventricular and atrial electrophysiology simulations. Comput. Methods Appl. Mech. Engrg. 373, 113468. 10.1016/j.cma.2020.113468, publisher: Elsevier B.V..

[R85] Plank G, Loewe A, Neic A, Augustin C, Huang YL, Gsell MA, Karabelas E, Nothstein M, Prassl AJ, Sánchez J, , 2021. The opencarp simulation environment for cardiac electrophysiology. Comput. Methods Programs Biomed. 208, 106223. 34171774 10.1016/j.cmpb.2021.106223

[R86] Platonov PG, 2007. Interatrial conduction in the mechanisms of atrial fibrillation: from anatomy to cardiac signals and new treatment modalities. Europace 9, vi10–vi16. 17959684 10.1093/europace/eum201

[R87] Platonov P, Mitrofanova L, Chireikin L, Olsson S, 2002. Morphology of inter-atrial conduction routes in patients with atrial fibrillation. Europace 4, 183–192. 12135252 10.1053/eupc.2002.0221

[R88] Potse M, 2018. Scalable and accurate ECG simulation for reaction–diffusion models of the human heart. Front. Physiol. 9, 370. 29731720 10.3389/fphys.2018.00370 PMC5920200

[R89] Potse M, Dubé B, Richer J, Vinet A, Gulrajani RM, 2006. A comparison of monodomain and bidomain reaction–diffusion models for action potential propagation in the human heart. IEEE Trans. Biomed. Eng. 53, 2425–2435. 17153199 10.1109/TBME.2006.880875

[R90] Prassl AJ, Kickinger F, Ahammer H, Grau V, Schneider JE, Hofer E, Vigmond EJ, Trayanova NA, Plank G, 2009. Automatically generated, anatomically accurate meshes for cardiac electrophysiology problems. IEEE Trans. Bio-Medical Eng. 56, 1318–1330. 10.1109/TBME.2009.2014243. PMC2819345 19203877

[R91] Reddy VY, Kong MH, Petru J, Maan A, Funasako M, Minami K, Ruppersberg P, Dukkipati S, Neuzil P, 2023. Electrographic flow mapping of persistent atrial fibrillation: intra-and inter-procedure reproducibility in the absence of ‘ground truth’. Europace 25, euad308. 37956309 10.1093/europace/euad308 PMC10642765

[R92] Roberts DE, Hersh LT, Scher AM, 1979. Influence of cardiac fiber orientation on wavefront voltage, conduction velocity, and tissue resistivity in the dog. Circ. Res. 44, 701–712. 428066 10.1161/01.res.44.5.701

[R93] Rodero C, Strocchi M, Marciniak M, Longobardi S, Whitaker J, O’Neill MD, Gillette K, Augustin C, Plank G, Vigmond EJ, Lamata P, Niederer SA, 2021. Linking statistical shape models and simulated function in the healthy adult human heart. PLoS Comput. Biol. 17, e1008851. 10.1371/journal.pcbi.1008851. 33857152 PMC8049237

[R94] Roney CH, Bendikas R, Pashakhanloo F, Corrado C, Vigmond EJ, McVeigh ER, Trayanova NA, Niederer SA, 2021. Constructing a human atrial fibre atlas. Ann. Biomed. Eng. 49, 233–250. 32458222 10.1007/s10439-020-02525-w PMC7773625

[R95] Roney CH, Pashaei A, Meo M, Dubois R, Boyle PM, Trayanova NA, Cochet H, Niederer SA, Vigmond EJ, 2019a. Universal atrial coordinates applied to visualisation, registration and construction of patient specific meshes. Med. Image Anal. 55, 65–75. 10.1016/j.media.2019.04.004, arXiv:1810.06630. 31026761 PMC6543067

[R96] Roney CH, Pashaei A, Meo M, Dubois R, Boyle PM, Trayanova NA, Cochet H, Niederer SA, Vigmond EJ, 2019b. Universal atrial coordinates applied to visualisation, registration and construction of patient specific meshes. Med. Image Anal. 55, 65–75. 10.1016/j.media.2019.04.004, URL: https://www.sciencedirect.com/science/article/pii/S1361841518308089. 31026761 PMC6543067

[R97] Roney C, Solis Lemus JA, , 2023. Constructing bilayer and volumetric atrial models at scale. Interface Focus. 20230038. 10.1098/rsfs.2023.0038 PMC10722212 38106921

[R98] Sakamoto SI, Nitta T, Ishii Y, Miyagi Y, Ohmori H, Shimizu K, 2005. Interatrial electrical connections: the precise location and preferential conduction. J. Cardiovasc. Electrophysiol. 16, 1077–1086. 16191118 10.1111/j.1540-8167.2005.40659.x

[R99] Sakata K, Bradley RP, Prakosa A, Yamamoto CAP, Ali SY, Loeffler S, Tice BM, Boyle PM, Kholmovski EG, Yadav R, Sinha SK, Marine JE, Calkins H, Spragg DD, Trayanova NA, 2024. Assessing the arrhythmogenic propensity of fibrotic substrate using digital twins to inform a mechanisms-based atrial fibrillation ablation strategy. Nat. Cardiovasc. Res. 3, 857–868. 10.1038/s44161-024-00489-x, publisher: Nature Publishing Group, URL: https://www.nature.com/articles/s44161-024-00489-x. 39157719 PMC11329066

[R100] Schneider T, Hu Y, Dumas J, Gao X, Panozzo D, Zorin D, 2018. Decoupling simulation accuracy from mesh quality. ACM Trans. Graph.

[R101] Schotten U, Verheule S, Kirchhof P, Goette A, 2011. Pathophysiological mechanisms of atrial fibrillation: a translational appraisal. Physiol. Rev. 91, 265–325. 21248168 10.1152/physrev.00031.2009

[R102] Schuler S, Pilia N, Potyagaylo D, Loewe A, 2021. Cobiveco: Consistent biventricular coordinates for precise and intuitive description of position in the heart–with matlab implementation. Med. Image Anal. 74, 102247. 34592711 10.1016/j.media.2021.102247

[R103] Schuler S, Tate JD, Oostendorp TF, MacLeod RS, Dössel O, 2019. Spatial downsampling of surface sources in the forward problem of electrocardiography. In: Coudière Y, Ozenne V, Vigmond E, Zemzemi N (Eds.), Functional Imaging and Modeling of the Heart. Springer International Publishing, Cham, pp. 29–36. 10.1007/978-3-030-21949-9_4.

[R104] Seemann G, Höper C, Sachse FB, Dössel O, Holden AV, Zhang H, 2006. Heterogeneous three-dimensional anatomical and electrophysiological model of human atria. Philos. Trans. Ser. A Math. Phys. Eng. Sci. 364, 1465–1481. 10.1098/rsta.2006.1781, URL: http://www.ncbi.nlm.nih.gov/pubmed/16766355. 16766355

[R105] Szili-Torok T, Neuzil P, Langbein A, Petru J, Funasako M, Dinshaw L, Wijchers S, Bhagwandien R, Rillig A, Spitzer SG, , 2023. Electrographic flow–guided ablation in redo patients with persistent atrial fibrillation (flow-af): design and rationale. Hear. Rhythm. O2 4, 391–400. 10.1016/j.hroo.2023.04.001 PMC10287997 37361617

[R106] Matsuyama T.a., Inoue S, Kobayashi Y, Sakai T, Saito T, Katagiri T, Ota H, 2004. Anatomical diversity and age-related histological changes in the human right atrial posterolateral wall. EP Eur. 6, 307–315. 10.1016/j.eupc.2004.03.011 15172655

[R107] Tan K, Chan K, Choi K, 2000. Detection of the qrs complex, p wave and t wave in electrocardiogram. In: 2000 First International Conference Advances in Medical Signal and Information Processing (IEE Conf. Publ. No. 476). IET, pp. 41–47.

[R108] Thaler F, Payer C, Bischof H, Stern D, 2021. Efficient multi-organ segmentation using spatial configuration-net with low gpu memory requirements. arXiv preprint arXiv:2111.13630.

[R109] Varela M, Morgan R, Theron A, Dillon-Murphy D, Chubb H, Whitaker J, Henningsson M, Aljabar P, Schaeffter T, Kolbitsch C, , 2017. Novel mri technique enables non-invasive measurement of atrial wall thickness. IEEE Trans. Med. Imaging 36, 1607–1614. 28422654 10.1109/TMI.2017.2671839 PMC5549842

[R110] Viceconti M, Pappalardo F, Rodriguez B, Horner M, Bischoff J, Musuamba Tshinanu F, 2020. In silico trials: Verification, validation and uncertainty quantification of predictive models used in the regulatory evaluation of biomedical products. Methods 10.1016/j.ymeth.2020.01.011. PMC7883933 31991193

[R111] Vigmond EJ, Clements C, 2007. Construction of a computer model to investigate sawtooth effects in the purkinje system. IEEE Trans. Biomed. Eng. 54, 389–399. 10.1109/TBME.2006.888817, (Print)\r0018–9294 (Linking), 17355050

[R112] Wachter A, Loewe A, Krueger MW, Dössel O, Seemann G, 2015. Mesh structure-independent modeling of patient-specific atrial fiber orientation. Curr. Dir. Biomed. Eng. 1, 409–412.

[R113] Whitaker J, Rajani R, Chubb H, Gabrawi M, Varela M, Wright M, Niederer S, O’Neill MD, 2016. The role of myocardial wall thickness in atrial arrhythmogenesis. Ep Eur. 18, 1758–1772. 10.1093/europace/euw014 PMC5841556 27247007

[R114] Whittaker DG, Clerx M, Lei CL, Christini DJ, Mirams GR, 2020. Calibration of ionic and cellular cardiac electrophysiology models. Wiley Interdiscip. Rev.: Syst. Biol. Med 12, e1482. 32084308 10.1002/wsbm.1482 PMC8614115

[R115] Yang J, Li H, Campbell D, Jia Y, 2015. Go-icp: A globally optimal solution to 3d icp point-set registration. IEEE Trans. Pattern Anal. Mach. Intell. 38, 2241–2254. 26731638 10.1109/TPAMI.2015.2513405

[R116] Yang J, Li H, Jia Y, 2013. Go-icp: Solving 3d registration efficiently and globally optimally. In: Proceedings of the IEEE International Conference on Computer Vision. pp. 1457–1464.

[R117] Yushkevich PA, Piven J, Cody Hazlett H, Gimpel Smith R, Ho S, Gee JC, Gerig G, 2006. User-guided 3D active contour segmentation of anatomical structures: Significantly improved efficiency and reliability. Neuroimage 31, 1116–1128. 16545965 10.1016/j.neuroimage.2006.01.015

[R118] Zappon E, Manzoni A, Quarteroni A, 2024. A non-conforming-in-space numerical framework for realistic cardiac electrophysiological outputs. J. Comput. Phys. 502, 112815. 10.1016/j.jcp.2024.112815, URL: https://www.sciencedirect.com/science/article/pii/S0021999124000640.

[R119] Zheng T, Azzolin L, Sánchez J, Dössel O, Loewe A, 2021. An automate pipeline for generating fiber orientation and region annotation in patient specific atrial models. Curr. Dir. Biomed. Eng. 7, 136–139. 10.1515/cdbme-2021-2035.

